# Common data model for COVID-19 datasets

**DOI:** 10.1093/bioinformatics/btac651

**Published:** 2022-10-27

**Authors:** Philipp Wegner, Geena Mariya Jose, Vanessa Lage-Rupprecht, Sepehr Golriz Khatami, Bide Zhang, Stephan Springstubbe, Marc Jacobs, Thomas Linden, Cindy Ku, Bruce Schultz, Martin Hofmann-Apitius, Alpha Tom Kodamullil, for the COPERIMOplus Consortium

**Affiliations:** Department of Bioinformatics, Fraunhofer Institute for Algorithms and Scientific Computing (SCAI), Sankt Augustin, 53757, Germany; Causality Biomodels, Kinfra Hi-Tech Park, Cochin, Kerala 683503, India; Department of Bioinformatics, Fraunhofer Institute for Algorithms and Scientific Computing (SCAI), Sankt Augustin, 53757, Germany; Department of Bioinformatics, Fraunhofer Institute for Algorithms and Scientific Computing (SCAI), Sankt Augustin, 53757, Germany; Department of Bioinformatics, Fraunhofer Institute for Algorithms and Scientific Computing (SCAI), Sankt Augustin, 53757, Germany; Department of Bioinformatics, Fraunhofer Institute for Algorithms and Scientific Computing (SCAI), Sankt Augustin, 53757, Germany; Department of Bioinformatics, Fraunhofer Institute for Algorithms and Scientific Computing (SCAI), Sankt Augustin, 53757, Germany; Department of Bioinformatics, Fraunhofer Institute for Algorithms and Scientific Computing (SCAI), Sankt Augustin, 53757, Germany; Department of Bioinformatics, Fraunhofer Institute for Algorithms and Scientific Computing (SCAI), Sankt Augustin, 53757, Germany; Department of Bioinformatics, Fraunhofer Institute for Algorithms and Scientific Computing (SCAI), Sankt Augustin, 53757, Germany; Department of Bioinformatics, Fraunhofer Institute for Algorithms and Scientific Computing (SCAI), Sankt Augustin, 53757, Germany; Bonn-Aachen International Center for IT (B-IT), Rheinische Friedrich-Wilhelms-Universität Bonn, Bonn 53115, Germany; Department of Bioinformatics, Fraunhofer Institute for Algorithms and Scientific Computing (SCAI), Sankt Augustin, 53757, Germany; Bonn-Aachen International Center for IT (B-IT), Rheinische Friedrich-Wilhelms-Universität Bonn, Bonn 53115, Germany

## Abstract

**Motivation:**

A global medical crisis like the coronavirus disease 2019 (COVID-19) pandemic requires interdisciplinary and highly collaborative research from all over the world. One of the key challenges for collaborative research is a lack of interoperability among various heterogeneous data sources. Interoperability, standardization and mapping of datasets are necessary for data analysis and applications in advanced algorithms such as developing personalized risk prediction modeling.

**Results:**

To ensure the interoperability and compatibility among COVID-19 datasets, we present here a common data model (CDM) which has been built from 11 different COVID-19 datasets from various geographical locations. The current version of the CDM holds 4639 data variables related to COVID-19 such as basic patient information (*age, biological sex* and *diagnosis*) as well as disease-specific data variables, for example, *Anosmia* and *Dyspnea.* Each of the data variables in the data model is associated with specific data types, variable mappings, value ranges, data units and data encodings that could be used for standardizing any dataset. Moreover, the compatibility with established data standards like OMOP and FHIR makes the CDM a well-designed CDM for COVID-19 data interoperability.

**Availability and implementation:**

The CDM is available in a public repo here: https://github.com/Fraunhofer-SCAI-Applied-Semantics/COVID-19-Global-Model.

**Supplementary information:**

Supplementary data are available at *Bioinformatics* online.

## 1. Introduction

An enormous amount of data has been produced since the beginning of the coronavirus disease 2019 (COVID-19) pandemic from different research groups from all over the globe. Clinical research benefits from this international effort as patient data are now available for advanced analysis with AI for applications such as personalized risk prediction modeling or early stratification of patients in the care units based on their data patterns. However, one downside is that the available clinical or patient data is highly heterogeneous, in a non-standardized format, not -interoperable, and non-comparable. This presents data scientists with the problem that the data are available but cannot be unified and readily applied to algorithms.

Such a problem highlights the need for a global common data model (CDM) for COVID-19 which is suitable for both the standardization and normalization of COVID-19 clinical datasets. Establishing such a standardization workflow via a CDM and suitable software components can provide data scientists with larger cohort sizes thus leading to better results in their data analyses. To visualize this, imagine a scenario in which two different datasets compiled from separate clinical trials observing COVID-19 patients. Each group is investigating the same hypothesis but neither can achieve statistical significance with their given sample sizes. If we are now capable of unifying the two cohorts by standardizing and normalizing them with a common model, we now might have enough data to reject a certain hypothesis. For a detailed example see Supplementary Text S2.

In the context of the COPERIMO Plus (https://www.scai.fraunhofer.de/de/geschaeftsfelder/bioinformatik/projekte/coperimoplus.html) project, we developed the COVID-19 CDM that can be used for standardizing and unifying global COVID-19 datasets. This CDM was built based on the GECCO—German Corona Consensus Dataset (Sass et al., 2020)—and extended with 10 other datasets collected globally. The CDM, in combination with the Data Steward Tool (DST) (https://doi.org/10.1093/bioinformatics/btac375) (Wegner et al., 2021), forms an end-to-end data standardization pipeline that can read data, standardize it with a common data standard (CDM) and then export it to well-established health IT formats such as FHIR via RESTful interfaces. Throughout the development, we used the DST to map data from different sources to (i) unify and standardize datasets to standard terminologies and ontologies, (ii) to further enrich the CDM with variable mappings and (iii) to map and compare with other global data standards like OMOP (https://www.ohdsi.org/data-standardization/the-common-data-model/).

## 2. Methodology

**Fig. 1.**
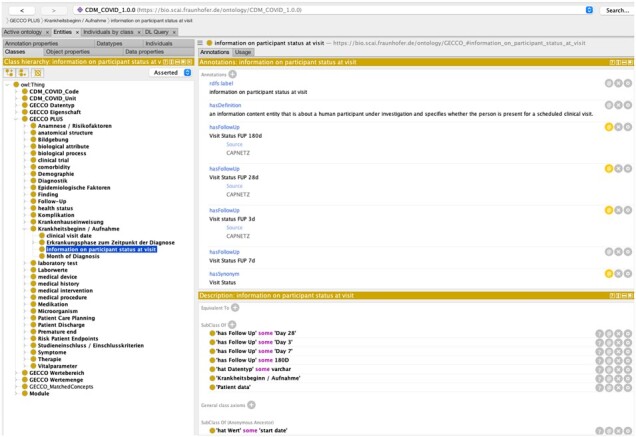
Screenshot from CDM showing various meta-data associated with a variable ‘Bilirubin’

Data elements and variables were collected from MC-19 (Lippi et al., 2020), MMIC-III (https://doi.org/10.1038/sdata.2016.35) (Johnson et al., 2016), CAPNETZ (https://www.capnetz.de/html/capnetz/project) (Capnetz Stiftung), LEOSS (Jakob et al., 2021), PaCOVID (Kurth et al., 2020), IBM Explorys Dataset (https://www.ibm.com/watson-health/about/explorys) (IBM Explorys Solutions) and datasets from various hospitals (UK Aachen, Universitätsklinikum Frankfurt, UK Erlangen) (Supplementary Table S1). The list of overlapping entities from these various datasets is given in Supplementary Table S2. The current version of the COVID-19 CDM consists of 4895 total concepts, among which 4639 are the commonly used data variables in the clinical context of SARS-CoV-2 research. For each data variable, the model provides further information such as data types, value ranges, data encodings and data units (Fig. 1). The set of variables itself has an internal hierarchy with 32 top-level categories including *diagnostics* and *medication* leading down to lower-level groupings that distinguish, for example, computed *tomography results* and *X-Ray findings.* Additionally, the model contains information for around 9000 variable synonyms as well as alternative definitions from other data resources.

The CDM is available in both OWL and Excel formats. The excel sheet holds a subset of information about the model as it does not store the inner hierarchy of the CDM but is enough to be readable by the data steward or standardization tools. In the development phase, we worked on the .owl file and used Protégé (https://protege.stanford.edu/) to populate the model. This has several advantages since Protégé and the built-in reasoner system keep track of the models' consistency and results in an .owl file that can be read by semantic systems. After setting up a dedicated COVID-19 instance of the DST and equipping it with the newest version of the CDM, we have a data standardization solution for COVID-19 data and subsequently used it to harmonize and map SARS-CoV-2 clinical datasets and improve the interoperability.

## 3. Results

### 3.1 Data standardization of COVID-19 datasets

The CDM presented in this article adapts data variables from 11 data resources and unifies those to a common consensus to create a COVID-19 data standard. In order to validate the usability of the CDM, we mapped data variables from three major COVID-19 trials (LEOSS, MC-19 and MMIC-III) using DST onto it (Supplementary Text S3). We obtained ∼70%, ∼80% and ∼90% using automatic mapping of original data points to the standard variable name of the CDM for the LEOSS, MC-19, and MMIC-III datasets, respectively. With those variable mappings, we are able to unify those three cohorts into one large cohort that is ready for further data exploration. This resulted in 383 variable mappings and adding further mappings will only increase this value.

### 3.2 Alignment with global data models and export to FHIR

In order to migrate our solution into the current semantic data research landscape, we aligned our model with well-established global data standards. For instance, we mapped the CDM to OMOP (https://www.ohdsi.org/data-standardization/the-common-data-model/) where approximately half of the mappings (46%) could be found automatically by fuzzy string matching. An expert manual curation increased the mapping to 79%. The mapping table can be found in Supplementary Table S3 (without no-matches) and the complete table (with no-matches) can be found in Supplementary Document S2. Note that other work in the field of modeling COVID-19 data often also connects their solution to the OMOP standard (Supplementary Text S4). Additionally, in combination with the DST, we provide FHIR (https://www.hl7.org/fhir/) exports of the standardized data. Exported as a .json file with each datapoint encoded as an observation using the FHIR standard, the data can then be moved to other systems in a convenient manner. The FHIR exports can be accessed via several RESTful endpoints whose documentation can be found in the official repository of the DST.

## 4. Discussion and future work

Both the number and accuracy of the insights we are able to extract from data with advanced data analysis algorithms are crucially dependent on the size of the dataset or the number of observations a clinical dataset consists of. With the CDM presented here, we have standardized 10 datasets and unified 3 COVID-19 cohorts to provide researchers with the large datasets needed for high-precision analysis. To be noted here is, that the presented data model is not intended to replace established data models but to extend current solutions with very detailed modeling in the COVID-19 domain. Even though this CDM serves as a basis to standardize and map various heterogeneous datasets in the COVID-19 context, it is also important to note that it constantly needs to be updated with new data as our understanding of the COVID-19 disease continues to evolve. With each new dataset, there could be new variables that will need to be mapped onto the CDM. As of now, the DST’s mapping assistant suggests mappings based on fuzzy string matching and synonym lists. This has its limitations due to distinct variables with very similar naming and insufficient synonym information. Moreover, highly complex variables with potential semantic equivalent counterparts in other models will not be identified by fuzzy matching. Thus, further work will extend this approach by more sophisticated machine learning-based variable matching.

## Funding

This research was conducted in the context of the ‘COPERIMO*plus*’ initiative and supported by the Fraunhofer ‘Internal Programs Fraunhofer vs Corona’ [Anti-Corona 840266].

*Conflict of Interest*: none declared.

## Supplementary Material

btac651_Supplementary_Data
