# Supplementary material for: Common data model for COVID-19 datasets
Source: Bioinformatics. 2022 Oct 27;38(24):5466–8. doi: 10.1093/bioinformatics/btac651 (PMC9750115; doi:10.1093/bioinformatics/btac651)
Supplement: btac651_Supplementary_Data [file btac651_supplementary_data.zip › btac651_Supplementary_Data/Supplementary File GECCO++-2.pdf]

# Supplementary File

*Common Data Model for COVID-19 data*

**Supplementary Table 1: Variable Synonyms examples**

| CDM Naming | Alternative Term | Source |
|---|---|---|
| chest computed tomography results | Chest CT results | LEOSS |
| clinical visit date | Visit Date Baseline | CAPNETZ |
| immunization date | immunization_date | IBM Explorys Therapeutic Datasets |

**Supplementary Table 2: Overlappings between Sources**

| Entity | Number of datasets in which same entity is present (as synonym/exactMatch/ relatedMatch.. etc) | Datasets in which the entity is present |
|---|---|---|
| 'Respiratory Rate' | 10 | GECCO \| CAPNETZ \| MC-19 \| Frankfurt \| Erlangen \| LEOSS \| PaCovid \| Aachen \| Brazilian Variables \| MIMIC-III |
| 'Aspartate Aminotransferase' | 9 | GECCO \| CAPNETZ \| MC-19 \| Frankfurt \| Erlangen \| LEOSS \| PaCovid \| Aachen \| MIMIC-III |
| 'biological sex' | 9 | GECCO \| CAPNETZ \| MC-19 \| LEOSS \| PaCovid \| Aachen \| Brazilian Variables \| MIMIC-III \| IBM Explorys |
| 'Body Temperature' | 9 | GECCO \| CAPNETZ \| |

| | | |
|---|---|---|
| | | MC-19 \| Frankfurt \| Erlangen \| LEOSS \| PaCovid \| Brazilian Variables \| MIMIC-III |
| 'Fraction of Inspired Oxygen' | 9 | GECCO \| CAPNETZ \| MC-19 \| Frankfurt \| Erlangen \| LEOSS \| PaCovid \| Aachen \| MIMIC-III |
| hemoglobin | 9 | GECCO \| CAPNETZ \| MC-19 \| Frankfurt \| Erlangen \| LEOSS \| PaCovid \| Aachen \| MIMIC-III |
| 'Lactate Dehydrogenase' | 9 | GECCO \| CAPNETZ \| MC-19 \| Frankfurt \| Erlangen \| LEOSS \| PaCovid \| Aachen \| MIMIC-III |
| leukocyte | 9 | GECCO \| CAPNETZ \| MC-19 \| Frankfurt \| Erlangen \| LEOSS \| PaCovid \| Aachen \| MIMIC-III |
| 'partial pressure of blood oxygen (Po2)' | 9 | GECCO \| CAPNETZ \| MC-19 \| Frankfurt \| Erlangen \| LEOSS \| PaCovid \| Aachen \| MIMIC-III |
| platelet | 9 | GECCO \| CAPNETZ \| MC-19 \| Frankfurt \| Erlangen \| LEOSS \| PaCovid \| Aachen \| MIMIC-III |
| 'Alanine Aminotransferase' | 8 | CAPNETZ \| MC-19 \| Frankfurt \| Erlangen \| LEOSS \| PaCovid \| Aachen \| MIMIC-III |
| 'C-Reactive Protein' | 8 | GECCO \| CAPNETZ \| MC-19 \| Erlangen \| LEOSS \| PaCovid \| Aachen \| MIMIC-III |
| Creatinine | 8 | GECCO \| CAPNETZ \| MC-19 \| Erlangen \| LEOSS \| PaCovid \| Aachen \| MIMIC-III |
| 'diastolic blood pressure' | 8 | GECCO \| CAPNETZ \| |

| | | |
|---|---|---|
| | | MC-19 \| Erlangen \| LEOSS \| PaCovid \| Aachen \| Brazilian Variables |
| lymphocyte | 8 | GECCO \| CAPNETZ \| MC-19 \| Erlangen \| LEOSS \| PaCovid \| Aachen \| MIMIC-III |
| 'partial pressure of blood carbon dioxide (Pco2)' | 8 | GECCO \| CAPNETZ \| MC-19 \| Frankfurt \| Erlangen \| LEOSS \| Aachen \| MIMIC-III |
| 'body weight' | 7 | GECCO \| CAPNETZ \| MC-19 \| Erlangen \| PaCovid \| Aachen \| Brazilian Variables |
| cough | 7 | GECCO \| CAPNETZ \| MC-19 \| LEOSS \| PaCovid \| Brazilian Variables \| MIMIC-III |
| 'D-dimer (human)' | 7 | GECCO \| MC-19 \| Frankfurt \| LEOSS \| PaCovid \| Aachen \| MIMIC-III |
| 'Heart Rate' | 7 | GECCO \| CAPNETZ \| MC-19 \| Erlangen \| PaCovid \| Aachen \| Brazilian Variables |
| hematocrit | 7 | CAPNETZ \| MC-19 \| Erlangen \| LEOSS \| PaCovid \| Aachen \| MIMIC-III |
| neutrophil | 7 | GECCO \| CAPNETZ \| MC-19 \| Erlangen \| LEOSS \| PaCovid \| MIMIC-III |
| Other | 7 | GECCO \| CAPNETZ \| LEOSS \| PaCovid \| Brazilian Variables \| MIMIC-III \| IBM Explorys |
| Procalcitonin | 7 | GECCO \| CAPNETZ \| MC-19 \| Erlangen \| LEOSS \| PaCovid \| Aachen |
| 'systolic blood pressure' | 7 | GECCO \| CAPNETZ \| |

| | | |
|---|---|---|
| | | MC-19 \| LEOSS \| PaCovid \| Aachen \| Brazilian Variables |
| age | 6 | CAPNETZ \| MC-19 \| LEOSS \| PaCovid \| Aachen \| Brazilian Variables |
| Albumin | 6 | CAPNETZ \| MC-19 \| Erlangen \| PaCovid \| Aachen \| MIMIC-III |
| 'computed tomography' | 6 | GECCO \| CAPNETZ \| MC-19 \| LEOSS \| PaCovid \| Brazilian Variables |
| 'Creatine Kinase' | 6 | MC-19 \| Erlangen \| LEOSS \| PaCovid \| Aachen \| MIMIC-III |
| Dialysis | 6 | CAPNETZ \| Erlangen \| LEOSS \| PaCovid \| Brazilian Variables \| MIMIC-III |
| dyspnea | 6 | GECCO \| CAPNETZ \| MC-19 \| LEOSS \| PaCovid \| Brazilian Variables |
| Ferritin | 6 | GECCO \| MC-19 \| Frankfurt \| LEOSS \| PaCovid \| MIMIC-III |
| fever | 6 | GECCO \| CAPNETZ \| MC-19 \| LEOSS \| Brazilian Variables \| MIMIC-III |
| 'fibrinogen complex' | 6 | GECCO \| MC-19 \| Frankfurt \| Erlangen \| LEOSS \| PaCovid |
| 'Gamma-Glutamyl Transpeptidase' | 6 | GECCO \| MC-19 \| Erlangen \| LEOSS \| PaCovid \| MIMIC-III |
| glucose | 6 | CAPNETZ \| MC-19 \| Erlangen \| LEOSS \| PaCovid \| MIMIC-III |
| Interleukin-6 | 6 | GECCO \| MC-19 \| Frankfurt \| LEOSS \| PaCovid \| Aachen |
| No | 6 | GECCO \| CAPNETZ \| MC-19 \| LEOSS \| PaCovid \| |

| | | |
|---|---|---|
| | | IBM Explorys |
| 'Peripheral Oxygen Saturation' | 6 | GECCO \| Erlangen \| PaCovid \| Aachen \| Brazilian Variables \| MIMIC-III |
| 'Positive end expiratory pressure' | 6 | Frankfurt \| Erlangen \| LEOSS \| PaCovid \| Aachen \| MIMIC-III |
| tracheostomy | 6 | GECCO \| CAPNETZ \| LEOSS \| PaCovid \| Brazilian Variables \| MIMIC-III |
| Yes | 6 | GECCO \| CAPNETZ \| MC-19 \| LEOSS \| PaCovid \| IBM Explorys |
| 'activated partial thromboplastin time' | 5 | GECCO \| Erlangen \| LEOSS \| PaCovid \| MIMIC-III |
| 'Admission date' | 5 | CAPNETZ \| Erlangen \| PaCovid \| Brazilian Variables \| IBM Explorys |
| Asthma | 5 | GECCO \| CAPNETZ \| LEOSS \| PaCovid \| Brazilian Variables |
| azithromycin | 5 | CAPNETZ \| MC-19 \| LEOSS \| PaCovid \| MIMIC-III |
| 'base excess' | 5 | CAPNETZ \| MC-19 \| PaCovid \| Aachen \| MIMIC-III |
| 'body height' | 5 | GECCO \| CAPNETZ \| MC-19 \| PaCovid \| Brazilian Variables |
| 'body mass index' | 5 | CAPNETZ \| MC-19 \| LEOSS \| Aachen \| Brazilian Variables |
| 'cerebrovascular disease' | 5 | GECCO \| CAPNETZ \| LEOSS \| PaCovid \| Brazilian Variables |
| 'chronic obstructive pulmonary disease' | 5 | GECCO \| CAPNETZ \| LEOSS \| PaCovid \| Brazilian Variables |

| | | |
|---|---|---|
| Dead | 5 | GECCO \| CAPNETZ \| PaCovid \| Aachen \| IBM Explorys |
| diarrhea | 5 | GECCO \| MC-19 \| LEOSS \| PaCovid \| Brazilian Variables |
| 'ethnic group' | 5 | GECCO \| LEOSS \| PaCovid \| Brazilian Variables \| IBM Explorys |
| Feces | 5 | Frankfurt \| Erlangen \| LEOSS \| PaCovid \| MIMIC-III |
| 'High-Flow Nasal Cannula Oxygen Therapy' | 5 | GECCO \| MC-19 \| Erlangen \| LEOSS \| PaCovid |
| Hypertension | 5 | GECCO \| CAPNETZ \| MC-19 \| LEOSS \| PaCovid |
| 'Intensive Care Unit Admission Status' | 5 | GECCO \| CAPNETZ \| Frankfurt \| PaCovid \| Brazilian Variables |
| 'International Normalized Ratio' | 5 | GECCO \| CAPNETZ \| LEOSS \| PaCovid \| MIMIC-III |
| 'invasive mechanical ventilation' | 5 | CAPNETZ \| LEOSS \| PaCovid \| Brazilian Variables \| MIMIC-III |
| lactate | 5 | GECCO \| CAPNETZ \| Erlangen \| PaCovid \| MIMIC-III |
| 'Non-Invasive Mechanical Ventilation' | 5 | GECCO \| CAPNETZ \| LEOSS \| PaCovid \| MIMIC-III |
| 'N-Terminal Fragment Brain Natriuretic Protein' | 5 | GECCO \| CAPNETZ \| MC-19 \| PaCovid \| MIMIC-III |
| 'oxygen saturation' | 5 | CAPNETZ \| MC-19 \| Erlangen \| LEOSS \| MIMIC-III |
| 'Partial Pressure Arterial Oxygen to Fraction Inspired | 5 | MC-19 \| Frankfurt \| Erlangen \| Aachen \| |

| | | |
|---|---|---|
| Oxygen Ratio Measurement' | | Brazilian Variables |
| 'pH value' | 5 | GECCO \| CAPNETZ \| MC-19 \| Erlangen \| MIMIC-III |
| piperacillin-tazobactam | 5 | CAPNETZ \| MC-19 \| Erlangen \| PaCovid \| MIMIC-III |
| 'pleural effusion' | 5 | CAPNETZ \| LEOSS \| PaCovid \| Brazilian Variables \| MIMIC-III |
| 'Pulmonary embolism' | 5 | GECCO \| CAPNETZ \| LEOSS \| PaCovid \| Brazilian Variables |
| 'rheumatoid arthritis' | 5 | GECCO \| CAPNETZ \| LEOSS \| PaCovid \| Brazilian Variables |
| 'Sequential organ failure assessment score' | 5 | GECCO \| Frankfurt \| LEOSS \| PaCovid \| MIMIC-III |
| sodium | 5 | CAPNETZ \| MC-19 \| Erlangen \| PaCovid \| MIMIC-III |
| 'start date' | 5 | CAPNETZ \| LEOSS \| PaCovid \| Brazilian Variables \| IBM Explorys |
| 'Tidal Volume' | 5 | Frankfurt \| Erlangen \| PaCovid \| Aachen \| MIMIC-III |
| 'total bilirubin measurement' | 5 | CAPNETZ \| Erlangen \| PaCovid \| Aachen \| MIMIC-III |
| triglyceride | 5 | MC-19 \| Frankfurt \| LEOSS \| PaCovid \| MIMIC-III |
| Urea | 5 | CAPNETZ \| Erlangen \| LEOSS \| PaCovid \| Aachen |
| urine | 5 | CAPNETZ \| Erlangen \| LEOSS \| PaCovid \| MIMIC-III |
| adrenaline | 4 | LEOSS \| PaCovid \| Aachen \| |

| | | |
|---|---|---|
| | | MIMIC-III |
| 'Alkaline Phosphatase' | 4 | MC-19 \| Erlangen \| PaCovid \| MIMIC-III |
| ampicillin-sulbactam | 4 | CAPNETZ \| Erlangen \| PaCovid \| MIMIC-III |
| antibiotic | 4 | CAPNETZ \| Erlangen \| PaCovid \| Brazilian Variables |
| bilirubin | 4 | GECCO \| LEOSS \| Aachen \| MIMIC-III |
| blood | 4 | CAPNETZ \| Erlangen \| LEOSS \| MIMIC-III |
| 'blood hemoglobin A1c level' | 4 | CAPNETZ \| LEOSS \| PaCovid \| MIMIC-III |
| cancer | 4 | GECCO \| MC-19 \| LEOSS \| Brazilian Variables |
| 'CD4-positive T-lymphocyte count' | 4 | CAPNETZ \| LEOSS \| PaCovid \| MIMIC-III |
| ceftazidime | 4 | CAPNETZ \| Erlangen \| PaCovid \| MIMIC-III |
| ceftriaxone | 4 | MC-19 \| Erlangen \| PaCovid \| MIMIC-III |
| 'Chest pain' | 4 | GECCO \| CAPNETZ \| PaCovid \| MIMIC-III |
| Cirrhosis | 4 | GECCO \| CAPNETZ \| LEOSS \| PaCovid |
| 'coronary artery disease' | 4 | GECCO \| CAPNETZ \| LEOSS \| Brazilian Variables |
| 'Creatine Kinase MB' | 4 | LEOSS \| PaCovid \| Aachen \| MIMIC-III |
| 'cyclosporin A' | 4 | GECCO \| Frankfurt \| LEOSS \| MIMIC-III |
| 'cystic fibrosis' | 4 | GECCO \| LEOSS \| PaCovid \| Brazilian Variables |
| delirium | 4 | CAPNETZ \| Erlangen \| |

| | | |
|---|---|---|
| | | LEOSS \| Brazilian Variables |
| dementia | 4 | GECCO \| CAPNETZ \| LEOSS \| PaCovid |
| Diabetes | 4 | GECCO \| MC-19 \| LEOSS \| MIMIC-III |
| 'Discharge date' | 4 | CAPNETZ \| PaCovid \| Brazilian Variables \| IBM Explorys |
| eosinophil | 4 | CAPNETZ \| MC-19 \| Erlangen \| MIMIC-III |
| erythrocyte | 4 | CAPNETZ \| MC-19 \| Erlangen \| MIMIC-III |
| 'Extracorporeal Membrane Oxygenation' | 4 | GECCO \| LEOSS \| PaCovid \| MIMIC-III |
| female | 4 | GECCO \| MC-19 \| PaCovid \| IBM Explorys |
| 'Have Other Symptoms' | 4 | GECCO \| MC-19 \| LEOSS \| PaCovid |
| Headache | 4 | GECCO \| LEOSS \| PaCovid \| MIMIC-III |
| 'heart failure' | 4 | GECCO \| LEOSS \| PaCovid \| Brazilian Variables |
| Hospitalization | 4 | GECCO \| CAPNETZ \| LEOSS \| IBM Explorys |
| 'human immunodeficiency virus infectious disease' | 4 | GECCO \| CAPNETZ \| LEOSS \| Brazilian Variables |
| hydrogencarbonate | 4 | CAPNETZ \| MC-19 \| Erlangen \| MIMIC-III |
| magnesium | 4 | MC-19 \| Erlangen \| PaCovid \| MIMIC-III |
| male | 4 | GECCO \| MC-19 \| PaCovid \| IBM Explorys |
| 'Mean inspiratory airway pressure' | 4 | Frankfurt \| Erlangen \| PaCovid \| MIMIC-III |
| 'Mechanical Ventilation' | 4 | GECCO \| CAPNETZ \| |

| | | |
|---|---|---|
| | | PaCovid \| MIMIC-III |
| Medication | 4 | GECCO \| CAPNETZ \| Erlangen \| PaCovid |
| Migraine | 4 | GECCO \| LEOSS \| PaCovid \| Brazilian Variables |
| monocyte | 4 | CAPNETZ \| MC-19 \| Erlangen \| MIMIC-III |
| noradrenaline | 4 | Erlangen \| PaCovid \| Aachen \| MIMIC-III |
| 'oral anticoagulant' | 4 | GECCO \| CAPNETZ \| LEOSS \| PaCovid |
| 'Patient Discharge' | 4 | CAPNETZ \| LEOSS \| PaCovid \| IBM Explorys |
| potassium | 4 | MC-19 \| Erlangen \| PaCovid \| MIMIC-III |
| prednisolone | 4 | GECCO \| LEOSS \| PaCovid \| MIMIC-III |
| 'Prone Position' | 4 | GECCO \| LEOSS \| PaCovid \| Brazilian Variables |
| 'pulmonary fibrosis' | 4 | GECCO \| CAPNETZ \| LEOSS \| PaCovid |
| seizure | 4 | GECCO \| LEOSS \| PaCovid \| MIMIC-III |
| 'Smoking Status' | 4 | GECCO \| MC-19 \| LEOSS \| PaCovid |
| Specify | 4 | CAPNETZ \| LEOSS \| PaCovid \| Brazilian Variables |
| Unknown | 4 | GECCO \| LEOSS \| PaCovid \| IBM Explorys |
| vancomycin | 4 | CAPNETZ \| Erlangen \| PaCovid \| MIMIC-III |
| 'vitamin D' | 4 | MC-19 \| Frankfurt \| LEOSS \| MIMIC-III |
| Vomiting | 4 | GECCO \| PaCovid \| |

| | | |
|---|---|---|
| | | Brazilian Variables \| MIMIC-III |
| 'X-Ray Imaging' | 4 | GECCO \| CAPNETZ \| MC-19 \| PaCovid |
| 3MRGN | 3 | CAPNETZ \| LEOSS \| PaCovid |
| 4MRGN | 3 | CAPNETZ \| LEOSS \| PaCovid |
| 'acetylsalicylic acid' | 3 | GECCO \| LEOSS \| PaCovid |
| 'Acute respiratory distress syndrome' | 3 | GECCO \| Erlangen \| Brazilian Variables |
| 'Airway Plateau Pressure' | 3 | Frankfurt \| PaCovid \| MIMIC-III |
| Ampicillin | 3 | CAPNETZ \| Erlangen \| MIMIC-III |
| 'Angiotensin-Converting Enzyme Inhibitors' | 3 | GECCO \| LEOSS \| PaCovid |
| Antithrombin-III | 3 | GECCO \| PaCovid \| MIMIC-III |
| 'Arterial Oxygen Saturation' | 3 | GECCO \| Aachen \| MIMIC-III |
| Arthralgia | 3 | GECCO \| PaCovid \| Brazilian Variables |
| 'autoimmune disease' | 3 | CAPNETZ \| PaCovid \| Brazilian Variables |
| azathioprine | 3 | Frankfurt \| LEOSS \| PaCovid |
| basophil | 3 | MC-19 \| Erlangen \| MIMIC-III |
| 'Biospecimen Type' | 3 | Erlangen \| Brazilian Variables \| MIMIC-III |
| 'Blood Culture' | 3 | CAPNETZ \| Erlangen \| PaCovid |
| 'blood serum' | 3 | CAPNETZ \| Frankfurt \| LEOSS |

| | | |
|---|---|---|
| 'brain cancer' | 3 | CAPNETZ \| LEOSS \| PaCovid |
| 'breast cancer' | 3 | CAPNETZ \| LEOSS \| PaCovid |
| 'Bronchoalveolar Lavage Fluid' | 3 | CAPNETZ \| LEOSS \| PaCovid |
| Calcifediol | 3 | GECCO \| LEOSS \| MIMIC-III |
| calcium | 3 | MC-19 \| Erlangen \| MIMIC-III |
| 'Calcium Cation' | 3 | Erlangen \| PaCovid \| MIMIC-III |
| 'carotid stenosis' | 3 | GECCO \| CAPNETZ \| LEOSS |
| 'CD8-positive T-lymphocyte count' | 3 | CAPNETZ \| LEOSS \| PaCovid |
| chloride | 3 | MC-19 \| Erlangen \| MIMIC-III |
| ciprofloxacin | 3 | CAPNETZ \| Erlangen \| MIMIC-III |
| conjunctivitis | 3 | GECCO \| PaCovid \| Brazilian Variables |
| 'continuous positive airway pressure' | 3 | CAPNETZ \| LEOSS \| PaCovid |
| corticosteroid | 3 | GECCO \| LEOSS \| PaCovid |
| cortisol | 3 | LEOSS \| PaCovid \| MIMIC-III |
| daptomycin | 3 | Erlangen \| PaCovid \| MIMIC-III |
| darunavir | 3 | GECCO \| MC-19 \| LEOSS |
| 'date of birth' | 3 | GECCO \| Erlangen \| Brazilian Variables |
| 'date of test' | 3 | CAPNETZ \| PaCovid \| Brazilian Variables |
| 'Date transplant' | 3 | CAPNETZ \| LEOSS \| |

| | | |
|---|---|---|
| | | Brazilian Variables |
| day | 3 | CAPNETZ \| MC-19 \| Frankfurt |
| 'Delivered oxygen flow rate' | 3 | CAPNETZ \| PaCovid \| MIMIC-III |
| 'diabetes mellitus' | 3 | GECCO \| CAPNETZ \| Brazilian Variables |
| dioxygen | 3 | LEOSS \| PaCovid \| MIMIC-III |
| 'Discharged Alive' | 3 | GECCO \| MC-19 \| PaCovid |
| 'Disease Severity' | 3 | GECCO \| LEOSS \| IBM Explorys |
| disorientation | 3 | GECCO \| LEOSS \| PaCovid |
| dobutamine | 3 | PaCovid \| Aachen \| MIMIC-III |
| 'driving pressure' | 3 | Frankfurt \| Aachen \| MIMIC-III |
| drug | 3 | GECCO \| CAPNETZ \| LEOSS |
| Dysphagia | 3 | CAPNETZ \| LEOSS \| PaCovid |
| encephalitis | 3 | GECCO \| LEOSS \| PaCovid |
| epilepsy | 3 | GECCO \| LEOSS \| PaCovid |
| erythromycin | 3 | CAPNETZ \| Erlangen \| MIMIC-III |
| 'Former Smoker' | 3 | GECCO \| CAPNETZ \| PaCovid |
| ganciclovir | 3 | GECCO \| LEOSS \| MIMIC-III |
| gentamicin | 3 | CAPNETZ \| Erlangen \| MIMIC-III |
| glucocorticoid | 3 | GECCO \| Frankfurt \| LEOSS |
| Glucose:MCnc:Pt:CSF:Qn | 3 | LEOSS \| PaCovid \| MIMIC-III |

| | | |
|---|---|---|
| 'gram per deciliter' | 3 | CAPNETZ \| MC-19 \| Brazilian Variables |
| Heart | 3 | GECCO \| CAPNETZ \| LEOSS |
| Heparin | 3 | GECCO \| Erlangen \| MIMIC-III |
| 'high-density lipoprotein cholesterol' | 3 | MC-19 \| PaCovid \| MIMIC-III |
| hydroxychloroquine | 3 | GECCO \| MC-19 \| LEOSS |
| Identifier | 3 | GECCO \| CAPNETZ \| MC-19 |
| 'Immunoglobulin A' | 3 | CAPNETZ \| LEOSS \| MIMIC-III |
| 'Immunoglobulin G' | 3 | CAPNETZ \| LEOSS \| MIMIC-III |
| 'Inspiratory/expiratory ratio' | 3 | Erlangen \| Aachen \| MIMIC-III |
| Insulin | 3 | Erlangen \| LEOSS \| MIMIC-III |
| 'intensive care' | 3 | GECCO \| CAPNETZ \| IBM Explorys |
| 'Intubation Procedure' | 3 | LEOSS \| PaCovid \| MIMIC-III |
| Kidney | 3 | GECCO \| CAPNETZ \| LEOSS |
| Lactate:MCnc:Pt:CSF:Qn | 3 | GECCO \| LEOSS \| PaCovid |
| Linezolid | 3 | CAPNETZ \| Erlangen \| MIMIC-III |
| 'Long-term oxygen therapy' | 3 | CAPNETZ \| LEOSS \| PaCovid |
| Lopinavir/Ritonavir | 3 | GECCO \| MC-19 \| LEOSS |
| 'loss of appetite' | 3 | GECCO \| PaCovid \| Brazilian Variables |
| 'low-density lipoprotein cholesterol' | 3 | CAPNETZ \| LEOSS \| PaCovid |

| | | |
|---|---|---|
| Lung | 3 | GECCO \| CAPNETZ \| LEOSS |
| 'lung cancer' | 3 | CAPNETZ \| LEOSS \| PaCovid |
| 'mean arterial pressure' | 3 | PaCovid \| Aachen \| MIMIC-III |
| 'mean corpuscular hemoglobin' | 3 | MC-19 \| Erlangen \| MIMIC-III |
| 'mean corpuscular hemoglobin concentration' | 3 | MC-19 \| Erlangen \| MIMIC-III |
| 'mean corpuscular volume' | 3 | MC-19 \| Erlangen \| MIMIC-III |
| meropenem | 3 | Erlangen \| PaCovid \| MIMIC-III |
| 'Methicillin Resistant Staphylococcus Aureus' | 3 | CAPNETZ \| LEOSS \| PaCovid |
| methotrexate | 3 | LEOSS \| PaCovid \| MIMIC-III |
| 'Microbiology Specimen Type' | 3 | CAPNETZ \| PaCovid \| IBM Explorys |
| 'Mild to moderate' | 3 | GECCO \| LEOSS \| IBM Explorys |
| Moderate | 3 | GECCO \| PaCovid \| IBM Explorys |
| moxifloxacin | 3 | CAPNETZ \| Erlangen \| MIMIC-III |
| 'multiple sclerosis' | 3 | GECCO \| LEOSS \| PaCovid |
| 'nasal congestion' | 3 | GECCO \| PaCovid \| MIMIC-III |
| Nausea | 3 | GECCO \| PaCovid \| MIMIC-III |
| negative | 3 | CAPNETZ \| PaCovid \| IBM Explorys |
| 'Never Smoker' | 3 | GECCO \| CAPNETZ \| PaCovid |

| | | |
|---|---|---|
| normal | 3 | GECCO \| MC-19 \| IBM Explorys |
| 'Not Applicable' | 3 | GECCO \| LEOSS \| PaCovid |
| 'obstructive sleep apnea' | 3 | GECCO \| LEOSS \| PaCovid |
| 'organ transplantation' | 3 | CAPNETZ \| LEOSS \| Brazilian Variables |
| 'Osmolality Measurement' | 3 | Erlangen \| PaCovid \| MIMIC-III |
| paracetamol | 3 | GECCO \| PaCovid \| MIMIC-III |
| Pathogen | 3 | CAPNETZ \| MC-19 \| Brazilian Variables |
| 'Peak Inspiratory Pressure' | 3 | Erlangen \| Aachen \| MIMIC-III |
| 'Peak pressure' | 3 | Frankfurt \| Erlangen \| PaCovid |
| penicillin | 3 | CAPNETZ \| LEOSS \| MIMIC-III |
| percent | 3 | CAPNETZ \| MC-19 \| Brazilian Variables |
| 'Peripheral Arterial Occlusive Disease' | 3 | GECCO \| CAPNETZ \| PaCovid |
| pH:LsCnc:Pt:BldA:Qn | 3 | GECCO \| Aachen \| MIMIC-III |
| pharyngitis | 3 | LEOSS \| PaCovid \| Brazilian Variables |
| piperacillin | 3 | CAPNETZ \| LEOSS \| MIMIC-III |
| pneumonia | 3 | GECCO \| CAPNETZ \| MC-19 |
| polyneuropathy | 3 | GECCO \| LEOSS \| PaCovid |
| positive | 3 | CAPNETZ \| PaCovid \| IBM Explorys |
| 'prostate cancer' | 3 | CAPNETZ \| LEOSS \| PaCovid |

| | | |
|---|---|---|
| 'protein measurement' | 3 | CAPNETZ \| Erlangen \| MIMIC-III |
| 'prothrombin time' | 3 | Erlangen \| PaCovid \| MIMIC-III |
| 'pulmonary hypertension' | 3 | GECCO \| LEOSS \| PaCovid |
| 'red blood cell distribution width' | 3 | MC-19 \| Erlangen \| MIMIC-III |
| Rhinorrhea | 3 | LEOSS \| PaCovid \| Brazilian Variables |
| 'Route of Administration' | 3 | CAPNETZ \| PaCovid \| IBM Explorys |
| Sarilumab | 3 | GECCO \| MC-19 \| LEOSS |
| 'serum albumin level' | 3 | GECCO \| LEOSS \| MIMIC-III |
| sirolimus | 3 | GECCO \| LEOSS \| MIMIC-III |
| 'Sleep apnea' | 3 | GECCO \| CAPNETZ \| PaCovid |
| sputum | 3 | CAPNETZ \| LEOSS \| PaCovid |
| 'start time' | 3 | CAPNETZ \| PaCovid \| Aachen |
| stroke | 3 | GECCO \| CAPNETZ \| PaCovid |
| 'systemic lupus erythematosus' | 3 | CAPNETZ \| LEOSS \| Brazilian Variables |
| Tacrolimus | 3 | GECCO \| LEOSS \| MIMIC-III |
| 'thyroid stimulating hormone' | 3 | LEOSS \| PaCovid \| MIMIC-III |
| tobramycin | 3 | CAPNETZ \| Erlangen \| MIMIC-III |
| 'Total Protein Measurement' | 3 | MC-19 \| Erlangen \| MIMIC-III |
| Transferrin | 3 | Frankfurt \| PaCovid \| |

| | | |
|---|---|---|
| | | MIMIC-III |
| 'transient cerebral ischemia' | 3 | CAPNETZ \| LEOSS \| PaCovid |
| 'Treatment duration' | 3 | Erlangen \| LEOSS \| Brazilian Variables |
| 'treatment start date' | 3 | CAPNETZ \| Brazilian Variables \| IBM Explorys |
| 'Troponin T' | 3 | CAPNETZ \| LEOSS \| MIMIC-III |
| 'uric acid' | 3 | MC-19 \| Erlangen \| MIMIC-III |
| 'Vancomycin Resistant Enterococcus' | 3 | CAPNETZ \| LEOSS \| PaCovid |
| Wheezing | 3 | GECCO \| LEOSS \| PaCovid |
| 6alpha-methylprednisolone | 2 | GECCO \| MIMIC-III |
| Abciximab | 2 | GECCO \| MIMIC-III |
| 'Abdominal pain' | 2 | GECCO \| PaCovid |
| 'Abnormal reflex' | 2 | LEOSS \| PaCovid |
| 'Acute coronary syndrome' | 2 | CAPNETZ \| LEOSS |
| 'Acute Myocardial Infarction' | 2 | LEOSS \| Brazilian Variables |
| Adalimumab | 2 | GECCO \| LEOSS |
| Adenoviridae | 2 | CAPNETZ \| PaCovid |
| ADP | 2 | LEOSS \| MIMIC-III |
| African | 2 | GECCO \| PaCovid |
| Ageusia | 2 | GECCO \| LEOSS |
| 'Airway pressure delta' | 2 | Aachen \| MIMIC-III |
| aldosterone | 2 | GECCO \| PaCovid |
| amikacin | 2 | CAPNETZ \| MIMIC-III |
| Anakinra | 2 | GECCO \| LEOSS |

| | | |
|---|---|---|
| 'Angiotensin II Type 1 Receptor Blockers' | 2 | LEOSS \| PaCovid |
| 'anion gap' | 2 | Erlangen \| MIMIC-III |
| Anosmia | 2 | GECCO \| LEOSS |
| anticoagulant | 2 | LEOSS \| Brazilian Variables |
| 'Anticoagulation Therapy' | 2 | GECCO \| PaCovid |
| anticonvulsant | 2 | LEOSS \| PaCovid |
| 'antifungal agent' | 2 | CAPNETZ \| PaCovid |
| 'antigen of Legionella' | 2 | CAPNETZ \| PaCovid |
| 'antimicrobial resistance test platform' | 2 | CAPNETZ \| IBM Explorys |
| Antipyretics | 2 | GECCO \| Brazilian Variables |
| 'Antithrombin Activity Measurement' | 2 | Erlangen \| LEOSS |
| anxiety | 2 | PaCovid \| MIMIC-III |
| 'aortic valve stenosis' | 2 | LEOSS \| PaCovid |
| aphasia | 2 | LEOSS \| PaCovid |
| apixaban | 2 | GECCO \| LEOSS |
| apraxia | 2 | LEOSS \| PaCovid |
| Arab | 2 | GECCO \| PaCovid |
| argatroban | 2 | LEOSS \| MIMIC-III |
| Arrhythmia | 2 | GECCO \| LEOSS |
| 'Assisted spontaneous breathing' | 2 | LEOSS \| PaCovid |
| asthenia | 2 | GECCO \| Brazilian Variables |
| Asymptomatic | 2 | GECCO \| LEOSS |
| Ataxia | 2 | LEOSS \| PaCovid |
| atazanavir | 2 | GECCO \| LEOSS |

| | | |
|---|---|---|
| 'atrial fibrillation' | 2 | CAPNETZ \| LEOSS |
| 'autoimmune hepatitis' | 2 | LEOSS \| PaCovid |
| Autopsy | 2 | CAPNETZ \| LEOSS |
| 'Axillary Temperature' | 2 | CAPNETZ \| Brazilian Variables |
| 'Barthel Index of Activities of Daily Living' | 2 | LEOSS \| PaCovid |
| Basiliximab | 2 | GECCO \| LEOSS |
| 'Begin Ventilation' | 2 | Frankfurt \| Erlangen |
| 'Behavioral Pain Scale' | 2 | Erlangen \| PaCovid |
| 'beta-adrenergic antagonist' | 2 | LEOSS \| PaCovid |
| Better | 2 | CAPNETZ \| MC-19 |
| 'Billion per Liter' | 2 | CAPNETZ \| MC-19 |
| 'Birth Year' | 2 | CAPNETZ \| IBM Explorys |
| bivalirudin | 2 | GECCO \| MIMIC-III |
| bleeding | 2 | GECCO \| PaCovid |
| 'blood flow rate' | 2 | Erlangen \| PaCovid |
| 'Blood Gas Analysis' | 2 | CAPNETZ \| Erlangen |
| 'blood plasma' | 2 | Frankfurt \| MIMIC-III |
| 'Blood Urea Nitrogen' | 2 | CAPNETZ \| MC-19 |
| B-lymphocyte | 2 | CAPNETZ \| LEOSS |
| 'Body fluid' | 2 | Erlangen \| MIMIC-III |
| 'Brain natriuretic peptide' | 2 | CAPNETZ \| LEOSS |
| bronchitis | 2 | LEOSS \| Brazilian Variables |
| 'calcium channel blocker' | 2 | LEOSS \| PaCovid |
| 'calcium gluconate' | 2 | Erlangen \| MIMIC-III |
| Canakinumab | 2 | GECCO \| LEOSS |
| 'Carbon dioxide measurement, partial | 2 | GECCO \| Aachen |

| | | |
|---|---|---|
| pressure' | | |
| 'Carbon dioxide:PPres:Pt:BldC:Qn' | 2 | GECCO \| MIMIC-III |
| 'cardiac arrhythmia' | 2 | GECCO \| PaCovid |
| 'cardiovascular system disease' | 2 | GECCO \| Brazilian Variables |
| 'Cause of Death' | 2 | CAPNETZ \| LEOSS |
| 'CD4 T Cell to Lymphocyte Ratio Measurement' | 2 | CAPNETZ \| LEOSS |
| 'CD8 T Cell to Lymphocyte Ratio Measurement' | 2 | CAPNETZ \| LEOSS |
| cefazolin | 2 | Erlangen \| MIMIC-III |
| cefuroxime | 2 | CAPNETZ \| Erlangen |
| 'Center for Disease Control and Prevention Classification System for HIV-Infected Adults and Adolescents Clinical Classification' | 2 | CAPNETZ \| LEOSS |
| 'Central Venous Access Catheter' | 2 | Erlangen \| Brazilian Variables |
| 'Central venous sinus thrombosis' | 2 | LEOSS \| PaCovid |
| 'Cerebral hemorrhage' | 2 | LEOSS \| PaCovid |
| 'cerebrospinal fluid' | 2 | LEOSS \| PaCovid |
| Chills | 2 | GECCO \| Brazilian Variables |
| cholesterol | 2 | MC-19 \| PaCovid |
| 'Cholesterol total' | 2 | CAPNETZ \| MIMIC-III |
| 'chronic kidney disease' | 2 | GECCO \| LEOSS |
| 'Chronic Liver Disease' | 2 | GECCO \| LEOSS |
| 'Chronic Lung Disorder' | 2 | GECCO \| LEOSS |
| 'Chronic neurological | 2 | GECCO \| CAPNETZ |

| | | |
|---|---|---|
| disease' | | |
| 'Churg-Strauss syndrome' | 2 | CAPNETZ \| LEOSS |
| cladribine | 2 | LEOSS \| MIMIC-III |
| clarithromycin | 2 | CAPNETZ \| PaCovid |
| clindamycin | 2 | Erlangen \| MIMIC-III |
| 'clinical visit date' | 2 | CAPNETZ \| Frankfurt |
| clopidogrel | 2 | GECCO \| LEOSS |
| colchicine | 2 | GECCO \| LEOSS |
| 'Collection Date Time' | 2 | CAPNETZ \| PaCovid |
| 'colon cancer' | 2 | CAPNETZ \| PaCovid |
| comment | 2 | LEOSS \| PaCovid |
| comorbidity | 2 | CAPNETZ \| MC-19 |
| 'Complete Blood Count' | 2 | CAPNETZ \| Erlangen |
| completed | 2 | LEOSS \| IBM Explorys |
| 'Complicated phase' | 2 | GECCO \| LEOSS |
| Complication | 2 | GECCO \| CAPNETZ |
| 'confusion assessment method for the intensive care unit' | 2 | Erlangen \| PaCovid |
| consciousness | 2 | Erlangen \| PaCovid |
| 'Convalescent Plasma' | 2 | GECCO \| LEOSS |
| co-trimoxazole | 2 | CAPNETZ \| Erlangen |
| 'Critical phase' | 2 | GECCO \| LEOSS |
| 'CSF oligoclonal band' | 2 | LEOSS \| PaCovid |
| 'Cumulative Dose' | 2 | CAPNETZ \| Erlangen |
| 'Current Smoker' | 2 | CAPNETZ \| PaCovid |
| 'Daily Dose' | 2 | CAPNETZ \| PaCovid |
| Dalteparin | 2 | GECCO \| MIMIC-III |

| | | |
|---|---|---|
| 'date and time of blood collection' | 2 | CAPNETZ \| PaCovid |
| 'Date of Death' | 2 | CAPNETZ \| Brazilian Variables |
| 'date of diagnosis' | 2 | LEOSS \| IBM Explorys |
| 'Date of procedure' | 2 | Erlangen \| IBM Explorys |
| 'date of specimen collection' | 2 | CAPNETZ \| IBM Explorys |
| death | 2 | GECCO \| PaCovid |
| 'Deep Vein Thrombosis' | 2 | PaCovid \| Brazilian Variables |
| Demographics | 2 | GECCO \| IBM Explorys |
| depression | 2 | GECCO \| PaCovid |
| Dermatology | 2 | CAPNETZ \| IBM Explorys |
| dermatomyositis | 2 | CAPNETZ \| LEOSS |
| diagnosis | 2 | Erlangen \| IBM Explorys |
| 'Diagnosis Code' | 2 | Erlangen \| IBM Explorys |
| 'Dialysis Fluid' | 2 | Erlangen \| MIMIC-III |
| 'Dialysis or hemofiltration' | 2 | GECCO \| PaCovid |
| 'dipeptidyl peptidase-4 inhibitor' | 2 | LEOSS \| PaCovid |
| 'Direct Bilirubin Measurement' | 2 | Erlangen \| MIMIC-III |
| 'Discharge from intensive care service' | 2 | CAPNETZ \| Frankfurt |
| 'disease stage' | 2 | GECCO \| LEOSS |
| diuretic | 2 | LEOSS \| PaCovid |
| Documentation | 2 | CAPNETZ \| IBM Explorys |
| dose | 2 | Brazilian Variables \| IBM Explorys |
| 'dose unit' | 2 | CAPNETZ \| PaCovid |

| | | |
|---|---|---|
| doxycycline | 2 | CAPNETZ \| MC-19 |
| 'drug category' | 2 | CAPNETZ \| PaCovid |
| 'dry cough' | 2 | GECCO \| LEOSS |
| Dysarthria | 2 | LEOSS \| PaCovid |
| 'Ear, nose and throat medicine' | 2 | CAPNETZ \| IBM Explorys |
| edoxaban | 2 | GECCO \| LEOSS |
| 'EDTA-Treated Plasma Sample' | 2 | CAPNETZ \| Frankfurt |
| 'ejection fraction' | 2 | CAPNETZ \| LEOSS |
| electrocardiogram | 2 | Erlangen \| MIMIC-III |
| Electroencephalogram | 2 | LEOSS \| MIMIC-III |
| 'Electronic Cigarette' | 2 | CAPNETZ \| LEOSS |
| Electrophysiology | 2 | LEOSS \| IBM Explorys |
| 'emergency department' | 2 | Brazilian Variables \| IBM Explorys |
| emphysema | 2 | LEOSS \| Brazilian Variables |
| 'end date' | 2 | CAPNETZ \| PaCovid |
| 'End Ventilation' | 2 | Frankfurt \| Erlangen |
| 'end-tidal partial pressure of carbon dioxide (PETCO2)' | 2 | Erlangen \| PaCovid |
| 'Enoxaparin Sodium' | 2 | MC-19 \| MIMIC-III |
| 'Enteral Tube Feeding' | 2 | CAPNETZ \| Erlangen |
| Epidemiology | 2 | GECCO \| PaCovid |
| 'Epoetin Alfa' | 2 | Erlangen \| MIMIC-III |
| eptifibatide | 2 | GECCO \| MIMIC-III |
| 'Escherichia coli' | 2 | CAPNETZ \| PaCovid |
| etanercept | 2 | GECCO \| LEOSS |
| European | 2 | GECCO \| PaCovid |

| | | |
|---|---|---|
| everolimus | 2 | GECCO \| LEOSS |
| Famotidine | 2 | Erlangen \| MIMIC-III |
| Fatigue | 2 | GECCO \| LEOSS |
| favipiravir | 2 | GECCO \| LEOSS |
| 'Foreign Body' | 2 | Erlangen \| MIMIC-III |
| 'Frailty Index score' | 2 | GECCO \| PaCovid |
| 'Free Thyroxine Measurement' | 2 | PaCovid \| MIMIC-III |
| 'Glasgow coma scale' | 2 | LEOSS \| PaCovid |
| 'glomerular filtration rate' | 2 | CAPNETZ \| Erlangen |
| Glucose:MCnc:Pt:Urine:Qn | 2 | LEOSS \| MIMIC-III |
| Golimumab | 2 | GECCO \| LEOSS |
| 'Graft Versus Host Disease' | 2 | CAPNETZ \| LEOSS |
| 'gram per liter' | 2 | CAPNETZ \| MC-19 |
| 'granulomatosis with polyangiitis' | 2 | CAPNETZ \| LEOSS |
| 'Ground Glass Opacity' | 2 | MC-19 \| LEOSS |
| 'Group ID' | 2 | Erlangen \| IBM Explorys |
| 'Group name' | 2 | Erlangen \| IBM Explorys |
| Haloperidol | 2 | Erlangen \| MIMIC-III |
| Haptoglobin | 2 | PaCovid \| MIMIC-III |
| 'Health Care Provider Identifier' | 2 | Erlangen \| IBM Explorys |
| hemiplegia | 2 | CAPNETZ \| LEOSS |
| Hemoptysis | 2 | GECCO \| MIMIC-III |
| Hepatitis | 2 | CAPNETZ \| Erlangen |
| 'Hepatitis A Virus Antibody' | 2 | GECCO \| MIMIC-III |
| 'Hepatitis B Virus Antibody' | 2 | GECCO \| MIMIC-III |
| 'High Sensitivity Troponin T | 2 | GECCO \| PaCovid |

| | | |
|---|---|---|
| Assay' | | |
| 'high-density lipoprotein' | 2 | CAPNETZ \| LEOSS |
| 'Hispanic or Latin American' | 2 | GECCO \| PaCovid |
| 'HIV Entry Inhibitor' | 2 | CAPNETZ \| LEOSS |
| 'HIV Integrase Inhibitor' | 2 | CAPNETZ \| LEOSS |
| 'HIV positive' | 2 | CAPNETZ \| Frankfurt |
| 'HIV viral load' | 2 | CAPNETZ \| LEOSS |
| Home | 2 | CAPNETZ \| IBM Explorys |
| 'Hospital Departments' | 2 | CAPNETZ \| IBM Explorys |
| ibuprofen | 2 | LEOSS \| PaCovid |
| 'If a spinal tap was performed, please specify' | 2 | LEOSS \| PaCovid |
| 'If an EEG was performed, please specify' | 2 | LEOSS \| PaCovid |
| 'If electrophysiology was performed, please specify' | 2 | LEOSS \| PaCovid |
| iloprost | 2 | GECCO \| LEOSS |
| Imaging | 2 | GECCO \| PaCovid |
| 'Imaging Technique' | 2 | GECCO \| PaCovid |
| imipenem | 2 | CAPNETZ \| PaCovid |
| Immunoglobulin | 2 | GECCO \| LEOSS |
| 'Immunoglobulin M' | 2 | CAPNETZ \| MIMIC-III |
| 'immunosuppressive agent' | 2 | LEOSS \| Brazilian Variables |
| 'Indirect Bilirubin Measurement' | 2 | Erlangen \| MIMIC-III |
| 'Infectious diseases department' | 2 | Erlangen \| IBM Explorys |
| 'inflammatory bowel disease' | 2 | GECCO \| PaCovid |
| Infliximab | 2 | GECCO \| LEOSS |
| Influenza | 2 | GECCO \| LEOSS |

| | | |
|---|---|---|
| 'Influenza virus found' | 2 | CAPNETZ \| PaCovid |
| inhalation | 2 | CAPNETZ \| LEOSS |
| 'inpatient encounter' | 2 | CAPNETZ \| IBM Explorys |
| 'Intake of biological or other immunosuppresive drugs within the past 3 months' | 2 | CAPNETZ \| LEOSS |
| 'Intensive Care Unit' | 2 | CAPNETZ \| MIMIC-III |
| Interferon | 2 | GECCO \| LEOSS |
| 'Interferon Alpha' | 2 | GECCO \| LEOSS |
| 'Interferon Beta' | 2 | GECCO \| LEOSS |
| 'Interleukin-2 Receptor Subunit Alpha' | 2 | Frankfurt \| MIMIC-III |
| 'Internal Medicine' | 2 | CAPNETZ \| IBM Explorys |
| 'International Classification of Diseases, Tenth Revision' | 2 | Aachen \| IBM Explorys |
| 'Interventional Radiology' | 2 | MIMIC-III \| IBM Explorys |
| 'Intravenous Route of Administration' | 2 | CAPNETZ \| Erlangen |
| iron | 2 | Erlangen \| MIMIC-III |
| 'Ischemic stroke' | 2 | LEOSS \| PaCovid |
| Ivermectin | 2 | GECCO \| LEOSS |
| Ixekizumab | 2 | GECCO \| LEOSS |
| 'kidney disease' | 2 | CAPNETZ \| Brazilian Variables |
| Lactate:MCnc:Pt:BldA:Qn | 2 | GECCO \| Aachen |
| 'Large Platelet Count' | 2 | Erlangen \| MIMIC-III |
| 'Left Ventricular Ejection Fraction' | 2 | LEOSS \| PaCovid |
| Lepirudin | 2 | GECCO \| MIMIC-III |
| leukemia | 2 | CAPNETZ \| LEOSS |

| | | |
|---|---|---|
| 'leukocyte count' | 2 | Aachen \| MIMIC-III |
| 'Leukocytes in urine' | 2 | LEOSS \| MIMIC-III |
| 'Life threatening severity' | 2 | GECCO \| IBM Explorys |
| Lipase | 2 | Erlangen \| LEOSS |
| Liver | 2 | GECCO \| LEOSS |
| Location | 2 | PaCovid \| Brazilian Variables |
| 'location of death' | 2 | CAPNETZ \| LEOSS |
| 'lung disease' | 2 | CAPNETZ \| Brazilian Variables |
| Lymphadenopathy | 2 | GECCO \| PaCovid |
| lymphoma | 2 | CAPNETZ \| LEOSS |
| 'mean platelet volume' | 2 | MC-19 \| Erlangen |
| 'Measurement of protein in cerebrospinal fluid specimen' | 2 | LEOSS \| MIMIC-III |
| 'medical intervention' | 2 | PaCovid \| MIMIC-III |
| melanoma | 2 | LEOSS \| PaCovid |
| 'Memory impairment' | 2 | LEOSS \| PaCovid |
| 'Men who have Sex with Men' | 2 | CAPNETZ \| LEOSS |
| meningitis | 2 | GECCO \| LEOSS |
| meprednisone | 2 | GECCO \| MIMIC-III |
| Metastasis | 2 | CAPNETZ \| LEOSS |
| metformin | 2 | LEOSS \| PaCovid |
| metoprolol | 2 | Erlangen \| MIMIC-III |
| Microbiology | 2 | Erlangen \| IBM Explorys |
| 'Microbiology Specimen Test' | 2 | CAPNETZ \| PaCovid |
| 'Microscopic polyangiitis' | 2 | CAPNETZ \| LEOSS |

| | | |
|---|---|---|
| midazolam | 2 | Erlangen \| MIMIC-III |
| Mild | 2 | GECCO \| IBM Explorys |
| 'mild cognitive impairment' | 2 | GECCO \| LEOSS |
| 'Milk Protein-Based Energy Drink' | 2 | Erlangen \| MIMIC-III |
| 'Milliequivalent per Liter' | 2 | CAPNETZ \| MC-19 |
| 'millimetres of mercury' | 2 | CAPNETZ \| MC-19 |
| 'Millimole per Liter' | 2 | CAPNETZ \| MC-19 |
| 'Moderate to severe' | 2 | GECCO \| IBM Explorys |
| 'Modified Rankin Scale' | 2 | GECCO \| LEOSS |
| 'Montreal Cognitive Assessment Test score' | 2 | GECCO \| LEOSS |
| 'motor neuron disease' | 2 | LEOSS \| PaCovid |
| 'movement disease' | 2 | LEOSS \| PaCovid |
| 'Multilobar lung infiltrate' | 2 | CAPNETZ \| LEOSS |
| 'muscle pain' | 2 | GECCO \| LEOSS |
| Myalgia | 2 | PaCovid \| Brazilian Variables |
| 'myocardial infarction' | 2 | GECCO \| LEOSS |
| Myocarditis | 2 | LEOSS \| Brazilian Variables |
| Myositis | 2 | LEOSS \| PaCovid |
| 'nasopharyngeal swab specimen' | 2 | LEOSS \| PaCovid |
| 'Nervous System Disorder' | 2 | CAPNETZ \| Brazilian Variables |
| Neuralgia | 2 | LEOSS \| PaCovid |
| Neurology | 2 | CAPNETZ \| IBM Explorys |
| neuropathy | 2 | CAPNETZ \| PaCovid |
| Neurosurgery | 2 | CAPNETZ \| IBM Explorys |
| nitrite | 2 | CAPNETZ \| MIMIC-III |

| | | |
|---|---|---|
| 'Non-nucleoside Reverse Transcriptase Inhibitor' | 2 | CAPNETZ \| LEOSS |
| 'Not Done' | 2 | GECCO \| LEOSS |
| 'Not Performed' | 2 | GECCO \| CAPNETZ |
| 'Nucleoside Reverse Transcriptase Inhibitor' | 2 | CAPNETZ \| LEOSS |
| 'Number of Hospitalization Days' | 2 | CAPNETZ \| MC-19 |
| 'nursing home' | 2 | CAPNETZ \| IBM Explorys |
| nystagmus | 2 | LEOSS \| PaCovid |
| Obesity | 2 | MC-19 \| Brazilian Variables |
| 'Obesity Hypoventilation Syndrome' | 2 | LEOSS \| PaCovid |
| 'Obstetrics and Gynecology' | 2 | CAPNETZ \| IBM Explorys |
| Oncology | 2 | GECCO \| IBM Explorys |
| Ophthalmology | 2 | CAPNETZ \| IBM Explorys |
| 'Oral and maxillofacial surgery' | 2 | CAPNETZ \| IBM Explorys |
| 'Oral Route of Administration' | 2 | CAPNETZ \| Erlangen |
| oseltamivir | 2 | GECCO \| LEOSS |
| 'Other autoimmune disease' | 2 | CAPNETZ \| Brazilian Variables |
| 'Other cardiovascular disease' | 2 | GECCO \| LEOSS |
| 'Other complication' | 2 | CAPNETZ \| PaCovid |
| 'Other diseases or conditions' | 2 | CAPNETZ \| Brazilian Variables |
| 'Other macrolide' | 2 | CAPNETZ \| LEOSS |
| 'Other respiratory disorders' | 2 | GECCO \| CAPNETZ |
| 'Other Route of Administration' | 2 | CAPNETZ \| Erlangen |

| | | |
|---|---|---|
| 'Other symptoms and signs involving cognitive functions and awareness' | 2 | GECCO \| LEOSS |
| 'Outcome at discharge' | 2 | GECCO \| PaCovid |
| 'outpatient encounter' | 2 | CAPNETZ \| IBM Explorys |
| oxacillin | 2 | Erlangen \| MIMIC-III |
| 'Oxygen measurement, partial pressure, arterial' | 2 | GECCO \| Aachen |
| 'Oxygen Therapy' | 2 | PaCovid \| Brazilian Variables |
| 'Oxygenation index' | 2 | Frankfurt \| Aachen |
| pancreas | 2 | GECCO \| LEOSS |
| paresis | 2 | LEOSS \| PaCovid |
| 'Parkinson's disease' | 2 | GECCO \| PaCovid |
| 'Patient Identifier' | 2 | Erlangen \| IBM Explorys |
| 'Patient Readmission' | 2 | CAPNETZ \| MIMIC-III |
| 'Patient Status' | 2 | CAPNETZ \| LEOSS |
| Pediatrics | 2 | GECCO \| IBM Explorys |
| 'pericardial effusion' | 2 | LEOSS \| PaCovid |
| pericarditis | 2 | LEOSS \| Brazilian Variables |
| phosphate | 2 | PaCovid \| MIMIC-III |
| 'picogram per milliliter' | 2 | CAPNETZ \| MC-19 |
| Plasmapheresis | 2 | LEOSS \| MIMIC-III |
| 'Platelet Count' | 2 | Aachen \| MIMIC-III |
| 'polyarteritis nodosa' | 2 | CAPNETZ \| LEOSS |
| 'polymerase chain reaction' | 2 | LEOSS \| PaCovid |
| polymyositis | 2 | CAPNETZ \| LEOSS |
| Pregnant | 2 | CAPNETZ \| MIMIC-III |
| 'presence of pathogen' | 2 | CAPNETZ \| PaCovid |

| | | |
|---|---|---|
| 'Pressure controlled ventilation' | 2 | LEOSS \| PaCovid |
| Procedure | 2 | Erlangen \| IBM Explorys |
| 'Productive cough' | 2 | GECCO \| LEOSS |
| prophylaxis | 2 | GECCO \| Erlangen |
| propofol | 2 | Erlangen \| MIMIC-III |
| 'protease inhibitor' | 2 | CAPNETZ \| LEOSS |
| 'Protein C, functional assay' | 2 | PaCovid \| MIMIC-III |
| 'Protein S, functional assay' | 2 | PaCovid \| MIMIC-III |
| 'Proteus mirabilis' | 2 | CAPNETZ \| Erlangen |
| 'psychiatric disorder' | 2 | GECCO \| CAPNETZ |
| Psychiatry | 2 | CAPNETZ \| IBM Explorys |
| 'Pulmonary Compliance' | 2 | Aachen \| MIMIC-III |
| 'Pulmonary Consolidation' | 2 | MC-19 \| LEOSS |
| 'Pulmonary Infiltrate' | 2 | CAPNETZ \| PaCovid |
| 'Pyramidal symptoms' | 2 | LEOSS \| PaCovid |
| Radiology | 2 | GECCO \| IBM Explorys |
| 'Rapid shallow breathing index' | 2 | Erlangen \| MIMIC-III |
| 'RASS - Score' | 2 | Erlangen \| Brazilian Variables |
| 'Rectal Route of Administration' | 2 | CAPNETZ \| Erlangen |
| 'Reduced visual acuity' | 2 | LEOSS \| PaCovid |
| Referral | 2 | GECCO \| PaCovid |
| 'Referral to palliative care service' | 2 | GECCO \| PaCovid |
| remdesivir | 2 | GECCO \| LEOSS |
| remission | 2 | GECCO \| LEOSS |
| 'Remission phase' | 2 | GECCO \| LEOSS |

| | | |
|---|---|---|
| 'Renal Replacement Therapy' | 2 | CAPNETZ \| Erlangen |
| Residence | 2 | CAPNETZ \| LEOSS |
| Respiration | 2 | Erlangen \| LEOSS |
| 'Respiratory Arrest' | 2 | Brazilian Variables \| MIMIC-III |
| 'Respiratory outcome' | 2 | GECCO \| PaCovid |
| 'Respiratory Therapy' | 2 | GECCO \| LEOSS |
| 'rheumatic disease' | 2 | GECCO \| LEOSS |
| ribavirin | 2 | LEOSS \| MIMIC-III |
| 'Richmond Agitation-Sedation Scale Clinical Classification' | 2 | GECCO \| PaCovid |
| Rigors | 2 | GECCO \| MIMIC-III |
| Rituximab | 2 | LEOSS \| MIMIC-III |
| rivaroxaban | 2 | GECCO \| LEOSS |
| ruxolitinib | 2 | GECCO \| LEOSS |
| sarcoidosis | 2 | CAPNETZ \| LEOSS |
| 'SARS-CoV-2 PCR Test Result' | 2 | GECCO \| PaCovid |
| Secukinumab | 2 | GECCO \| LEOSS |
| Sedation | 2 | CAPNETZ \| Brazilian Variables |
| sedative | 2 | Erlangen \| PaCovid |
| 'Sensory impairment' | 2 | LEOSS \| PaCovid |
| Sepsis | 2 | GECCO \| Brazilian Variables |
| 'septic shock' | 2 | GECCO \| LEOSS |
| Severe | 2 | GECCO \| IBM Explorys |
| 'Shunt Device' | 2 | Erlangen \| MIMIC-III |

| | | |
|---|---|---|
| 'Sjogren's syndrome' | 2 | CAPNETZ \| LEOSS |
| 'small intestine' | 2 | GECCO \| LEOSS |
| 'sodium-glucose transport protein subtype 2 inhibitor' | 2 | LEOSS \| PaCovid |
| 'Soluble Interleukin 2 Receptor Measurement' | 2 | GECCO \| LEOSS |
| 'Spontaneous respiration' | 2 | Erlangen \| PaCovid |
| 'Stage 5 chronic kidney disease' | 2 | GECCO \| Erlangen |
| 'Staphylococcus aureus' | 2 | CAPNETZ \| PaCovid |
| statin | 2 | LEOSS \| PaCovid |
| 'Stem Cell Transplantation' | 2 | CAPNETZ \| LEOSS |
| 'Streptococcus pneumoniae' | 2 | CAPNETZ \| PaCovid |
| 'Subcutaneous Route of Administration' | 2 | CAPNETZ \| Erlangen |
| 'supplemental oxygen therapy' | 2 | Erlangen \| LEOSS |
| 'Swab finding' | 2 | GECCO \| MC-19 |
| 'Symptom Onset' | 2 | MC-19 \| PaCovid |
| 'Synchronized intermittent mandatory ventilation' | 2 | LEOSS \| Brazilian Variables |
| 'Takayasu's arteritis' | 2 | CAPNETZ \| LEOSS |
| 'temporal arteritis' | 2 | CAPNETZ \| LEOSS |
| Text | 2 | GECCO \| Erlangen |
| 'Text value' | 2 | Erlangen \| IBM Explorys |
| 'Therapeutic Apheresis' | 2 | GECCO \| LEOSS |
| 'Therapeutic Hydrocortisone' | 2 | GECCO \| MIMIC-III |
| 'Thrombotic microangiopathy' | 2 | GECCO \| LEOSS |
| 'Timestamp Data Type' | 2 | Brazilian Variables \| IBM Explorys |

| | | |
|---|---|---|
| Tirofiban | 2 | GECCO \| MIMIC-III |
| Tocilizumab | 2 | MC-19 \| LEOSS |
| 'total serum bilirubin level' | 2 | GECCO \| LEOSS |
| Transplants | 2 | GECCO \| LEOSS |
| 'Transportation of Patients' | 2 | Erlangen \| Brazilian Variables |
| 'Traumatic injury' | 2 | Erlangen \| LEOSS |
| treatment | 2 | GECCO \| MC-19 |
| 'treatment end date' | 2 | CAPNETZ \| IBM Explorys |
| 'Treatment Ongoing' | 2 | CAPNETZ \| PaCovid |
| Troponin | 2 | Aachen \| MIMIC-III |
| tuberculosis | 2 | CAPNETZ \| Brazilian Variables |
| 'Tumor necrosis factor alpha inhibitor' | 2 | GECCO \| LEOSS |
| 'Type 3 diabetes mellitus' | 2 | GECCO \| LEOSS |
| 'type I diabetes mellitus' | 2 | GECCO \| CAPNETZ |
| 'type II diabetes mellitus' | 2 | GECCO \| CAPNETZ |
| 'Uncomplicated phase' | 2 | GECCO \| LEOSS |
| 'unprotected coitus' | 2 | CAPNETZ \| LEOSS |
| Unspecified | 2 | GECCO \| PaCovid |
| 'Urinary pneumococcal antigen test' | 2 | CAPNETZ \| PaCovid |
| 'Urine Albumin' | 2 | PaCovid \| MIMIC-III |
| 'urine sodium level' | 2 | PaCovid \| MIMIC-III |
| 'urine total protein level' | 2 | LEOSS \| MIMIC-III |
| Urology | 2 | CAPNETZ \| IBM Explorys |
| 'user identifier' | 2 | Erlangen \| IBM Explorys |
| Ustekinumab | 2 | GECCO \| LEOSS |

| | | |
|---|---|---|
| 'Vascular Surgery' | 2 | CAPNETZ \| IBM Explorys |
| vasculitis | 2 | GECCO \| LEOSS |
| 'Venous Thrombosis' | 2 | GECCO \| LEOSS |
| 'Ventilation mode' | 2 | Erlangen \| PaCovid |
| Ventilator | 2 | Erlangen \| MIMIC-III |
| 'Ventilator delivered tidal volume' | 2 | Aachen \| MIMIC-III |
| 'Ventilator Weaning' | 2 | Erlangen \| MIMIC-III |
| Virology | 2 | GECCO \| Erlangen |
| 'Visual impairment' | 2 | LEOSS \| PaCovid |
| 'vital sign' | 2 | GECCO \| Erlangen |
| 'Vitek System' | 2 | CAPNETZ \| Erlangen |
| 'volume controlled ventilation' | 2 | LEOSS \| PaCovid |
| water | 2 | Erlangen \| MIMIC-III |
| Worse | 2 | CAPNETZ \| MC-19 |
| 'Wound Discharge' | 2 | Erlangen \| MIMIC-III |
| 'zinc atom' | 2 | GECCO \| LEOSS |
| | | |
| Total | 1982 | |

## Supplementary Table 3: Mappings to OMOP

| Internal Variable | OMOP Variable |
|---|---|
| blood sample collecting | Blood sample taken |
| Actual Date of Delivery | Delivery date |
| Oxygen Consumption | Oxygen consumption |
| Bilirubin:MCnc:Pt:CSF:Qn | Bilirubin |
| Epilepsie | Epilepsy |
| oxacillin | Oxacillin |

| | |
|---|---|
| Intensive Care Units, Neonatal | Neonatal intensive care unit |
| Nasal Route of Administration | Nasal route of administration |
| Leucine Crystal Measurement | Leucine crystal |
| Chlamydia pneumoniae | Chlamydia pneumoniae |
| Fever greater than 100.4 Fahrenheit / 38° Celsius (finding) | Fever greater than 100.4 Fahrenheit |
| Transplant Waiting List | On transplant waiting list |
| Enteral Nutrition | Enteral nutrition |
| Human coronavirus OC43 | Human coronavirus OC43 |
| Simplified Acute Physiology Score | Simplified Acute Physiology Score |
| Acute Myocardial Infarction | Acute myocardial infarction |
| Date of observation | Date |
| amikacin | Amikacin |
| Oxygenation index | Oxygenation index |
| Bilirubin:MCnc:Pt:Periton fld:Qn | Bilirubin |
| Agent Given Within 30 Days | Ascites within 30 days |
| Interleukin 6 (highest) | Interleukin 6 |
| Leukocyte Cell Clumps Measurement | Leukocyte clumps |
| Lepirudin | Lepirudin |
| Chloroquine Phosphate | Chloroquine Phosphate |
| Anosmia | Anosmia |
| date of diagnosis | Earliest date of diagnosis |
| Transport mode to hospital | Transport mode to hospital |
| Enteral Tube Feeding | Enteral tube feeding |
| Human metapneumovirus | Human metapneumovirus infection |
| Fatty Casts | Fatty Casts |
| Sinus Rhythm | Sinus rhythm |
| respiratory failure | respiratory failure |
| amlodipine | amlodipine |
| Bilirubin:MCnc:Pt:Plr fld:Qn | Bilirubin |
| throat | throat |
| Nasogastric Tube | Nasogastric tube |
| Wheezing | Wheezing |
| prothrombin time | Prothrombin time |

| | |
|---|---|
| Sputum purulent | Purulent |
| Traumatic injury | Traumatic injury of oculomotor nerve |
| disease | disease |
| Siponimod | Siponimod |
| Acute Respiratory Failure | OMOP Acute Respiratory Failure 1 |
| Date of procedure | Date of procedure |
| ammonia | Ammonia |
| International Classification of Diseases, Ninth Revision | International Classification of Diseases, Ninth Revision, Chinese Edition, Procedures |
| PCR | PCR |
| Blood total carbon dioxide (calculated) | Blood total carbon dioxide (calculated) |
| oxygen saturation | oxygen saturation |
| toxoplasmosis | Toxoplasmosis |
| Natalizumab | Natalizumab |
| Cholestasis | Cholestasis |
| Diarrhea (finding) | Diarrhea |
| Feces | Feces |
| Site of shunt | Site of shunt |
| injection | Injection |
| Acute kidney injury | Acute kidney injury warning stage |
| Headache | Headache |
| International Classification of Diseases, Tenth Revision | International Classification of Diseases, Tenth Revision, Clinical Modification (NCHS) |
| Body temperature/Intravascular/Specific body temperature | Body temperature |
| tuberculosis | Tuberculosis |
| thrombin | thrombin |
| Leukocytes in urine | Leukocytes in urine |
| Cholesterol Crystal Measurement | Cholesterol content measurement |
| Bivalirudin | Bivalirudin |
| Muscle pain (finding) | Muscle pain |
| Treatment duration | Treatment duration |
| Fungus Present | Fungus |
| Hyaline casts | Hyaline casts |

| | |
|---|---|
| Fedratinib | Fedratinib |
| Size of catheter | Size of catheter |
| phosphate | Phosphate |
| Acute renal failure with need for dialysis | Acute renal failure |
| amphotericin B | amphotericin B; parenteral |
| International Normalized Ratio (highest) | International Normalized Ratio |
| Pain intensity | Pain intensity |
| vancomycin | vancomycin |
| Cholesterol to HDL-Cholesterol Ratio Measurement | High density lipoprotein/total cholesterol ratio measurement |
| Fatigue | Fatigue |
| Trichomonas | Trichomonas |
| Graft Versus Host Disease | Graft versus host disease |
| Hyperreflexia | Hyperreflexia |
| Immunization | Immunization |
| Tumor necrosis factor alpha (TNF-alpha) inhibitors | Tumor necrosis factor alpha (TNF-a) inhibitors |
| Skin Care | Skin care |
| inorganic phosphate | Plasma inorganic phosphate measurement |
| Pulmonary Function Test | Pulmonary Function Test with Flow-Volume Curve |
| analgesic | Analgesic |
| thrombocytopenia | Thrombocytopenia |
| Levodopa | Levodopa |
| Citrobacter freundii | Citrobacter freundii |
| Asymptomatic | Asymptomatic |
| Tricyclic antidepressant screen | Tricyclic Antidepressant |
| Granuloma | Granuloma |
| Hypersegmented Neutrophil Measurement | Hypersegmented neutrophil count, blood |
| allergy | Allergy |
| C-Reactive Protein (highest) | C-Reactive Protein |
| Feel Too Tired to do Things I Like to Do | I was too tired to do things outside in past 7 days |
| Skin Manifestations | Skin Manifestations |
| anion gap | Anion gap |

| | |
|---|---|
| Palliative care team | Palliative care Team |
| Lewy body dementia | Lewy body dementia |
| Rivaroxaban | Rivaroxaban |
| Chronic myocardial infarction | Myocardial infarction |
| triglyceride | Triglyceride |
| Hypertensive Crisis | Hypertensive crisis |
| health maintenance | History and physical examination, annual for health maintenance |
| Fetal Hemoglobin | Fetal Hemoglobin |
| Skin Temperature | Skin temperature |
| Spirometry | Spirometry |
| antacid | Antacid |
| thyroid gland disease | Thyroid gland repair |
| Need for unplanned mechanical ventilation | Mechanical ventilation |
| Apixaban | Apixaban |
| delayed | Delayed |
| Chronic respiratory failure | Chronic respiratory failure |
| Triglyceride.plr fld | Triglyceride.plr fld/Triglyceride.serum |
| antiviral agent | Antiviral agent |
| Hypochromia | Hypochromia |
| Fetal fibronectin | Fetal fibronectin |
| Skin cleansing procedure | Skin cleansing procedure |
| interstitial pneumonia | Interstitial pneumonia |
| Address | Address |
| Lactate dehydrogenase:CCnc:Pt:Body fld:Qn | Lactate dehydrogenase |
| Panic | (Panic disorder) or (panic attack) |
| thyroid peroxidase antibody measurement | Thyroid peroxidase antibody level |
| Provider type | Provider type |
| Edoxaban | Edoxaban |
| delusional disorder | Delusional disorder |
| Triiodothyronine | Triiodothyronine |
| Hypochromic Red Blood Cell | Hypochromic red blood cell |
| intestinal cancer | FH: Intestinal cancer |
| Date transplant | Date transplant |

| | |
|---|---|
| Lactate dehydrogenase:CCnc:Pt:CSF:Qn | Lactate dehydrogenase |
| Pappenheimer bodies | Pappenheimer bodies \| Blood \| Hematology and Cell counts |
| thyroid stimulating hormone | Thyroid Stimulating Hormone |
| pericarditis | Pericarditis |
| kidney neoplasm | Kidney Neoplasms |
| Calcium:MCnc:Pt:Urine:Qn | Calcium |
| Licensed Practical Nurse | Licensed Practical Nurse |
| detection of virus | Detection of virus |
| Triple Phosphate Crystal Measurement | Triple phosphate crystal |
| HIV Entry Inhibitor | Human immunodeficiency virus entry inhibitor |
| Hypogammaglobulinemia | Hypogammaglobulinemia |
| Still in Hospital | Still in hospital |
| Smoking Cessation | Smoking cessation |
| nucleoprotein (SARS-CoV-2) | Nucleoprotein |
| Maternity Ward or Nursery | Nursery |
| Lactate dehydrogenase:CCnc:Pt:Periton fld:Qn | Lactate dehydrogenase |
| anti-mitochondrial antibody | Antimitochondrial antibody |
| What type of diabetes | Diabetes type |
| tigecycline | Tigecycline |
| peripheral vascular disease | peripheral vascular disease |
| Neoplasm of uncertain or unknown behavior of kidney | Neoplasm of uncertain or unknown behavior of kidney |
| Life-threatening cardiac arrhythmia | Cardiac arrhythmia |
| Calcium:SCnc:Pt:Body fld:Qn | Calcium |
| Camostat | Camostat Mesilate 100mg1T |
| dexamethasone | Dexamethasone |
| HIV Integrase Inhibitor | HIV Integrase Inhibitors |
| Hyporeflexia | Hyporeflexia |
| Fluid Therapy | Administration of fluid therapy |
| Smooth Muscle Antibody Measurement | Smooth muscle antibody measurement |
| Admission date | Admission date |
| Day 3 | Day 3 |

| | |
|---|---|
| Lactate dehydrogenase:CCnc:Pt:Plr fld:Qn | Lactate dehydrogenase |
| antidementia drug | Antidementia drug |
| Parathyroid Hormone | Parathyroid Hormone |
| tobramycin | Tobramycin |
| peripheral venous access | Guidance for peripheral venous access |
| Neprilysin | Neprilysin |
| Linezolid | Linezolid |
| Carbon dioxide:PPres:Pt:Body fld:Qn | Carbon dioxide |
| diabetic foot | Diabetic foot |
| HIV Status | HIV Status |
| Surgical Complication | Hypertension, Surgical Complication |
| Troponin I | Troponin I |
| Hypovolemic shock | Hypovolemic shock |
| Fluoroquinolones | Fluoroquinolones |
| Soft Tissue Infection | Soft tissue infection |
| Chest pain | Chest pain |
| Day 7 | Day 7 |
| Lactate dehydrogenase:CCnc:Pt:Synv fld:Qn | Lactate dehydrogenase |
| antidepressant | Antidepressant |
| Parechovirus | Parechovirus |
| Acetaminophen | acetaminophen |
| permanent | Permanent |
| Neural Cell Adhesion Molecule 1 | Neural Cell Adhesion Molecule L1 |
| diabetic nephropathy | Diabetic nephropathy |
| Circumferential strain.max | Circumferential |
| HIV positive | HIV positive |
| Focal Fibrosis | Focal fibrosis |
| Sometimes | Sometimes |
| antigen of Legionella | Antigen of Legionella |
| Acute coronary syndrome | Acute coronary syndrome |
| tofacitinib | Tofacitinib |
| Neuralgia | Neuralgia |
| Lipase | Lipase |

| | |
|---|---|
| Captopril | Captopril |
| diabetic retinopathy | Diabetic retinopathy |
| Special care of dentures | Special care of dentures |
| Entire heart (body structure) | Entire heart |
| ischemia | Ischemia |
| Admission source | Other admission source |
| Medicine, Ayurvedic | Medicine, Ayurvedic |
| Cannula, nasal | Cannula |
| phenobarbital | Phenobarbital |
| City | City |
| Blood Pressure | Blood Pressure Determination |
| Follicle stimulating hormone | Follicle Stimulating Hormone |
| Special screening examination for other infectious diseases | Special screening examination for other infectious diseases |
| ischemic cardiomyopathy | Ischemic cardiomyopathy |
| Admission type | Admission note |
| antipsychotic drug | Antipsychotic drug |
| Leukocytes:NCnc:Pt:Plr fld:Qn | Total leukocyte count |
| Partial Pressure Arterial Oxygen to Fraction Inspired Oxygen Ratio 200 mmHg | Partial pressure arterial oxygen/fraction inspired oxygen ratio |
| Level of Consciousness | Level of consciousness |
| phenytoin | phenytoin |
| total calorie intake level | Dietary calorie intake |
| Neurologic Manifestations | Neurologic Manifestations |
| Chloride:SCnc:Pt:BldA:Qn | Chloride |
| City of residence | City of residence |
| Hepatitis | Hepatitis |
| Type 2 diabetes mellitus with multiple complications | Type 2 diabetes mellitus with multiple complications |
| Fondaparinux | Fondaparinux |
| Specific Gravity | Specific Gravity |
| itraconazole | Itraconazole |
| Dehydration | Dehydration |
| appointment | Appointment |

| | |
|---|---|
| Leukocytes:NCnc:Pt:Urine:Qn | Leukocytes |
| Bite cells | Bite cells |
| Quinidine | Quinidine |
| toxemia | Toxemia |
| Neurosurgery | Neurosurgery |
| Chloride:SCnc:Pt:Periton fld:Qn | Chloride |
| Health Care Provider | Health care provider |
| Memory impairment | Memory impairment |
| Hepatitis C Virus Positive | Hepatitis C virus |
| Type 2 diabetes mellitus without complication | Type 2 diabetes mellitus without complication |
| Forced Expiratory Volume in 1 Second | Percent of baseline forced expiratory volume in 1 second |
| site of infection | Infection by site |
| juvenile rheumatoid arthritis | Monarticular juvenile rheumatoid arthritis |
| apraxia | Apraxia |
| Low molecular weight heparin, subtherapeutic dose | Low molecular weight heparin |
| dialysate flow | Dialysate flow rate |
| Bleeding Time | Bleeding Time |
| Chloride:SCnc:Pt:Plr fld:Qn | Chloride |
| Provider role | Provider role |
| Often Have Trouble Breathing | Do you have trouble concentrating |
| Forced Expiratory Volume in 1 Second to Forced Vital Capacity Ratio Measurement | Forced expiratory volume in one second/Forced vital capacity ratio pre steroids |
| Did the patient participate in an interventional clinical trial? | Invitation to participate in clinical trial |
| apremilast | Apremilast |
| Low molecular weight heparin, therapeutic dose | Low molecular weight heparin |
| Tetrahydrocannabinol | Tetrahydrocannabinol |
| Adenoviridae | Adenoviridae Infections |
| RASS - Score | Richmond agitation-sedation scale |
| Blinatumomab | Blinatumomab |
| tracheal cannula | Tracheal |

| | |
|---|---|
| Neutrophils in sputum | Neutrophils |
| health status | Personal health status |
| Chloride measurement, body fluid | Chloride measurement, body fluid |
| Refreshing Sleep | Describe refreshing sleep |
| Forced Vital Capacity | Forced vital capacity |
| Lymphadenopathy | Lymphadenopathy |
| ketone | Ketone |
| spike glycoprotein (SARS-CoV-2) | Spike Glycoprotein, Coronavirus |
| Nausea | Nausea |
| arachidonic acid | Arachidonic acid |
| Patient Identifier | Patient identifier |
| Thrombolytic Therapy | Thrombolytic therapy |
| Myalgia | Myalgia |
| transfusion | Transfusion |
| pimecrolimus | pimecrolimus |
| Never | Never |
| immunosuppression | Immunosuppression |
| Cholesterol:MCnc:Pt:Periton fld:Qn | Cholesterol |
| Coronary heart disease 10Y risk | Coronary heart disease 10Y risk |
| Hypercholesterolemia | Hypercholesterolemia |
| Tyrosine Crystal Measurement | Tyrosine |
| Foreign Body | Foreign body |
| ketone body | Ketone body |
| diethylene glycol | Diethylene glycol |
| Delivered oxygen flow rate | Oxygen flow rate |
| argatroban | Argatroban |
| Magnesium:SCnc:Pt:Body fld:Qn | Magnesium |
| Angina pectoris | Angina pectoris |
| Radiation Oncology | Radiation Oncology |
| Blood Chemistry Measurement | Blood chemistry (& serum test) |
| piperacillin | Piperacillin |
| Cholesterol (Body fld) [Mass/Vol] | Cholesterol |
| Advance Care Planning | Advance care planning |

| | |
|---|---|
| Framingham Heart Study Cardiovascular Disease 10-Year Risk Score Clinical Classification | Framingham Cardiovascular Disease 10 year risk score calculator |
| digestive system symptom | Digestive system symptom |
| Dental Care | Dental Care |
| arrhythmogenic right ventricular cardiomyopathy | Arrhythmogenic right ventricular cardiomyopathy |
| Medicine, Chinese Traditional | Medicine, Chinese Traditional |
| Angiography | Angiography |
| Radiation Therapy | Radiation therapy |
| transmural | transmural |
| dizziness | Dizziness |
| Patient Observation | Patient observation over time |
| Aggressive Non-Hodgkin Lymphoma | Non-Hodgkin lymphoma (category) |
| Sore Throat | Sore throat |
| Congestive heart failure | Congestive heart failure monitoring |
| digoxin | Digoxin |
| Interferon Alpha | Interferon alpha |
| Angiotensin II Receptor Antagonist | Angiotensin II receptor antagonist |
| New or direct oral anticoagulants | Oral anticoagulants |
| piperacillin-sulbactam | Piperacillin+Sulbactam |
| Patient transfer to another hospital | Patient transfer to another hospital |
| Cholesterol (Pleur fld) [Mass/Vol] | Cholesterol |
| Visual Disturbance | Visual disturbance |
| Advance Directive | Advance directive |
| Immunodeficiency | Immunodeficiency |
| Unilateral | Unilateral |
| dihydralazine | Dihydralazine |
| Interferon Beta | Interferon beta |
| lab test result date | Date and time lab result reported |
| atherosclerosis | Generalized atherosclerosis |
| Methadone:SCnc:Pt:Urine:Qn | Methadone |
| Angiotensin II Type 1 Receptor Blockers | Angiotensin 2 Type 1 Receptor Antagonists |
| Nivolumab | Nivolumab |

| | |
|---|---|
| Ig kappa light chain protein | Immunoglobulin kappa light chain gene |
| Chronic obstructive pulmonary disease with (acute) exacerbation | Chronic obstructive pulmonary disease with (acute) exacerbation |
| aphasia | Aphasia |
| protein measurement | Protein measurement |
| Patient summary | Patient summary |
| Unit of Measurement | Patient summary |
| Free Estriol Measurement | Estriol |
| Vaskulitiden | Vasculitis |
| dimenhydrinate | Dimenhydrinate |
| Dermatology | Dermatology |
| laboratory test LOINC identifier | Laboratory test |
| atrioventricular block | Atrioventricular block |
| Microscopic observation:Prid:Pt:XXX:Nom:Wright stain | Microscopic observation |
| continuous positive airway pressure | Continuous Positive Airway Pressure |
| Antiretroviral Therapy | Antiretroviral therapy |
| Rapid Sequence Induction and Intubation | Rapid sequence induction |
| trimethoprim | Trimethoprim |
| piritramide | piritramide |
| Speech | Speech |
| Altered sense of smell or taste | Loss of smell or taste |
| African American | African American |
| Chronicity | Chronicity |
| kidney failure | Kidney Failure |
| testosterone | Testosterone |
| Hospitalization | Hospitalization |
| dimethyl fumarate | Dimethyl fumarate |
| mild cognitive impairment | Mild cognitive impairment |
| laboratory test result normal range lower bound | Laboratory test result borderline |
| autoimmune disease of the nervous system | Autoimmune Diseases of the Nervous System |
| Glucose:MCnc:Pt:CSF:Qn | Glucose \| Cerebral spinal fluid |
| Antitubercular Agent | Antitubercular agent |

| | |
|---|---|
| Rapid shallow breathing index | Rapid shallow breathing index \| Respiratory system \| Respiratory measures and Ventilator management |
| trochlear nerve disease | Trochlear Nerve Diseases |
| Spherocyte | Spherocyte |
| plasma cell | Plasma cell |
| Immature Granulocyte | Immature granulocyte |
| Cocaine measurement, urine | Cocaine measurement, urine |
| earache | Earache |
| Age at starting smoking | Age at starting smoking |
| Unspecified kidney failure | Unspecified kidney failure |
| Free Testosterone Measurement | Urine free testosterone measurement |
| dipeptidyl peptidase-4 inhibitor | Dipeptidyl peptidase IV inhibitor |
| autoimmune hepatitis | Autoimmune hepatitis |
| Peak Inspiratory Pressure | Peak inspiratory pressure |
| Antiviral Therapy | Antiviral therapy |
| Rarely | Rarely |
| tube | tube |
| Blood culture holder | Blood culture analyzer |
| Spontaneous | Spontaneous |
| platelet aggregation inhibitor | Platelet aggregation inhibitor |
| Collection date:TmStp:Pt:Urine:Qn | Collection date |
| feeling exhausted | Quickly exhausted |
| Unsteady gait | Unsteady gait [Minimum Data Set] |
| Immunosuppressive Therapy | Immunosuppressive therapy |
| Free Thyroxine Index | Free thyroxine index |
| diuretic | Diuretic |
| lactulose | Lactulose |
| Chill (finding) | Chill |
| Diabetes type | Diabetes type |
| polyneuropathy | Polyneuropathy |
| azathioprine | Azathioprine |
| NIH Stroke Scale (NIHSS) | National Institutes of Health Stroke Scale (NIHSS) score |

| | |
|---|---|
| Peak pressure | Peak pressure |
| Bleeding (finding) | Bleeding |
| Ravulizumab | Ravulizumab |
| Spontaneous tidal volume | Spontaneous tidal volume |
| Immunoglobulin A vasculitis | Immunoglobulin A vasculitis |
| skin lesion | Skin lesion |
| Hard coronary heart disease 10Y risk | Hard coronary heart disease 10Y risk |
| Upadacitinib | Upadacitinib |
| Thyroxine | Thyroxine |
| dopamine agonist | Dopamine agonist |
| Diagnosis-Related Groups | Diagnosis-related group (CMS) |
| aztreonam | Aztreonam |
| Pegcetacoplan | pegcetacoplan |
| Weaning | Weaning |
| vascular disease | Peripheral vascular disease |
| Body Surface Area | Body surface area |
| Sports | Sports |
| sneezing | Sneezing |
| Air Trapping | Air trapping |
| Patient data | Patient data |
| Urapidil | Urapidil |
| Incision and Drainage | Incision AND drainage |
| Free Thyroxine Measurement | Free thyroxine measurement |
| latex allergy | Latex allergy |
| b9 folate | Vitamin B9 (Folate) intake |
| Pembrolizumab | Pembrolizumab |
| Lung Biopsy | lung biopsy |
| Raynaud disease | Raynaud Disease |
| unprotected coitus | Unprotected intercourse |
| Body fluid | Body fluid |
| Inactive | Inactive |
| Staphylococcus aureus as the cause of diseases classified to other chapters | Staphylococcus aureus as the cause of diseases classified to other chapters |
| pollen allergy | Allergy to pollen |

| | |
|---|---|
| Creatine kinase-MB in reference to indicated Creatine kinase (highest) | Creatine kinase |
| Airway Plateau Pressure | Airway plateau pressure |
| Stroke 10Y risk | Stroke 10Y risk |
| Urea Nitrogen | Urea nitrogen |
| Total Protein Measurement | Total protein measurement |
| Free fatty acids measurement | Free fatty acids measurement |
| Weaning failure | Weaning |
| leflunomide | Leflunomide |
| bacteremia | Bacteremia |
| Pencil cell | Pencil cell |
| Organizing pneumonia | Cryptogenic organizing pneumonia |
| clinical history | Finding / observation / clinical history |
| urinary bladder cancer | Urinary Bladder Neoplasms |
| Bordetella pertussis | Bordetella pertussis |
| Incomprehensible speech | Disturbance in speech |
| polycystic kidney disease | Polycystic kidney disease |
| squamous epithelial cell | Squamous epithelial cell |
| dose | dose |
| Creatine kinase (highest) | Creatine kinase |
| Airway pressure delta | Airway pressure delta |
| medical history | Documentation of past medical history |
| Uric Acid Crystal Measurement | Uric acid content measurement |
| Measurement of protein in cerebrospinal fluid specimen | Measurement of protein in cerebrospinal fluid specimen |
| Weaning successful | Weaning |
| Depression | Depression |
| Dialysis Fluid | Dialysis fluid |
| bacterial pneumonia | Bacterial pneumonia |
| Operation (in the last 3 months) | Last took within the past 3 months |
| Discharge disposition | Deprecated Discharge disposition |
| Reason for initial visit for clinical assessment | Reason for initial visit for clinical assessment |
| urine bilirubin level | Urine bilirubin level |

| | |
|---|---|
| Brain natriuretic peptide | Brain natriuretic peptide measurement |
| Nodular lesions | Nodular |
| polymyalgia rheumatica | Polymyalgia rheumatica |
| pre-existing condition | Pre-existing condition |
| Squamous epithelial cells in bronchoalveolar lavage fluid | Epithelial cells.squamous [Presence] in Bronchoalveolar lavage |
| Creatinine:MCnc:Pt:Plr fld:Qn | Creatinine |
| Albuterol Metered Dose Inhaler | albuterol Metered Dose Inhaler |
| Urinary Tract Infection | Urinary tract infection |
| hormone | Hormone |
| Fresh Frozen Plasma Transfusion | Transfusion of fresh frozen plasma |
| left ventricular end-diastolic volume | Left ventricular End-diastolic volume by US |
| band form neutrophil | Band neutrophil |
| Opiates:MCnc:Pt:Urine:Qn:Screen | Opiates |
| Pending | Pending |
| Finding | Finding |
| COPD | COPD |
| urine chloride level | Urine chloride level |
| Non-invasive blood pressure | Non-invasive blood pressure |
| posaconazole | Posaconazole |
| Squamous epithelial cells in sputum | Squamous epithelial cell |
| Creatinine:MCnc:Pt:Synv fld:Qn | Creatinine |
| disorientation | Disorientation |
| Alcohol Abuse | Alcohol abuse |
| Magnetic Resonance Imaging of the Heart | Magnetic resonance imaging of heart |
| Urinary oval fat bodies | Oval fat bodies (globules) \| Urine |
| Functional Fibrinogen Measurement | Fibrinogen Measurement |
| left ventricular end-systolic volume | Left ventricular End-systolic volume by US |
| Blutstrominfektionen | Infectious agent in bloodstream |
| benign essential hypertension | Benign essential hypertension |
| Osmolality:Osmol:Pt:Body fld:Qn | Osmolality |
| beta-lactam | Beta-lactam |
| Asthenia (finding) | Asthenia |
| Chloroquin | Chloroquine Oral Suspension [Chlorochin] |

| | |
|---|---|
| Autologous Transplantation | Autologous Testis Transplantation |
| Non-invasive diastolic arterial pressure | Non-invasive diastolic arterial pressure |
| Indirect | Indirect |
| positive | Positive |
| urine color | Finding of urine color |
| Standard Base Excess Measurement | Base excess measurement |
| Creatinine (Body fld) [Mass/Vol] | Creatinine |
| Little | A little |
| Alemtuzumab | Alemtuzumab |
| Heart Rhythm | Heart rhythm status |
| Urinary pneumococcal antigen test | Urinary pneumococcal antigen test |
| Influenza virus found | Influenza virus |
| adrenaline | Adrenaline |
| Fungal antibody | Fungal antibody titer measurement |
| beta-adrenergic antagonist | Adrenergic beta-Antagonists |
| Osmolality:Osmol:Pt:Periton fld:Qn | Osmolality |
| penicillin | penicillin |
| Clinical Data | Clinical data |
| Ivermectin | Ivermectin |
| Recommendation | Recommendation |
| urine glucose amount | Urine screening for glucose |
| Non-invasive mean arterial pressure | Non-invasive mean arterial pressure |
| Breathing Exercises | Breathing exercises |
| double vision | Double vision |
| Creatinine (Periton fld) [Mass/Vol] | creatinine |
| Alkaline Phosphatase | Alkaline Phosphatase |
| Urine Albumin | Urine albumin |
| age | Age |
| Diastolic arterial pressure | Diastolic arterial pressure |
| Osmolality measurement, serum | Osmolality measurement, serum |
| Penicillin G | Penicillin G |
| Live Birth | Live birth |
| Non-invasive systolic arterial pressure | Non-invasive systolic arterial pressure |

| | |
|---|---|
| Infection | Infection |
| positive controlled ventilation | Controlled ventilation |
| Standard bicarbonate measurement | Standard bicarbonate measurement |
| Dementia in Parkinson's disease | Dementia in Parkinson's disease |
| Prescribed medications | Prescribed medications |
| Mechanical Ventilation | Mechanical ventilation |
| Urine Albumin-Creatinine Ratio | Urine albumin/creatinine ratio measurement |
| leukocyte count | Leukocyte Count |
| Diazepam | Diazepam |
| Clinical Observations Data Collection Date Time | Collection date and time |
| bladder urothelial carcinoma | Bladder Urothelial Carcinoma (BLCA) |
| Penicillin G Potassium | Penicillin G potassium |
| Rectal Temperature | Rectal temperature |
| urine ketone body level | Urine ketone test |
| Broad Casts Measurement | Broad casts |
| Bacteria Present | Bacteria present |
| Staphylococcus aureus | Staphylococcus aureus |
| Urine Casts | Urine microscopy: hyaline casts |
| allotransplantation | Intestinal allotransplantation |
| Gastrin | Gastrin |
| Diclofenac | diclofenac |
| blast cell | Blast cell |
| Other complications of genitourinary prosthetic devices, implants and grafts | Other complications of genitourinary prosthetic devices, implants and grafts |
| Penicillin V Potassium | Penicillin V Potassium |
| All National Drug code | National Drug Code (FDA and manufacturers) |
| drug allergy | Drug allergy |
| European | European |
| Location | Location |
| urine myoglobin amount | Urine myoglobin level |
| Bronchiolitis | Bronchiolitis |
| Infiltrative cardiomyopathy | Infiltrative cardiomyopathy |

| | |
|---|---|
| prednisone | prednisone |
| Staphylococcus epidermidis | Staphylococcus epidermidis |
| Immune Checkpoint Inhibitor | Immune checkpoint inhibitor |
| Urine Creatinine | Urine creatinine |
| Intermediate Care Facility | Intermediate care facility |
| Gastrointestinal Diseases | Gastrointestinal Diseases |
| levofloxacin | Levofloxacin |
| None | None |
| Percent Predicted Forced Expiratory Volume in 1 Second | Percent of baseline forced expiratory volume in 1 second |
| Red eye | Red eye |
| urine osmolality | Urine osmolality |
| Influenza A Virus | Influenza A virus |
| amphetamine | amphetamine |
| Bronchoscopy with Bronchoalveolar Lavage | Bronchoscopy and bronchoalveolar lavage |
| Intracranial Hemorrhage | Intracranial hemorrhage |
| Gastrointestinal hemorrhage | Gastrointestinal hemorrhage unspecified |
| Diet | Diet |
| blood bicarbonate amount | Blood bicarbonate level |
| Peptic Ulcer | Peptic ulcer |
| Allergic Reaction | Allergic reaction |
| Visual impairment | Visual impairment |
| pleural effusion | Pleural effusion |
| urine output | Measure of urine output |
| Burn | Burn |
| Influenza B Virus | Influenza B virus |
| primary biliary cholangitis | Primary biliary cholangitis |
| Insurance type | Insurance: Insurance Type |
| Urine Pellet | Urine |
| Intubation Procedure | Intubation |
| septic shock | Septic shock |
| Dietary Supplements | Dietary supplements - other |
| allergen | Allergen |
| Reduced visual acuity | Reduced visual acuity |

| | |
|---|---|
| Burr Cell Count | Burr cell |
| primary glomerular disease | Glomerular disease |
| ampicillin-sulbactam | Ampicillin-Sulbactam Novaplus |
| State | State |
| Normal calcium level | Normal serum calcium level |
| palliative care | Palliative care |
| protein electrophoresis | Urine protein electrophoresis |
| Invasive Candidiasis | Invasive candidiasis |
| lithium | lithium |
| Dietary advice | Dietary advice |
| blood differential white blood cell count | Differential white blood cell count |
| Allergic reaction to chemical | Allergic reaction to chemical |
| C-Peptide | C-peptide |
| urine potassium level | Urine potassium level |
| primary sclerosing cholangitis | Primary sclerosing cholangitis |
| General medicine | General medicine |
| Ear, nose and throat medicine | Ear, nose and throat disorder |
| Urine Protein Electrophoresis | Urine protein electrophoresis |
| Invasive fungal infection | Invasive fungal infection |
| sleep disorder | Sleep disorder |
| blood flow rate | Blood flow rate |
| Oxygen:PPres:Pt:Body fld:Qn | Oxygen |
| dystonia | Dystonia |
| anticonvulsant | Anticonvulsant |
| Alpha-2-Antiplasmin | Alpha-2-antiplasmin |
| urine sodium level | Urine sodium level |
| C-peptide index | C-peptide |
| Reducing Substance Measurement | Reducing substance measurement |
| Lost To Follow-Up | Lost to Follow-Up |
| Ear, Nose, and Throat manifestation | Ear, Nose, Throat |
| Urine amylase | Urine amylase |
| Ischemic stroke | Ischemic stroke without residual deficits |
| Etanercept | Etanercept |

| | |
|---|---|
| Difficulty Falling Asleep | I had difficulty falling asleep in past 7 days |
| blood hemoglobin A1c level | Hemoglobin A1c |
| antifungal agent | Antifungal agent |
| alveolar macrophage | Alveolar macrophage |
| Low | Low |
| Breast Feeding | Breast Feeding |
| Referral by physician | Referral by physician |
| urine total protein level | Urine total protein |
| Infusion Procedure | Infusion |
| Stenotrophomonas maltophilia | Stenotrophomonas maltophilia |
| Not Assessed | Not assessed |
| progressive supranuclear palsy | PSP - progressive supranuclear palsy |
| Erythrocytes:NCnc:Pt:Periton fld:Qn | Erythrocytes |
| Migraine | Migraine |
| Infliximab | Infliximab |
| Diffuse myocardial fibrosis | Diffuse subretinal fibrosis |
| Coagulation Factor IX | Coagulation factor IX |
| T2 (Observed)-Weighted Imaging | T2 |
| Percent Predicted Forced Vital Capacity | Percentage of predicted forced vital capacity |
| antilipemic drug | Antilipemic agent |
| urobilinogen | Urobilinogen |
| Bronchiectasis | Bronchiectasis |
| prolactin | prolactin |
| Glasgow Coma Score eye opening subscore | Glasgow Coma Score eye opening subscore |
| Not Available | Not available |
| Steroid bolus therapy | Steroid therapy |
| Referring | Referring organization |
| Escherichia coli [E. coli ] as the cause of diseases classified elsewhere | Escherichia coli [E. coli ] as the cause of diseases classified elsewhere |
| Infusion Pumps | Infusion |
| magnesium | magnesium |
| low density lipoprotein cholesterol measurement | Low density lipoprotein cholesterol measurement |
| Digitalis | Digitalis |

| | |
|---|---|
| Coagulation Factor V | Coagulation factor V |
| potassium | potassium |
| Parkinson's disease | Parkinson's disease clinic |
| edema | Edema |
| Pericardial | Pericardial |
| anxiety | Anxiety |
| Residence | Residence |
| Low Grade Lymphoma | Low grade lymphoma |
| Refills | Refills |
| Stillbirth | Stillbirth |
| Burkholderia | Burkholderia |
| Ethanol:MCnc:Pt:Urine:Qn | Ethanol |
| Urine magnesium | Urine magnesium (& level) |
| Long-term oxygen therapy | Long-term oxygen therapy |
| Adalimumab | Adalimumab |
| Diplopia | Diplopia |
| mTOR inhibitor | MTOR inhibitor |
| Coagulation Factor VII | Coagulation factor VII |
| blood potassium level | Blood potassium level |
| chest CT | Chest CT |
| aortic valve stenosis | Aortic valve stenosis |
| Pericardial thickening | Pericardial |
| Feel Anxious | Feel anxious |
| Refuse | refuse |
| promyelocyte | Promyelocyte |
| chloride | Chloride |
| Malignant Laryngeal Neoplasm | Malignant neoplasm of laryngeal cartilage |
| Medical Record | Medical Record Number |
| Arthralgia | Arthralgia of the forearm |
| Coagulation Factor VIII | Coagulation factor VIII |
| atrial fibrillation | Atrial fibrillation |
| Not Done | Not done |
| emphysema | Emphysema |

| | |
|---|---|
| Peripheral pulse | Peripheral pulse rate taking |
| Feel Depressed | Feel depressed |
| Heparin | Heparin |
| Neutrophil granulocyte count determined? (most recent count) | Neutrophil count |
| CSF cell count | CSF (cerebrospinal fluid) cell count |
| CA-125 Measurement | Antibody to CA-125 measurement |
| valproic acid | Valproic Acid |
| Registered Nurse | Registered Nurse |
| propofol | propofol |
| Stool chloride | Chloride \| Stool |
| Glatiramer Acetate | Glatiramer Acetate |
| Golimumab | Golimumab |
| macrophage | Macrophage |
| Peripheral Arterial Occlusive Disease | Peripheral arterial occlusive disease |
| Coagulation Factor X | Coagulation factor X |
| location of death | Location of death |
| blood sodium level | Blood sodium level |
| Patient transfer, in-hospital | Patient transfer, in-hospital |
| azithromycin | Azithromycin |
| HLA-B57*01 | HLA-B57*01 |
| Peripheral sensory deficits | Peripheral sensory neuropathy |
| Feel Helpless | Do you often feel helpless |
| Dalteparin | Dalteparin |
| CA27-29 | Cancer antigen 27-29 |
| CSF cell count increased | CSF (cerebrospinal fluid) cell count |
| Inotropic Support | Inotropic support |
| proton pump inhibitor | Proton pump inhibitor |
| Gliadin IgA Antibody Measurement | Gliadin antibody, IgA measurement |
| Ferritin (highest) | Ferritin |
| Urine nitrite | Urine nitrite |
| Meningism | Meningism |
| Methicillin Resistant Staphylococcus Aureus | Methicillin resistant Staphylococcus aureus |
| Former Smoker | Former Smoker |

| | |
|---|---|
| Direct | Direct |
| magnesium sulfate | Magnesium Sulfate |
| Cohort Study | Cohort study |
| body mass index | Body mass index |
| Not Tested | Not tested |
| Feel Hopeless | Feeling hopeless |
| Low molecular weight heparin prophylaxis | Low molecular weight heparin |
| Inotuzumab Ozogamicin | Inotuzumab ozogamicin |
| Streptococcus agalactiae | Streptococcus agalactiae |
| Urine tube | Urine tube |
| Direct Bilirubin Measurement | Direct Bilirubin |
| manufacturer code | Manufacturer code |
| Hemoptysis | Hemoptysis |
| Collagen | Collagen |
| Phosphate (Body fld) [Mass/Vol] | Phosphate |
| Not at All | Not at all |
| Patient Status | Patient Status |
| Feel Worthless | Feel depressed |
| Lumbar Puncture | Lumbar Puncture |
| Cancer Other | Other cancer |
| CD15 Antigen | CD15 antibody |
| psoriatic arthritis | Psoriatic arthritis |
| Streptococcus pneumoniae | Streptococcus pneumoniae |
| Glucose-6-Phosphate Dehydrogenase Measurement | Glucose-6-phosphate dehydrogenase measurement |
| uric acid | Uric acid |
| Nausea and vomiting | Nausea and vomiting status |
| maraviroc | Maraviroc |
| Collection Date Time | Collection date and time |
| blood transfusion | Blood transfusion |
| Cardiac Catheterization | Cardiac Catheterization Procedures |
| Inpatient | Inpatient |
| psychotropic drug | Psychotropic drug use |
| Streptococcus pyogenes | Streptococcus pyogenes |

| | |
|---|---|
| Urine uric acid | Uric acid measurement, urine |
| Nervous System Disorder | Central Nervous System Disorder |
| Never Smoker | Never smoker |
| Basiliximab | Basiliximab |
| Nasal congestion (finding) | Nasal congestion |
| bronchitis | Bronchitis |
| Dead | Dead |
| brain cancer | Cancer Condition: Brain Cancer |
| Have Trouble Doing Activities with Friends | I have trouble doing all of the activities with friends that I want to do |
| CD2 molecule | CD2 |
| Inspiratory Time | Inspiratory time |
| Stress | Stress |
| Urological Manifestations | Urological Manifestations |
| Microscopic polyangiitis | Microscopic polyangiitis |
| Nucleated Red Blood Cell | Nucleated red blood cell |
| Anakinra | Anakinra |
| Lung abscess | Lung abscess |
| Comment | Comment |
| bronchopneumonia | Bronchopneumonia |
| Hospital | Hospital |
| Phoenix | Phoenix |
| Have Trouble Doing Regular Family Activities | I have trouble doing all of the family activities that I want to do |
| CD34 molecule | CD34 |
| Good | Good |
| vascular dementia | Vascular dementia |
| Urology | Urology |
| Other neurological symptoms | Other neurological conditions |
| Discharge date | Discharge date |
| Arrhythmia | Arrhythmia |
| mean corpuscular hemoglobin concentration | Erythrocyte mean corpuscular hemoglobin concentration |
| Hospital Departments | Hospital department |

| | |
|---|---|
| cardiomyopathy | Cardiomyopathy |
| Have Trouble Doing Regular Leisure Activities with Others | I have trouble doing all of my regular leisure activities with others |
| CD3 Complex | CD3 |
| Insulin | Insulin |
| vasopressin | vasopressin |
| Multilobar lung infiltrate | Multilobar lung infiltrate |
| Ustekinumab | Ustekinumab |
| Discharge time | Discharge time |
| mean corpuscular volume | Erythrocyte mean corpuscular volume |
| calcium acetate | Calcium acetate |
| catecholamine | Catecholamine |
| Physical Therapist | Physical therapist |
| neutropenia | Neutropenia |
| Glucose:MCnc:Pt:Plr fld:Qn | Glucose \| Pleural fluid |
| vasopressor agent therapy | Vasopressor therapy |
| Luteinizing hormone | Luteinizing hormone |
| Use of de-escalation technique | Use of de-escalation technique |
| Palpitations | Palpitations |
| Canakinumab | Canakinumab |
| mean platelet volume | Mean Platelet Volume |
| Disease Progression | Disease progression |
| calcium channel blocker | Calcium channel blocker |
| Subcutaneous Route of Administration | Subcutaneous route of administration |
| Lymphocyte antigen CD20 | Lymphocyte antigen CD20 |
| end date | End date |
| Myositis | Myositis |
| Insulin Lispro | Insulin Lispro |
| Pleural fluid: RBC's present | Pleural fluid: RBC's present |
| medical intervention | please double check |
| ceftriaxone | Ceftriaxone |
| I was overwhelmed by my worries | My worries overwhelmed me in past 7 days |
| Influenza | Influenza |
| Pulmonary Infiltrate | Pulmonary infiltrate |

| | |
|---|---|
| Insulin glargine | Insulin glargine |
| calcium gluconate | Calcium gluconate |
| cigarette | Cigarette |
| Glucose:SCnc:Pt:Synv fld:Qn | Glucose in synovial fluid |
| erythromycin | Erythromycin |
| cohort | cohort |
| vitamin | Vitamin |
| MELD - Score | Model for end-stage liver disease score |
| Value | Please double check |
| Reasons for treatment | Reasons for treatment |
| Documentation | documentation |
| Secukinumab | Secukinumab |
| calcium phosphate | Calcium phosphate |
| clarithromycin | clarithromycin |
| Plateau pressure | Plateau pressure |
| Always | Always |
| Interferon | Interferon |
| Churg-Strauss syndrome | Churg-Strauss Syndrome |
| vitamin D | vitamin D |
| essential tremor | Essential tremor |
| mercaptopurine | mercaptopurine |
| Drainage of bladder | Drainage of bladder |
| candesartan | Candesartan |
| Platelet Count | Platelet Count |
| Amber | Amber |
| Cigar | Cigar |
| von Willebrand Factor | von Willebrand factor |
| MIC value | MIC |
| estradiol | Estradiol |
| Nocardia | Nocardia |
| mesothelial cell | Mesothelial cell |
| Drinking | Drinking |
| Procalcitonin (PCT) (highest) | Procalcitonin (PCT) |

| | |
|---|---|
| carbamazepine | Carbamazepine |
| Platelet Distribution Width | Platelet distribution width |
| Relavant ischemia | Ischemia |
| rectum | Rectum |
| Sucrose hemolysis test | Sucrose hemolysis test |
| ethanol | Ethanol |
| Immunoglobulin A | Immunoglobulin A |
| Confirmed | Confirmed |
| Duration of inpatient stay | Duration of inpatient stay |
| metamyelocyte | Metamyelocyte |
| Ixekizumab | Ixekizumab |
| Protein C, functional assay | Protein C, functional assay |
| Entire lung (body structure) | Entire lung |
| carbapenem | Carbapenem |
| Platelet Membrane Glycoprotein IIb | Platelet Membrane Glycoprotein IIb |
| von Willebrand Factor Activity Measurement | Von Willebrand factor activity measurement |
| Parenteral Nutrition | Parenteral nutrition |
| CD4 molecule | Cluster of differentiation antigen 4 |
| red blood cell distribution width | Red blood cell distribution width |
| Sulfated DHEA Measurement | Dehydroepiandrosterone sulfate measurement |
| Vascular Surgery | Vascular Surgery |
| Glasgow Coma Score verbal response subscore | Glasgow Coma Score verbal response subscore |
| ethylenediaminetetraacetic acid | EDTA |
| congenital heart defect | Heart Defects, Congenital |
| Non-nucleoside Reverse Transcriptase Inhibitor | Non-nucleoside reverse transcriptase inhibitors |
| metformin | Metformin |
| Carotisstenose | Carotid artery stenosis |
| Protein S, functional assay | Protein S, functional assay |
| Darm | intestine |
| carbon dioxide | Carbon dioxide |
| Platelet clumps | Platelet clumps |

| | |
|---|---|
| warfarin | warfarin |
| CD57 Antigen | Antigens, CD57 |
| relapse | Relapse |
| Sulfonamide Anti-Infective Agent | Sulfonamide |
| HIV Antibody | HIV ANTIBODY |
| Macroprolactin | Macroprolactin |
| daptomycin | daptomycin |
| Vedolizumab | Vedolizumab |
| Nontuberculous Mycobacteria | Mycobacterium, non-TB |
| methadone | methadone |
| Zinc | Zinc |
| Rheumatoide Arthritis | Rheumatoid arthritis |
| Drug Interactions | Drug Interactions |
| Intensive care unit admission date | Intensive care unit (ICU) admission date |
| Protein electrophoresis, CSF | Protein electrophoresis, CSF |
| cardiac index | Cardiac index |
| Hydroxychloroquin | hydroxychloroquine |
| CD59 Glycoprotein | CD59 |
| relapsing polychondritis | Relapsing polychondritis |
| Tidal Volume | Tidal Volume |
| Venous Pressure | Venous pressure |
| export | Export |
| water | water |
| methemoglobin | Methemoglobin |
| Meningitis | Meningitis |
| Drug Intolerance | Drug Intolerance |
| Interleukin-1 | Interleukin-1 |
| Pulmonary sign AND/OR symptom | Pulmonary sign AND/OR symptom |
| Entire liver (body structure) | Entire liver |
| cardiac tamponade | Cardiac tamponade |
| Blood Gas Analysis | Blood gas analysis |
| Renal replacement therapy - Base | Renal replacement |
| Herzrhythmusstörungen | Arrhythmia |

| | |
|---|---|
| Supine Position | Supine Position |
| HIV viral load | HIV viral load |
| Mandatory tidal volume | Mandatory tidal volume |
| Venous oxygen saturation | Venous oxygen saturation |
| Nurse Practitioner | Nurse Practitioner |
| Schlafapnoe | Sleep apnoea |
| Interleukin-2 | Interleukin-2 |
| Entire pancreas (body structure) | Entire pancreas |
| carotid artery disease | carotid artery disease |
| Arterial Blood Gas Measurement | Arterial blood gas analysis |
| Darunavir | Darunavir |
| Supranuclear gaze palsy | Supranuclear gaze palsy |
| HLA-DR Antigen Type | HLA-DR antigen |
| methotrexate | Methotrexate |
| Manual Entry | Manual entry |
| Conjunctival swab | Conjunctival swab |
| RNA-directed RNA polymerase (SARS-CoV-2) | RNA-directed RNA polymerase |
| Ventilation cycle time | Ventilation cycle time |
| dermatomyositis | Dermatomyositis |
| polymerase chain reaction | Polymerase chain reaction |
| eye | Eye |
| Mental disorder (disorder) | Mental disorder |
| Drug declined by patient | Drug declined by patient |
| Interleukin-2 Receptor | Interleukin-2 receptor |
| Pleural fluid protein content measurement | Pleural fluid protein content measurement |
| Amphetamine in urine | Amphetamine in urine |
| Lopinavir und Ritonavir | LOPINAVIR AND RITONAVIR |
| respiratory quotient | Respiratory quotient |
| Supraventricular tachycardia | Supraventricular tachycardia |
| metoprolol | Metoprolol |
| Measurement of gentamicin peak concentration | Measurement of gentamicin peak concentration |
| Constipation | Constipation |

| | |
|---|---|
| fairly | not found |
| diagnosis at discharge | not found |
| SARS-CoV-2 PCR Test Result | not found |
| Immunoglobulin G | Immunoglobulin G |
| Obesity | Obesity |
| Tacrolimus | Tacrolimus |
| Entire kidney (body structure) | Entire kidney |
| cefazolin | cefazolin |
| Pleural thickening | Pleural thickening (machine translation) |
| Ampicillin | Ampicillin |
| atazanavir | Atazanavir |
| Renal replacement therapy - Laboratory | not found |
| Tocilizumab | Tocilizumab |
| respiratory symptom | respiratory symptom |
| Radiography, Abdominal | Radiography, Abdominal |
| Surgical Procedure | surgical procedure |
| Haemophilus | Haemophilus |
| Partial Pressure Arterial Oxygen to Fraction Inspired Oxygen Ratio Measurement after first intubation | not found |
| Interleukin-2 Receptor Subunit Alpha | Interleukin-2 Receptor alpha Subunit |
| Manual Therapy | Manual Therapy |
| Consulting Physician | not found |
| dioxygen | Molecular oxygen |
| Immunoglobulin M | Immunoglobulin M |
| Obesity Hypoventilation Syndrome | Obesity hypoventilation syndrome |
| African | African |
| cefepime | cefepime |
| Pneumococcal vaccine administered or previously received | Pneumococcal vaccine administered or previously received |
| Sarilumab | Sarilumab |
| familial Mediterranean fever | familial Mediterranean fever |
| respiratory tract | Respiratory tract |
| Haemophilus influenzae | Haemophilus influenzae |

| | |
|---|---|
| Interleukin 6 receptor antagonist | Interleukin 6 receptor antagonist |
| Marijuana | Marijuana |
| Ventilator | Ventilator |
| Amylase | Amylase |
| Staining of Sputum for Pneumocystis | Pneumocystis sputum stain method |
| OSAS | Obstructive sleep apnea syndrome |
| cefixime | cefixime |
| Poikilocytosis | Poikilocytosis |
| Acetylsalicylsäure | acetylsalicylic acid |
| Famotidine | Famotidine |
| Synchronized intermittent mandatory ventilation | Synchronized intermittent mandatory ventilation |
| Haemophilus parainfluenzae | Haemophilus parainfluenzae |
| Fibrin degradation products | Fibrin degradation products |
| Massive | Massive |
| Ventilator delivered tidal volume | Ventilator delivered tidal volume |
| dobutamine | Dobutamine |
| Anaerobic Bacteria | Anaerobic bacteria |
| erythrocyte | Erythrocyte |
| Loss of appetite (finding) | Loss of appetite |
| Obinutuzumab | Obinutuzumab |
| Anti-Infective Agents | Anti-infective Agent |
| Environmental allergy | Environmental allergy |
| cefotaxime | cefotaxime |
| Renin | Renin |
| Corticosteroide | Corticosteroid |
| Syndecan-1 | Syndecan-1 |
| fat-soluble vitamin | Fat-soluble vitamin |
| ribavirin | Ribavirin |
| midazolam | Midazolam |
| Internal Medicine | Internal Medicine |
| Mean inspiratory airway pressure | Mean inspiratory airway pressure |
| Localization | Localization - action |
| Superinfection | superinfection |

| | |
|---|---|
| Obstetrics and Gynecology | Obstetrics and Gynecology |
| cefotiam | Cefotiam |
| Polatuzumab Vedotin | polatuzumab vedotin |
| Ja | Yes |
| rifampicin | Rifampicin |
| Hafnia alvei | Hafnia alvei |
| anemia | anemia |
| Ventricular arrhythmia | Ventricular arrhythmia |
| Occult Blood | Occult Blood |
| Bacterial Infection | Bacterial Infection |
| Pulmonary Compliance | Pulmonary compliance study |
| cefoxitin | cefoxitin |
| Polychromasia | Polychromasia |
| Internal facility referral or transfer | Internal facility referral or transfer |
| calcium | Calcium |
| rifaximin | Rifaximin |
| Result (Pattern) | Result |
| feces osmolality | Serum osmolality |
| Haloperidol | Haloperidol |
| gentamicin | gentamicin |
| Continuous renal replacement therapy | Continuous renal replacement therapy |
| doxycycline | doxycycline |
| Occupation | Occupation |
| Dynamic Compliance | Dynamic compliance |
| cefpodoxime proxetil | cefpodoxime proxetil |
| Poor | Poor |
| Atrial natriuretic peptide | Atrial natriuretic peptide |
| Resident | Resident |
| Haptoglobin | Haptoglobin |
| Angiotensin-Converting Enzyme | Angiotensin-converting enzyme |
| Measurement of gentamicin trough concentration | Measurement of gentamicin trough concentration |
| Continuous use of oxygen therapy | Oxygen therapy - Continuous |
| Ventriculoperitoneal Shunt | Ventriculoperitoneal Shunt |

| | |
|---|---|
| IgG1 immunoglobulin complex (human) | Immunoglobulin IgG1 |
| Ocrelizumab | Ocrelizumab |
| Dysarthria | Dysarthria |
| Cystic fibrosis (disorder) | Cystic fibrosis |
| cefuroxime | cefuroxime |
| Poor appetite | POOR APPETITE |
| Reticulocyte count, automated | Blood count; reticulocyte, automated |
| Attending | Attending |
| right ventricular end-diastolic volume | Right ventricular End-diastolic volume by Imaging |
| Systemic blood pressure | Systemic blood pressure |
| Have Trouble Breathing | I have trouble breathing in the past 7 days |
| Animal dander allergy | Allergy to animal dander |
| Measurement of tobramycin peak concentration | Measurement of tobramycin peak concentration |
| enoximone | Enoximone |
| Vertigo | Vertigo |
| E-mail Address | Email address |
| Porphobilinogen Measurement | Porphobilinogen measurement |
| Atypical lymphocytes | Atypical lymphocytes |
| Reticulocyte count, manual | Reticulocyte count, manual |
| Respiration monitoring | Respiration monitoring |
| right ventricular end-systolic volume | Right ventricular End-systolic volume by Imaging |
| Have Trouble Going To Sleep | Have trouble sleeping |
| Anisocytosis | Anisocytosis |
| Measurement of tobramycin trough concentration | Measurement of tobramycin trough concentration |
| Contraindicated | Contraindicated |
| Very | Very |
| Ofatumumab | Ofatumumab |
| Asthma | Asthma |
| acute-on-chronic liver failure | Acute-On-Chronic Liver Failure |
| esophageal cancer | Esophageal cancer |
| Systemic vascular resistance index | Systemic vascular resistance index |

| | |
|---|---|
| fibrinogen measurement | Fibrinogen measurement |
| Respiratory Arrest | Respiratory Arrest |
| Have to Stop for Breath After Walking About 100 Yards on Level Ground | Do you ever have to stop for breath after walking about 100 yards, or after a few Ms, on the level |
| Anthroposophical medicine | Anthroposophical medicine |
| Measurement of vancomycin in random specimen | Measurement of vancomycin in random specimen |
| Contrast media allergy | Contrast Media Allergy |
| cellulitis | Cellulitis |
| Respiratory Pattern | Respiratory pattern |
| Calcium pyrophosphate crystal | Calcium pyrophosphate crystal |
| ristocetin | Ristocetin |
| fingolimod | fingolimod |
| esophageal varices with bleeding | Esophageal varices with bleeding |
| Very Good | Very good |
| Measurement of vancomycin peak concentration | Measurement of vancomycin peak concentration |
| Control | Control |
| Cause of Death | Cause of death |
| Often | Often |
| Angsterkrankung | Anxiety disorder |
| Positive end expiratory pressure | Positive end expiratory pressure |
| Autopsy | Autopsy |
| Calculated LDL cholesterol level | Calculated LDL cholesterol level |
| Respiratory Syncytial Virus Infection | Respiratory Syncytial Virus Infections |
| food allergy | Food allergy |
| Anti-Neutrophil Cytoplasmic Antibody Measurement | Anti-neutrophil cytoplasmic antibody screen |
| Measurement of vancomycin trough concentration | Measurement of vancomycin trough concentration |
| Echinacea | Echinacea |
| Cirrhosis | Cirrhosis |
| central venous cannula insertion | Central venous cannula insertion |
| enzyme | Enzyme |

| | |
|---|---|
| Average blood pressure | Average blood pressure |
| Surfactant/Albumin | Surfactant/Albumin |
| Respiratory Therapy | Respiratory therapy |
| fosfomycin | Fosfomycin |
| roxithromycin | Roxithromycin |
| Health record report | Health record review |
| mitoxantrone | Mitoxantrone |
| Viral antibody | Anti viral antibody |
| Medicaid | Medicaid |
| Typ 3 | Type 3 |
| Eculizumab | Eculizumab |
| extended-spectrum beta-lactamase | Extended-spectrum beta lactamase |
| Calculated Right Ventricular Ejection Fraction | Right ventricular ejection fraction |
| Sodium:SCnc:Pt:Body fld:Qn | Sodium \| Body Fluid |
| Alanine Aminotransferase | Alanine aminotransferase |
| frontotemporal dementia | Frontotemporal dementia |
| mixed connective tissue disease | Mixed connective tissue disease |
| Medical Assistant | Medical Assistant |
| Abdominal pain | Abdominal pain |
| Education Level | The Basics: Education Level |
| centromere | Centromere |
| Meprednison | meprednisone |
| Potassium Phosphate | Potassium phosphate |
| Interventional Radiology | Interventional Radiology |
| Plasmapheresis | Plasmapheresis |
| Sodium:SCnc:Pt:Periton fld:Qn | Sodium \| Peritoneal fluid |
| Albumin | Albumin |
| serum chloride level | Serum chloride level |
| ganciclovir | Ganciclovir |
| Heinz Body | Heinz Body |
| model number | Model number |
| Medical Examination | Medical examination |
| cephalosporin | cephalosporin |

| | |
|---|---|
| 25-Hydroxyvitamin D | 25-hydroxyvitamin D |
| gas gangrene | Gas gangrene |
| Route of Administration | Route of administration |
| reticulocyte count | reticulocyte count |
| Sodium:SCnt:Pt:Stool:Qn | Sodium [Moles/mass] in Stool |
| Blood Urea Nitrogen | Blood urea nitrogen |
| gastrointestinal cancer | Suspected lower gastrointestinal cancer |
| T2 mapping | Magnetic resonance imaging of brain with T2 mapping |
| Hematocrit:VFr.DF:Pt:Body fld:Qn | Hematocrit \| Body Fluid |
| monocyte count | monocyte count |
| Medical record number | Medical record number [Identifier] |
| Copeptin Measurement | Copeptin immunofluorescent assay |
| Ophthalmology | Ophthalmology |
| Arab | Arab |
| Electroencephalogram | Electroencephalogram (EEG) |
| cerebral computed tomography imaging | Cerebral cisterns \| Computed tomography |
| Intramuscular Route of Administration | Intramuscular route of administration |
| Aviptadil | Aviptadil |
| Reticulocyte Corpuscular Hemoglobin Content | Reticulocyte corpuscular hemoglobin concentration mean |
| cholesterol | Cholesterol |
| Antimicrobial susceptibility | Antimicrobial Susceptibility |
| Hematuria | Hematuria |
| Medicare | Medicare |
| Aldosteron | Aldosterone |
| Nucleoside Reverse Transcriptase Inhibitor | Nucleoside Reverse Transcriptase Inhibitors |
| delirium | Delirium |
| Electrolytes | Electrolytes |
| granulomatosis with polyangiitis | granulomatosis with polyangiitis |
| cerebrospinal fluid | Cerebrospinal fluid |
| Invasive systolic arterial pressure | Invasive systolic arterial pressure |
| Rheumatoid Factor | Rheumatoid Factor |
| Cholesterol total | Cholesterol/Total |

| | |
|---|---|
| Myokardinfarkt | myocardial infarction |
| Men who have Sex with Men | Male who has sex with men |
| Coronary Microvascular Disease | Coronary microvascular disease |
| Oral Route of Administration | Oral route of administration |
| Electronic Cigarette | Electronic cigarette |
| hematologic cancer | Hematologic cancer |
| cerebrospinal fluid chloride level | CSF (cerebrospinal fluid) chloride level |
| motor neuron disease | Motor neuron disease |
| Axillary Temperature | Axillary temperature |
| Troponin | Troponin |
| glucagon-like peptide-1 receptor agonist | Glucagon-like peptide 1 receptor agonist |
| Hemoglobin A | Hemoglobin A |
| Biological Products | Biological Products |
| Hematocrit:VFr:Pt:Plr fld:Qn | Hematocrit \| Pleural fluid |
| Metastasis | Metastasis |
| non-Hodgkin lymphoma | Non-Hodgkin lymphoma |
| total serum bilirubin level | Serum total bilirubin level |
| Oral Temperature | Oral temperature |
| Conjunctivitis (disorder) | Conjunctivitis |
| Electrophysiology | Electrophysiology |
| hemiplegia | Hemiplegia |
| chronic | Chronic |
| moxifloxacin | Moxifloxacin |
| Intravenous Route of Administration | Intravenous route of administration |
| Rhinovirus | Rhinovirus |
| TOAST Classification | TOAST |
| glucometer | glucometer |
| Hemoglobin A2 | Hemoglobin A2 |
| Hematocrit:VFr:Pt:Synv fld:Qn | Hematocrit \| Synovial fluid |
| Metastatic Malignant Neoplasm in the Brain | Metastatic malignant neoplasm to brain |
| Glucocorticoide | Glucocorticoid |
| Country of current residence | Country of current residence |
| Opioid | Opioid |

| | |
|---|---|
| Oral and maxillofacial surgery | Oral and maxillofacial surgery |
| Immunomodulation Therapy | Immunomodulatory drugs (IMiDs) |
| Elliptocyte Count | Ovalocytes \| Blood \| Hematology and Cell counts |
| levosimendan | levosimendan |
| chronic liver failure | Chronic hepatic failure |
| multiple myeloma | Multiple myeloma |
| Intraventricular pressure catheter | Intracranial pressure catheter |
| Right Ventricular Dysfunction | Right ventricular dysfunction |
| B-Cell Receptor CD22 | Lymphocyte antigen CD22 |
| Temperature of pinna | Temperature of pinna |
| glycolic acid | Glycolic acid |
| Neutrophil Band Form Count | Neutrophil band count |
| Hemoglobin C | Hemoglobin C |
| serum amyloid A | Serum amyloid A protein |
| Country of residence | County of residence |
| Oral care | Oral care |
| immunomodulator | Immunomodulator |
| Emergency Room | Emergency Room - Hospital |
| hospital ward | Hospital ward |
| chronic lymphocytic leukemia | Chronic lymphocytic leukemia |
| Invasive blood pressure | Invasive blood pressure |
| Can Do With Much Difficulty | Able to do with much difficulty |
| glycopeptide antibiotic | Glycopeptide antibiotic |
| Segmented Neutrophil Count | Segmented neutrophil count, blood |
| Hemoglobin S | Hemoglobin S genotype determination |
| Takotsubo Cardiomyopathy | Takotsubo cardiomyopathy |
| Visual Analog Pain Scale | Visual analog pain scale |
| severe acute respiratory syndrome | Severe acute respiratory syndrome |
| Metastatic Malignant Neoplasm in the Colon | Metastatic malignant neoplasm to colon |
| lung ultrasonography | Ultrasonography of lung |
| Emergency Room Visit | Emergency Room Visit |
| ibuprofen | Ibuprofen |
| chronic myeloid leukemia | Chronic myeloid leukemia |

| | |
|---|---|
| Phenprocoumon | Phenprocoumon |
| Pressure controlled ventilation | Pressure controlled ventilation |
| multiple system atrophy | Multiple system atrophy |
| Invasive diastolic arterial pressure | Invasive diastolic arterial pressure |
| Can Do With Some Difficulty | Does with some difficulty |
| Troponin T | Troponin T |
| sex hormone-binding globulin | Sex Hormone-Binding Globulin |
| Antihypertensive Therapy | Antihypertensive therapy |
| ejection fraction | Ejection Fraction |
| Creatine Kinase | Creatine kinase |
| Carbon dioxide:PPres:Pt:BldC:Qn | Carbon Dioxide \| Blood capillary |
| medical etiology | Etiology |
| glycoprotein | Glycoprotein |
| imipenem | Imipenem |
| cigarettes per day measurement | Cigarettes smoked per day |
| Invasive mean arterial pressure | Invasive mean arterial pressure |
| Can Do With a Little Difficulty | Able to do with little difficulty |
| Urea | Urea |
| shock | Shock |
| Antimicrobial Susceptibility Result | Antimicrobial susceptibility test |
| Waxy casts | Waxy casts |
| shaving | Shaving |
| Hodgkin's lymphoma | Hodgkin lymphoma |
| Metastatic Malignant Neoplasm in the Larynx | Metastatic malignant neoplasm to larynx |
| Creatine Kinase MB | CKMB - Creatine kinase MB isoenzyme |
| muscle weakness | Muscle weakness |
| Riluzole | Riluzole |
| gout | Gout |
| leukemia | Leukemia |
| ciprofloxacin | Ciprofloxacin |
| Preterm Birth | Preterm infant |
| myasthenia gravis | Myasthenia gravis |
| Can Do Without Any Difficulty | Able to do without any difficulty |

| | |
|---|---|
| base excess | base excess |
| sickle cell | Sickle cells |
| Antineoplastic chemotherapy regimen | Antineoplastic chemotherapy regimen |
| Targeted Therapy | Targeted therapy |
| Hemorrhagic Shock | Hemorrhagic shock |
| Hospice/Palliative Care Unit | Hospice/Palliative Care Unit |
| Metastatic Malignant Neoplasm in the Lung | Metastatic malignant neoplasm to lung |
| supplemental oxygen therapy | Supplemental oxygen therapy |
| Creatinine Clearance | Creatinine clearance |
| lymphoma | Lymphoma |
| Risk calculation method used | Risk calculation method used |
| lopinavir | Lopinavir |
| cladribine | Cladribine |
| mycophenolate mofetil | mycophenolate mofetil |
| immune suppressant agent therapy | Immunosuppressant drug therapy |
| Thrombocytopenia, unspecified | Thrombocytopenia, unspecified |
| Procedure | Procedure |
| eosinophil | Eosinophil |
| Hemorrhagic pleural effusion | Hemorrhagic pleural effusion |
| Antithrombin Activity Measurement | Antithrombin III activity measurement |
| Metastatic Malignant Neoplasm in the Pancreas | Metastatic malignant neoplasm to pancreas |
| Prednisolon | prednisolone |
| Creatinine urine | Creatinine - urine |
| pharyngitis | pharyngitis |
| Rituximab | Rituximab |
| Endocarditis | Endocarditis |
| lung cancer | Lung cancer |
| clamp device | Clamp, device |
| comorbidity | Comorbidities |
| Primary Care | Primary care |
| mycophenolic acid | mycophenolic acid |
| Ipilimumab | Ipilimumab |
| Order status | Order status |

| | |
|---|---|
| glomerular filtration rate | Glomerular filtration rate |
| Hemosiderin | Hemosiderin |
| Telephone Number | Telephone number |
| Apnea | Apnea |
| Metastatic Malignant Neoplasm in the Prostate Gland | Metastatic malignant neoplasm to prostate |
| Cough (finding) | Cough |
| clindamycin | Clindamycin |
| Chronic Disease | Chronic disease |
| Private Health Insurance | Private Health Insurance |
| myelocyte | Myelocyte |
| iron | iron |
| glucose | Glucose |
| Hepatitis A Virus Antibody | Antibody to hepatitis A virus |
| Metastatic Malignant Neoplasm in the Stomach | Metastatic malignant neoplasm to stomach |
| Critical Illness Myopathy | Critical illness myopathy |
| Room temperature | Room temperature |
| Organomegaly | Organomegaly |
| meropenem | Meropenem |
| liver disease | Liver disease |
| myeloid neoplasm | myeloid neoplasm |
| Triacylglycerol lipase:CCnc:Pt:Body fld:Qn | Triacylglycerol Lipase \| Body Fluid |
| Candida | Candida |
| hematocrit | Hematocrit |
| Appointment date | Appointment date |
| Temporary | Temporary |
| Metastatic Malignant Neoplasm in the Vagina | Metastatic malignant neoplasm to vagina |
| Hydrocortison | hydrocortison |
| hair care | Hair care activities |
| metamizole | metamizole |
| myocarditis | Myocarditis |
| Irritability | Irritability |

| | |
|---|---|
| Triacylglycerol lipase:CCnc:Pt:Periton fld:Qn | Triacylglycerol Lipase \| Peritoneal fluid |
| Inflammatory diseases of the central nervous system | Inflammatory diseases of the central nervous system |
| Candida albicans | Candida albicans |
| Hepatitis B Surface Antigens | HEPATITIS B SURFACE ANTIGENS |
| Term Birth | Term Birth |
| AIDS | AIDS |
| MRI contrast agent | Magnetic resonance imaging (MRI) contrast agent (machine translation) |
| Cryoglobulin | Cryoglobulin |
| Oxygen Therapy | Oxygen therapy |
| Orthopedic Surgery | Orthopedic Surgery |
| Enterobacteriaceae | Enterobacteriaceae |
| milrinone | Milrinone |
| clonidine | clonidine |
| Legionella sp Ag:PrThr:Pt:Urine:Ord | Legionella sp \| Urine |
| Triacylglycerol lipase:CCnc:Pt:Plr fld:Qn | Triacylglycerol Lipase \| Pleural fluid |
| autoimmune disease | Autoimmune disease |
| Candida glabrata | Candida glabrata |
| sodium-glucose transport protein subtype 2 inhibitor | Sodium-Glucose Cotransporter 2 Inhibitor |
| hydrogencarbonate | Bicarbonate |
| Hepatitis B Virus Antibody | Antibody to hepatitis B virus |
| Phytotherapy | Phytotherapy |
| Crystal birefringence | Crystal |
| Patient Readmission | Readmission |
| Osmolality Measurement | Osmolality measurement |
| antimicrobial susceptibility testing | Antimicrobial susceptibility test |
| Enterococcus faecalis | Enterococcus faecalis |
| co-trimoxazole | co-trimoxazole |
| myoglobin | Myoglobin |
| Isolation | Isolation |
| Triglyceride:MCnc:Pt:Periton fld:Qn | Triglyceride \| Peritoneal fluid |
| Multiple Sklerose | Multiple sclerosis |

| | |
|---|---|
| organism | Organism |
| Cannot Do | Cannot do |
| low-density lipoprotein cholesterol | LOW-DENSITY LIPOPROTEIN CHOLESTEROL |
| Hepatitis B Virus Core Antibody | Antibody to hepatitis B core antigen |
| sodium hydrogencarbonate | Sodium bicarbonate |
| Aromatherapy | Aromatherapy |
| Text value | Text value |
| Microcytic Red Blood Cell | Microcytic anemia |
| Monoclonal Antibody | Monoclonal antibody |
| Cuff pressure | Cuff pressure |
| Patient eligible for clinical trial | Patient eligible for clinical trial |
| Enterococcus faecium | Enterococcus faecium |
| coagulation | Coagulation |
| myopathy | Myopathy |
| Urea nitrogen:MCnc:Pt:Body fld:Qn | Urea Nitrogen \| Body Fluid |
| Bacteria | Bacteria |
| Cannula Device | Cannula, device |
| monocyte | Monocyte |
| Hepatitis B Virus Core IgM Antibody Measurement | Hepatitis B core antibody measurement, IgM type |
| Theophylline | Theophylline |
| head and neck cancer | Head and neck cancer |
| Glasgow coma scale | Glasgow Coma Scale |
| Other Antihypertensives | Other antihypertensives |
| neuropathy | Neuropathy |
| Isosource Energy | Isosource Energy |
| Urea nitrogen:MCnc:Pt:Periton fld:Qn | Urea Nitrogen \| Peritoneal fluid |
| Bacterial Count | Bacterial count |
| Carboxyhemoglobin | Carboxyhemoglobin |
| sodium | sodium |
| Hepatitis B Virus Surface Antibody | Antibody to hepatitis B surface antigen |
| Protein S | protein S |
| Enterovirus | Enterovirus |

| | |
|---|---|
| non-steroidal anti-inflammatory drug | Non-steroidal anti-inflammatory drug |
| JAK Inhibitor | JAK inhibitor |
| Bacterial Peritonitis | Bacterial peritonitis |
| Urea nitrogen measurement, urine | Urea nitrogen measurement, urine |
| Chronisch entzündl. Darmerkrankung | Inflammatory bowel disease |
| Carcinoembryonic Antigen Measurement | Carcinoembryonic antigen measurement |
| Hepatitis C Antibody | Hepatitis C antibody |
| Renal Replacement Therapy | Renal replacement |
| Therapeutic intervention scoring system | Therapeutic intervention scoring system |
| spinal muscular atrophy | Spinal muscular atrophy |
| Mild dementia | Mild dementia |
| Pedal edema | Edema of foot |
| movement disease | Movement Disorder |
| Eosinophil Count | Eosinophil count |
| noradrenaline | Noradrenaline |
| Influenza A (H1N1) Virus | Influenza A H1N1 virus 2009 pandemic strain present |
| Jonosteril | Jonosteril |
| Urine microscopy: cysteine crystals | Urine microscopy: cysteine crystals |
| Delivery, Obstetric | Delivery, Obstetric |
| Hemodynamics | Hemodynamics |
| Bacterial antibody | Bacterial antibody type |
| spondyloarthropathy | Spondyloarthropathy |
| Hernia | Hernia |
| Arterial Pressure | Arterial Pressure |
| Thoracentesis | Thoracentesis |
| Mild unsteadiness or fatigue | Mild unsteadiness or fatigue |
| ADP | Adenosine diphosphate |
| Pertussis vaccination | Pertussis vaccination |
| Progress of preexisting disease | Disease Progression |
| heart valve disease | Heart Valve Disease |
| negative | Negative |
| Urobilinogen measurement, urine | Urobilinogen measurement, urine |
| Balanced Crystalloid Solution | Balanced Salt Solution irrigation solution |

| | |
|---|---|
| High Sensitivity Troponin T Assay | High sensitivity cardiac troponin T measurement |
| Hernia of the abdominal wall | Hernia of abdominal wall |
| Thoracic Surgery | Thoracic surgery |
| Arterial blood pH | Blood arterial pH |
| Sensory impairment | Sensory disability |
| Current Procedural Terminology | Current Procedural Terminology Concept |
| ADP-Ribosyl Cyclase 1 | ADP-ribosyl Cyclase |
| Pipe | Pipe |
| Prostaglandin D2 | Prostaglandin D2 |
| Epithelial-Cast Measurement | Urine microscopy: epithelial casts |
| pancreatic cancer | Pancreatic cancer |
| colistin | colistin |
| Balanced diet | Balanced diet |
| Cardiac Output | Cardiac output |
| Arthritis | Arthritis |
| medical condition | Other medical condition |
| hemangioma | Hemangioma |
| Pneumococcal Conjugate Vaccine | Pneumococcal conjugate vaccine |
| Prostate-Specific Antigen | Prostate-Specific Antigen |
| sputum | Sputum |
| Epithelial Cell Count | Epithelial cell count |
| Not Applicable | Not applicable |
| polyarteritis nodosa | Polyarteritis nodosa |
| comment | Comment |
| Barbiturate screen | Barbiturate screening |
| Homocysteine | Homocysteine |
| Throat Carcinoma | Throat Cancer |
| Worse | Worse |
| Cyanosis | Cyanosis |
| hematoma | hematoma |
| active ingredient | Has active ingredient |
| Safety | Safety |
| Prostatic acid phosphatase | Prostatic acid phosphatase measurement |

| | |
|---|---|
| Other Route of Administration | Route of administration |
| Epoetin Alfa | Epoetin alfa |
| Acid phosphatase, non-prostatic fraction | Acid phosphatase, non-prostatic fraction |
| polymyositis | Polymyositis |
| complement C3 measurement | Complement C3 |
| Baricitinib | Baricitinib |
| Closed drainage of chest | Closed drainage of chest |
| stage date | Date |
| Ascites | Ascites |
| Thrombotic microangiopathy | Thrombotic microangiopathy |
| maximum ventilation pressure (Pmax) | Maximum airway pressure limit |
| High | High |
| How much did pain interfere with your activities at home? | How much did pain interfere with your day to day activities in past 7 days |
| Salicylates | Salicylates \| Bld-Ser-Plas |
| hematopoietic system disease | Disorder of hematopoietic system |
| Opportunistic infection | Opportunistic Infections |
| Protein C | Protein C |
| Erroneous | Erroneous |
| Activated partial thromboplastin time (aPTT) (highest) | Activated partial thromboplastin time |
| prostate cancer | Prostate cancer |
| complement C4 measurement | C4 complement assay |
| neurodegenerative disease | Neurodegenerative disorders |
| Klebsiella | Klebsiella |
| Cardiomegaly | Cardiomegaly |
| Ascites amylase | Ascitic fluid amylase level |
| Thyroglobulin | Thyroglobulin |
| High-Dependency Unit | Secure and high-dependency mental health care |
| Huntington's disease | Huntington's disease |
| antibiotic | Antibiotic |
| DNA Nucleotidylexotransferase | DNA nucleotidylexotransferase |
| Clopidogrel | Clopidogrel |

| | |
|---|---|
| hemoglobin distribution width | Hemoglobin distribution width |
| Sambucus | Sambucus |
| Pneumocystis Pneumonia | Pneumocystis carinii pneumonia |
| Protein to Creatinine Ratio Measurement | Protein/creatinine ratio measurement |
| Complementary medicine | Complementary Therapies |
| sarcoidosis | Sarcoidosis |
| neurologic neglect syndrome | Neurologic neglect syndrome |
| Lung Disorder | Disorder of lung |
| Klebsiella oxytoca | Klebsiella oxytoca |
| Cardiopulmonary Resuscitation | Cardiopulmonary resuscitation |
| Ascitic fluid protein measurement | Ascitic fluid protein measurement |
| Wound Discharge | Wound discharge |
| hemophilia | Hemophilia |
| Abatacept | Abatacept |
| Pneumocystis jirovecii | Pneumocystis jirovecii |
| Proteinuria | Proteinuria |
| Active substance (Coded Term) | Biologically active substance |
| Rigor (finding) | Rigor |
| scleroderma | Scleroderma |
| neuromyelitis optica | Neuromyelitis optica |
| Klebsiella pneumoniae | Klebsiella pneumoniae |
| Cardiovascular Manifestation | Cardiovascular finding |
| Thyroxine-Binding Globulin | Thyroxine-Binding Globulin |
| Wound drain | Wound drain |
| Home | Home |
| immunosuppressive agent | Immunosuppressive agent |
| hemorrhage | Hemorrhage |
| anatomical structure | Anatomical structure |
| Pneumonia due to coronavirus disease 2019 | Pneumonia due to coronavirus disease 2019 |
| Proteus mirabilis | Proteus mirabilis |
| Erythropoietin | Erythropoietin |
| Active substance (Modified Term) | Biologically active substance |
| skeletal system disease | Disease of Skeletal System |

| | |
|---|---|
| Acute respiratory distress syndrome | Acute respiratory distress syndrome |
| Klebsiella pneumoniae - 3MRGN | Klebsiella pneumoniae with multi-resistance 3MRGN |
| Aspergillus antigen index | Aspergillus antigen level |
| Homeopathy | Homeopathy |
| neuromuscular disease | Neuromuscular disease |
| Daratumumab | Daratumumab |
| Abdomen | Abdomen |
| Proteus vulgaris | Proteus vulgaris |
| completed | completed |
| Escherichia coli | Escherichia coli |
| stomach cancer | stomach cancer |
| Venous Thrombosis | Venous thrombosis |
| nitrates | Nitrates |
| Klebsiella pneumoniae - 4MRGN | Klebsiella pneumoniae with multi-resistance 4MRGN |
| basophil | Basophil |
| Crackles | Respiratory crackles |
| Care of body sites | Care of body sites |
| Admission time | Admission time |
| Aspergillus fumigatus | Aspergillus fumigatus |
| Horowitz index | Horowitz index |
| hepatic encephalopathy | Hepatic encephalopathy |
| Mindfulness Relaxation | Mindfulness based therapy |
| paresis | Paresis |
| Abdominal Aorta Thrombosis | Thrombosis of abdominal aorta |
| Portal Hypertension | Portal hypertension |
| Prothrombin | Prothrombin |
| Other dysphagia | Dysphagia |
| start date | Start date |
| stroke | Stroke |
| nitrendipine | Nitrendipine |
| opportunistic pathogen | Opportunistic infectious agent |
| Assessment | Assessment |

| | |
|---|---|
| Mineral Oil | Mineral oil |
| hepatitis C | Hepatitis C |
| Abdominal Ultrasound | Abdominal ultrasound |
| Postpartum | Postpartum |
| Proto-Oncogene Proteins c-kit | Proto-Oncogene Proteins c-kit |
| Fatal (qualifier value) | Fatal |
| systemic lupus erythematosus | Systemic lupus erythematosus |
| nitroglycerin | nitroglycerin |
| Laboratory | Laboratory |
| Cryptococcus | Cryptococcus |
| Carrier of other specified bacterial diseases | Carrier of other specified bacterial diseases |
| Mitoxantrone Hydrochloride | Mitoxantrone hydrochloride |
| hereditary ataxia | Neuropathy in association with hereditary ataxia |
| Scheduled | Scheduled |
| Abdominal compartment syndrome | Abdominal compartment syndrome |
| temporal arteritis | Temporal arteritis |
| Laboratory Procedure | Laboratory procedure |
| Current Smoker | Current smoker |
| Assessment Date | Assessment reference date |
| Yeast | Yeast |
| high-density lipoprotein cholesterol | HDL cholesterol |
| Schistocyte | Schistocyte |
| Date of Death | Date of death |
| Iloprost | Iloprost |
| infectious disease | Infectious Disease |
| Puncture of lung | Puncture of lung |
| start time | Start time |
| clinical trial | Clinical trial |
| Other delirium | Other delirium |
| Estimated glomerular filtration rate | Estimated glomerular filtration rate by laboratory calculation |
| Catheter-Related Infection | Catheter-Related Infections |
| medical device | Medical device |

| | |
|---|---|
| Patient Discharge | Patient Discharge |
| Timestamp Data Type | Timestamp |
| Controlled mandatory ventilation | Controlled mandatory ventilation |
| Moderate | moderate |
| Date of Evaluation | Date of last evaluation |
| transient cerebral ischemia | Transient cerebral ischemia |
| Ability to Go Up and Down Stairs at a Normal Pace | Are you able to go up and down stairs at a normal pace |
| viral infectious disease | Viral infectious disease |
| Renal insufficiency | Renal insufficiency |
| Providencia sp. | Providencia |
| Ex-smoker | Ex-smoker |
| non-alcoholic steatohepatitis | Nonalcoholic steatohepatitis |
| Bed scale | Bed scale |
| Catheter Device | Catheter, device |
| stomach carcinoma | Carcinoma of stomach |
| volume controlled ventilation | Volume controlled ventilation |
| Tissue Transglutaminase IgA Antibody Measurement | Tissue transglutaminase IgA measurement |
| medical procedure | Medical procedure |
| Screening for hypertension | Screening for hypertension |
| Blood Culture | Blood culture |
| Abciximab | Abciximab |
| ulcer | Ulcer |
| Ability to Run Errands and Shop | Are you able to run errands and shop |
| nonischemic cardiomyopathy | nonischemic cardiomyopathy |
| congenital disorder | Congenital disorder |
| Assist-control ventilation | Assist-control ventilation |
| Zip Code | Person One Address: Person One Address Zip Code |
| How Much Did Pain Interfere with Ability to Participate in Social Activities | How much did pain interfere with your ability to participate in social activities in past 7 days |
| Medical Research Council Breathlessness Score | Medical Research Council breathlessness scale score |

| | |
|---|---|
| Bronchoalveolar Lavage | Bronchoalveolar lavage |
| Resuscitation | Resuscitation |
| Pseudomonas | Pseudomonas |
| Arterial Oxygen Saturation | Arterial oxygen saturation |
| Other extracorporeal oxygenation or decarboxylation support | Extracorporeal Membrane Oxygenation or Extracorporeal Life Support Services and Procedures |
| Moderate dementia | Moderate dementia |
| norfloxacin | Norfloxacin |
| corticobasal syndrome | Corticobasal syndrome |
| uterine cancer | Uterine Cancer |
| Follow-Up | Follow-up |
| Assisted spontaneous breathing | Mechanically assisted spontaneous ventilation |
| How Much Did Pain Interfere with Day to Day Activity | How much did pain interfere with your day to day activities in past 7 days |
| Exertional dyspnea | Dyspnea on exertion |
| urine | Urine |
| hydrotherapy | Hydrotherapy |
| eptifibatide | eptifibatide |
| Retinopathy | Retinopathy |
| Pseudomonas aeruginosa | Pseudomonas aeruginosa |
| Discharged Alive | Patient discharged alive |
| Month and year of initial HIV diagnosis | Date of HIV diagnosis |
| normoblast | Normoblast |
| Belatacept | Belatacept |
| Cytology | Cytology |
| vaginal cancer | vaginal cancer |
| connective tissue disease | undifferentiated connective tissue disease |
| Tobramycin-random | Tobramycin^random |
| How Much Did Pain Interfere with Household Chores | How much did pain interfere with your household chores in past 7 days |
| Implant | Implant |
| Tirofiban | Tirofiban |
| Microbiology Specimen Test | Microbiology screening test |

| | |
|---|---|
| Glasgow Coma Score motor response subscore | Glasgow Coma Score motor response subscore |
| Rhabdomyolysis | Rhabdomyolysis |
| Pseudomonas aeruginosa - 3MRGN | Pseudomonas aeruginosa with multi-resistance 3MRGN |
| Alzheimer's disease | Alzheimer's disease |
| abducens nerve disease | Abducens nerve disorder |
| nose | Nose |
| Belimumab | Belimumab |
| consciousness | Consciousness |
| study group population size | Population size |
| Tomography - chest normal | Tomography - chest normal |
| How Often Feel Tired | How often did you feel tired in past 7 days |
| Expiration Date | Expiration date |
| hypertensive renal disease | Hypertensive renal disease |
| Sensitivity qualifier | Sensitivity |
| Abnormal flexion | Abnormal flexion |
| Pseudomonas aeruginosa - 4MRGN | Pseudomonas aeruginosa with multi-resistance 4MRGN |
| abnormal | Abnormal |
| adult-onset Still's disease | Adult-onset Still's disease |
| Benserazide and levodopa only product | Benserazide and levodopa only product |
| Cytomegalovirus | Cytomegalovirus |
| Too Breathless to Leave the House or Breathless After Undressing | Are you too breathless to leave the house or breathless on dressing or undressing |
| sufentanil | Sufentanil |
| Facial paralysis | Facial paralysis (machine translation) |
| Oseltamivir | Oseltamivir |
| hypertrophic cardiomyopathy | Hypertrophic cardiomyopathy |
| Sepsis due to other specified staphylococcus | Sepsis due to other specified staphylococcus |
| Abnormal reflex | Abnormal reflex [D] |
| Pseudomonas sp. | Pseudomonas |
| Pregnancy Outcome | Pregnancy outcome |
| Other macrolide | Macrolide |
| Amylase:CCnc:Pt:Body fld:Qn | Amylase measurement, body fluid |

| | |
|---|---|
| acetone | Acetone |
| Moraxella catarrhalis | Moraxella catarrhalis |
| Sjogren's syndrome | Sjogren's Syndrome |
| calcium acetate / magnesium carbonate Oral Tablet | calcium acetate / magnesium carbonate Oral Tablet [Magnebind-200] |
| Benzodiazepines screen method | Benzodiazepines screen method |
| Cellular Cast Measurement | Cellular cast |
| contact person | Contact person |
| sulfasalazine | Sulfasalazine |
| Topical medication | Application of topical medication |
| pulmonary lobar consolidation | Lung consolidation |
| Cognitive impairment | Cognitive impairment |
| Child Development | Child development |
| Amylase:CCnc:Pt:Plr fld:Qn | Amylase measurement, pleural fluid |
| Morganella morganii | Morganella morganii |
| nursing home | Nursing home |
| Takayasu's arteritis | Takayasu Arteritis |
| Beta-2-Microglobulin | Beta-2-Microglobulin |
| Dyspnea (finding) | Dyspnea |
| contact specification - phone | contact phone number |
| sulfonylurea | Sulfonylurea |
| Respiratory physiotherapy | Respiratory physiotherapy |
| Extravascular lung water index | Measurement of extravascular lung water |
| Serratia marcescens | Serratia marcescens |
| Date of admission to this facility | Date of admission |
| immunization date | immunization date |
| Abnormality of vision | Disorder of vision |
| Psychosomatic Medicine | Psychosomatic Medicine |
| acupuncture | Acupuncture |
| Other neurological conditions | Other neurological conditions |
| nystagmus | Nystagmus |
| Respiratory disorder | RESPIRATORY DISORDER |
| Beta-Hemolytic Streptococcus | Beta-hemolytic streptococcus |
| Central Venous Access Catheter | Central venous catheter |

| | |
|---|---|
| Productive cough | Productive cough |
| Total | Total |
| Astragalus | Astragalus |
| surgery | Surgery |
| Thorax drainage | Closed drainage of thorax |
| Eye care | Eye care |
| About The Same | About the same |
| blood | Blood |
| Psychotherapy | Psychotherapy |
| acute | Acute |
| nystatin | nystatin |
| Law Enforcement | Discharged / transferred to Court / Law Enforcement |
| pericardial effusion | Pericardial effusion |
| Better | Healthcare Coverage: Better |
| Central venous sinus thrombosis | Cerebral venous sinus thrombosis |
| Ageusia | Loss of taste |
| core body temperature | Core body temperature |
| blood cell measurement | Blood cell analysis |
| Tracheal Hemorrhage | Tracheal hemorrhage |
| surgical history date | Surgical operation date |
| FDA National Drug Code | FDA product label Indexing - national clinical trial number |
| Referral | Referral |
| Serum Globulin | Immune Serum Globulin |
| Absolute Blood Lymphocyte Count | ABSOLUTE LYMPHOCYTE COUNT |
| Pulmonary Consolidation | Lung consolidation |
| acute lymphoblastic leukemia | Acute lymphoblastic leukemia |
| Arterial blood lactate level | Arterial blood lactate level |
| Other specified anemias | Other specified anemias |
| Antigens, CD13 | CD13 |
| obstructive nephropathy | Obstructive nephropathy |
| Left Ventricular Ejection Fraction | Left ventricular ejection fraction |
| Other respiratory disorders | Other respiratory disorders |

| | |
|---|---|
| Certolizumab | Certolizumab |
| cortisol | Cortisol |
| susceptible antimicrobial phenotype | Antimicrobial susceptible bacteria |
| Deep Vein Thrombosis | Deep vein thrombosis |
| FHS CVD 10-Year Risk Score - Risk Score | Framingham coronary heart disease 10 year risk score |
| Serum Viscosity | Viscosity \| Serum \| Specimen information |
| Acanthocyte | Acanthocyte |
| Septation | Septation - action |
| acute myeloid leukemia | Acute myeloid leukemia |
| Other specified diabetes mellitus | E13 Other specified diabetes mellitus |
| Moxonidine | Moxonidine |
| Left Ventricular Failure | Left ventricular failure |
| Bicarbonate in other fluid | Bicarbonate in sample |
| Miscellaneous | Miscellaneous |
| cyclophosphamide | Cyclophosphamide |
| swallowing | Swallowing |
| Ataxia | Ataxia |
| Tracheobronchial stent | Tracheobronchial stent |
| Howell-Jolly bodies | Howell-Jolly bodies |
| Favipiravir | Favipiravir |
| Serum creatinine | Serum creatinine level |
| Accident and Emergency department | Accident and Emergency department |
| Pulmonary hemorrhage | Pulmonary hemorrhage |
| ongoing | Ongoing |
| Apt test (hemoglobin), stool | Apt test (hemoglobin), stool |
| hypoglycemic drug | Hypoglycemic agent |
| ophthalmoplegia | Ophthalmoplegia |
| Left Ventricular Mass | Left ventricular mass |
| Bilateral | Bilateral |
| cyanocobalamin (b12) | Cyanocobalamin (Vitamin B-12) |
| Change | Change |
| systemic arterial blood pressure | Systemic arterial pressure |

| | |
|---|---|
| Cytopenia | Cytopenia |
| Dialysis | Dialysis |
| Human Chorionic Gonadotropin | Human chorionic gonadotropin |
| Factor VIII Inhibitor Measurement | Factor VIII inhibitor measurement |
| implant type code | Type of implant |
| Servo Device | Servo |
| Acid Phosphatase | Acid Phosphatase |
| Respiration | Respiration |
| pulse | Pulse |
| kidney disease | Kidney Disease |
| Other staphylococcus as the cause of diseases classified to other chapters | Other staphylococcus as the cause of diseases classified to other chapters |
| Barbiturates:MCnc:Pt:Urine:Qn:Screen | Barbiturates screen method \| Urine \| Drug toxicology |
| oral candidiasis | Oral candidiasis |
| Multiple Organ Failure | Multiple Organ Failure |
| Dry cough (finding) | Dry cough |
| end-tidal partial pressure of carbon dioxide (PETCO2) | Arterial partial pressure of carbon dioxide |
| Change in medication | Change of medication |
| Healthcare Facility | Healthcare facility |
| dabigatran | dabigatran |
| systemic inflammatory response syndrome | Systemic inflammatory response syndrome |
| Discharge from intensive care service | Discharge from intensive care service |
| Human Parainfluenza Virus 1 | Human parainfluenza virus 1 |
| Immunoglobulins, Intravenous | Immunoglobulins, Intravenous |
| Factor XI | Factor XI |
| Severe Cognitive Impairments | Severe cognitive impairment |
| Acinetobacter baumannii | Acinetobacter baumannii |
| Spontaneous respiration | Spontaneous respiration |
| Other symptoms and signs involving cognitive functions and awareness | Other symptoms and signs involving cognitive functions and awareness |
| pancreatitis | Pancreatitis |
| Behcet's syndrome | Behcet's syndrome |
| oral cavity carcinoma | Carcinoma in situ of oral cavity |

| Billing | Billing |
|---|---|
| systemic scleroderma | Scleroderma, Systemic |
| Human Parainfluenza Virus 2 | Human parainfluenza virus 2 |
| Factor XII | Factor XII |
| Severe dementia | Severe dementia |
| Acinetobacter baumanii - 3MRGN | Acinetobacter baumannii group with multi-resistance 3MRGN |
| Nein | No |
| alcoholic fatty liver disease | Alcoholic fatty liver disease |
| Pulse index Contour Continuous Cardiac Output | Monitoring of cardiac output using pulse contour analysis |
| Other systemically active antifungals | Antifungals |
| Benzodiazepines:MCnc:Pt:Urine:Qn:Screen | Benzodiazepines panel - Urine |
| oriented | Oriented |
| Mycoplasma pneumoniae | Mycoplasma pneumoniae |
| transplantation | Transplantation |
| Other | Other |
| Transfer from a different hospital | Transfer from a different hospital |
| Atrial fibrillation 10Y risk | Atrial fibrillation 10Y risk |
| Disseminated Intravascular Coagulation | Disseminated intravascular coagulation |
| Human Parainfluenza Virus 3 | Human parainfluenza virus 3 |
| Date of influenza vaccination | Date of influenza vaccination |
| Acinetobacter baumanii - 4MRGN | Acinetobacter baumannii group with multi-resistance 4MRGN |
| alcoholic liver cirrhosis | alcoholic liver disease / alcoholic cirrhosis |
| Purulent Discharge | Purulent discharge |
| Outpatient | Outpatient |
| protease inhibitor | Protease inhibitor |
| oropharyngeal swab specimen | Oropharyngeal swab |
| Myeloid Cell Surface Antigen CD33 | CD33 Ag |
| Left ventricular noncompaction cardiomyopathy | Left ventricular myocardial noncompaction cardiomyopathy |
| Oxygen supplementation | Oxygen therapy |
| teicoplanin | teicoplanin |
| Transfer from another type of Healthcare | Transfer from another type of Healthcare |

| | |
|---|---|
| facility NOS | facility NOS |
| Human Parainfluenza Virus 4 | Human parainfluenza virus 4 |
| Infection following a procedure, not elsewhere classified | Infection following a procedure, not elsewhere classified |
| Gait | Gait |
| Short of Breath When Hurrying on Level Ground or Walking Up a Slight Hill | Are you troubled by shortness of breath when hurrying on level ground or walking up a slight hill |
| Acinetobacter sp. | Acinetobacter |
| aldosterone antagonist | Aldosterone antagonist |
| Pyramidal symptoms | Pyramidal sign |
| Oval fat body | Oval fat body |
| revascularization | Revascularization - action |
| osmotic concentration | Osmolarity |
| N-acetylprocainamide | N-acetylprocainamide |
| Left without being seen | Left without being seen |
| telephone | Telephone |
| Human bocavirus | Human bocavirus |
| Shunt Device | Shunt |
| alfacalcidol | Alfacalcidol |
| Bicarbonate:SCnc:Pt:Plr fld:Qn | Bicarbonate \| Pleural fluid |
| ritonavir | Ritonavir |
| Legionella longbeachae | Legionella longbeachae |
| Birth Weight | Birth weight |
| Chemotherapy | Chemotherapy |
| airway management | Airway management |
| teriflunomide | Teriflunomide |
| Transferrin | Transferrin |
| Dysphagia | Dysphagia |
| Human coronavirus 229E | Human coronavirus 229E |
| Falls | Falls |
| Sialic Acid Binding Ig-like Lectin 1 | Sialic Acid Binding Ig-like Lectin 1 |
| inhalation | Inhalation |
| Active | Active |

| | |
|---|---|
| alpha-fetoprotein | Alpha-fetoprotein (AFP) |
| Quick SOFA | qSOFA - quick Sequential Organ Failure Assessment |
| Bicarbonate:SCnc:Pt:Urine:Qn | Urine bicarbonate level |
| sedative | Sedative |
| Inspiratory/expiratory ratio | Inspiratory/expiratory ratio |
| NUTREN PULMONARY | NUTREN PULMONARY |
| Legionella pneumophila | Legionella pneumophila |
| Birth Year | Birth year |
| tetracycline | Tetracycline |
| Human coronavirus HKU1 | Human coronavirus HKU1 |
| Sickle Cell Preparation | Sickle cell identification |
| Active disease diagnosis | Active disease diagnosis |
| amyotrophic lateral sclerosis | Amyotrophic lateral sclerosis |
| Herzinsuffizienz | Heart failure, unspecified |
| signing an informed consent form | Informed consent obtained |
| outpatient encounter | Outpatient Visit |
| Chest X-Ray | Chest X-ray |
| High-Flow Nasal Cannula Oxygen Therapy | Oxygen administration by nasal cannula |
| third cranial nerve disease | Third cranial nerve disease |
| Enoxaparin Sodium | Enoxaparin sodium |
| Human coronavirus NL63 | Human coronavirus NL63 |
| Tollwut-Immunglobulin | Rabies immunoglobulin |
| ambulatory - llimitation of activities | Ambulatory - Limitation of activities |
| Desirudin | Desirudin |
| Röteln-Immunglobulin | Rubella immunoglobulin |
| hospitalised/mild disease - no oxygen therapy | Hospitalized - mild disease, no oxygen therapy |
| hospitalized/ severe disease - intubation and mechanical ventilation | Hospitalized - severe disease, intubation and mechanical ventilation |
| Leukocyte alkaline phosphatase | Leukocyte alkaline phosphatase |
| Argatroban | Argatroban |
| Human respiratory syncytial virus A | Human respiratory syncytial virus A |
| hospitalized/mild disease - oxygen by mask | Hospitalized - mild disease, oxygen by mask |

| | |
|---|---|
| or nasal prongs | or nasal prongs |
| Melagatran | Melagatran |
| Human respiratory syncytial virus B | Human respiratory syncytial virus B |
| hospitalized/ severe disease -ventilation and additional organ support - pressors, RRT, ECMO | Hospitalized - severe disease, ventilation and additional organ support - pressors, RRT, ECMO |
| Diphtherie-Immunglobulin | Diphtheria immunoglobulin |
| exhaustion | Exhaustion |
| FSME-Immunglobulin | FSME-Bulin |
| Additional information Other symptoms | Other symptoms |
| Pertussis-Immunglobulin | Bordetella pertussis IgG |
| Masern-Immunglobulin | Measles immunoglobulin |
| Palliative discharge | Discharge from palliative care service |
| Mumps-Immunglobulin | Mumps immunoglobulin |
| Final | Final |
| Eruption of skin (disorder) | Eruption of skin present |
| Palivizumab | Palivizumab |
| Does the patient suffer from diabetes? | Do you suffer from diabetes |
| Betrixaban | Betrixaban |
| Type of ECMO therapy | ECMO |
| Unable to walk (finding) | Unable to walk |
| Raxibacumab | Raxibacumab |
| Disturbance of consciousness (finding) | Disturbance of consciousness |
| Bezlotoxumab | Bezlotoxumab |
| Citrobacter sp. | Citrobacter |
| INACTIVE | Inactive |
| Obiltoxaximab | Obiltoxaximab |
| Enalapril | Enalapril |
| Kombinationen | Combinations |
| Decreasing | Decreasing |
| Lisinopril | Lisinopril |
| Andere Immunglobuline | Other immunoglobulins |
| Andere Analgetika und Antipyretika | Other analgesics and antipyretics |
| Perindopril | Perindopril |

| | |
|---|---|
| Nebacumab | Nebacumab |
| Rimazolium | Rimazolium |
| Ramipril | Ramipril |
| Deleted | Deleted |
| Glafenin | Glafenine |
| Quinapril | Quinapril |
| Clear | Clear |
| Type of ventilation | Ventilation |
| Floctafenin | floctafenine |
| Benazepril | Benazepril |
| Cartilage tissue | Cartilage tissue |
| 180D | 180 days |
| Viminol | viminol |
| Cilazapril | Cilazapril |
| Four quadrants | Four quadrants |
| Nefopam | Nefopam |
| New long-term oxygen therapy | Long-term oxygen therapy |
| Constipation, unspecified | Constipation, unspecified |
| Fosinopril | Fosinopril |
| Immune status information | Immune status |
| Underlying medication | Medication |
| Bone (tissue) structure (body structure) | Bone (tissue) structure |
| Dialysis finding | Dialysis finding |
| History of cerebrovascular accident without residual deficits | History of cerebrovascular accident without residual deficits |
| Flupirtin | flupirtine |
| Specify other symptoms | Other symptoms |
| Trandolapril | Trandolapril |
| Delirium due to known physiological condition | Delirium due to known physiological condition |
| Dependence on renal dialysis | Dependence on renal dialysis |
| Ziconotid | ziconotide |
| Chronic nervous system disorder (disorder) | Chronic nervous system disorder |
| Spirapril | Spirapril |

| | |
|---|---|
| Interferon gamma | Interferon gamma |
| Unspecified mental disorder due to known physiological condition | Unspecified mental disorder due to known physiological condition |
| Does the patient take regular medication? | Does the patient take any medications |
| Infectious disease of lung | Infectious disease of lung |
| Methoxyfluran | methoxyflurane |
| Delapril | Delapril |
| Interferon alfa-2a | Interferon alfa-2a |
| Pre-renal acute kidney injury (disorder) | Pre-renal acute kidney injury |
| Cannabinoide | Cannabinoids |
| Moexipril | Moexipril |
| Interferon alfa-2b | Interferon alfa-2b |
| Epilepsy and recurrent seizures | Epilepsy and recurrent seizures |
| Sehne | Tendon |
| Interferon alfa-n1 | Interferon alfa-n1 |
| turbid | Turbid |
| Aconitum | Aconitum |
| Complete/COMPLETE | Complete |
| Zofenopril | Zofenopril |
| Patient referral | Patient referral |
| Interferon beta-1a | Interferon beta-1a |
| Cerebral infarction due to unspecified occlusion or stenosis of unspecified cerebral artery | Cerebral infarction due to unspecified occlusion or stenosis of unspecified cerebral artery |
| Verschiedene | Miscellaneous |
| Imidapril | Imidapril |
| Interferon beta-1b | Interferon beta-1b |
| Cerebral infarction, unspecified | Cerebral infarction, unspecified |
| New (intermitt.) invasive ventilation | Invasive ventilation |
| ACE-HEMMER, KOMBINATIONEN | ACE INHIBITORS, COMBINATIONS |
| Interferon alfacon-1 | Interferon alfacon-1 |
| Acute bronchitis due to other specified organisms | Acute bronchitis due to other specified organisms |
| New (intermitt.) non-invasive ventilation | Non-invasive ventilation |

| Reason for Visit | Reason for visit |
|---|---|
| In general, how exhausted did you feel? | How exhausted were you on average in past 7 days |
| ACE-Hemmer und Diuretika | ACE inhibitors and diuretics |
| Peginterferon alfa-2b | Peginterferon alfa-2b |
| Unspecified acute lower respiratory infection | unspecified acute lower respiratory infection |
| Aspergillus sp. | Aspergillus |
| Blauer Eisenhut | Monkshood |
| Increasing | Increasing |
| Magnitude of anxiety in the patient(s) | Level of anxiety |
| Captopril und Diuretika | captopril and diuretics; systemic |
| Peginterferon alfa-2a | Peginterferon alfa-2a |
| Bronchitis, not specified as acute or chronic | Bronchitis, not specified as acute or chronic |
| Dialysis access points | Dialysis access |
| Enalapril und Diuretika | enalapril and diuretics; systemic |
| Virus found | Virus identified |
| Albinterferon alfa-2b | albinterferon alfa-2b; systemic |
| Respiratory failure, not elsewhere classified | Respiratory failure, not elsewhere classified |
| Breastfeeding stopped | Breastfeeding stopped |
| Peginterferon beta-1a | Peginterferon beta-1a |
| Multisystem inflammatory syndrome | Multisystem inflammatory syndrome |
| < \|Thrombosis (disorder)\| | Thrombosis |
| Perindopril und Diuretika | perindopril and diuretics; systemic |
| History of cerebrovascular accident with residual deficit | History of cerebrovascular accident with residual deficit |
| biological attribute | Biological Factors |
| Kortikosteroide | Corticosteroids |
| Other amnesia | Other amnesia |
| Ropeginterferon alfa-2b | Ropeginterferon alfa-2b |
| Other complications due to prostheses, implants, or grafts in genitourinary tract | Other specified complications of genitourinary prosthetic devices, implants and grafts |
| Epinephrin und andere Mittel bei obstruktiven Atemwegserkrankungen | epinephrine and other drugs for obstructive airway diseases; inhalant |
| Quinapril und Diuretika | quinapril and diuretics; systemic |

| | |
|---|---|
| Altered mental status, unspecified | Altered mental status, unspecified |
| Interferon gamma-1b | Interferon gamma-1b |
| Isoprenalin und andere Mittel bei obstruktiven Atemwegserkrankungen | isoprenaline and other drugs for obstructive airway diseases; inhalant |
| Epidemic vertigo | Epidemic vertigo |
| Peginterferon alfa-2b, Kombinationen | peginterferon alfa-2b, combinations; parenteral |
| Long-term dependence on dialysis in renal failure | Dependence on renal dialysis |
| Urine appearance | Urine appearance |
| Malaise (finding) | Malaise |
| Unspecified convulsions | Unspecified convulsions |
| Peginterferon alfa-2a, Kombinationen | peginterferon alfa-2b, combinations; parenteral |
| Pain in throat (finding) | Pain in throat |
| Symptoms and signs specifically associated with systemic inflammation and infection | Symptoms and signs specifically associated with systemic inflammation and infection |
| Skin ulcer (disorder) | Skin ulcer |
| Involvement | Involvement |
| Severe sepsis | Severe sepsis |
| M. Parkinson | Parkinson disease |
| Formoterol und Budesonid | formoterol and budesonide; inhalant |
| Normal flexion | Normal flexion |
| Information on viral load | Viral load |
| COVID-19 | COVID-19 |
| Formoterol und Beclometason | formoterol and beclometasone; inhalant |
| Germ findings | Germ |
| Captopril und Hydrochlorothiazid | Captopril and Hydrochlorothiazide |
| C1 | C1 |
| Afelimomab | [U] afelimomab |
| Vilanterol und Fluticasonfuroat | vilanterol and fluticasone furoate; inhalant |
| C2 | C2 |
| Lisinopril und Hydrochlorothiazid | LISINOPRIL AND HYDROCHLOROTHIAZIDE - lisinopril and hydrochlorothiazide tablet |
| BCG | BCG |

| | |
|---|---|
| Formoterol und Fluticason | formoterol and fluticasone; inhalant |
| C3 | C3 |
| Glycopeptide intermediate Staphylococcus aureus | Glycopeptide intermediate Staphylococcus aureus |
| Certolizumab pegol | Certolizumab pegol |
| Quinapril und Hydrochlorothiazid | QUINAPRIL HCL AND HYDROCHLOROTHIAZIDE - quinapril hcl and hydrochlorothiazide tablet |
| Salbutamol und Beclometason | salbutamol and beclometasone; inhalant |
| Nasal discharge (finding) | Nasal discharge |
| If other, please specify | Other, specify |
| Personal history of infectious and parasitic diseases | Personal history of infectious and parasitic diseases |
| Fenoterol und Ipratropiumbromid | fenoterol and ipratropium bromide; inhalant |
| Fosinopril und Hydrochlorothiazid | Fosinopril and hydrochlorothiazide product |
| Current some day smoker | Current some day smoker |
| Daclizumab | Daclizumab |
| Reason for discharge | Reason for discharge |
| Salbutamol und Ipratropiumbromid | salbutamol and ipratropium bromide; inhalant |
| vancomycin intermediate Staphylococcus aureus | Vancomycin intermediate Staphylococcus aureus |
| Not indicated | Not indicated |
| Color of sputum | Color of sputum |
| Vilanterol und Umeclidiniumbromid | Umeclidinium bromide + Vilanterol |
| Zofenopril und Hydrochlorothiazid | Hydrochlorothiazide / zofenopril Oral Tablet |
| Smoker, current status unknown | Smoker, current status unknown |
| Discharge | Discharge |
| Psychose | Psychoses |
| Indacaterol und Glycopyrroniumbromid | Indacaterol + Glycopyrronium bromide |
| Perindopril und Indapamid | Perindopril And Indapamide |
| Unbekannt | Unknown |
| Rilonacept | Rilonacept |
| Formoterol und Aclidiniumbromid | Aclidinium bromide + Formoterol |
| Ramipril und Piretanid | piretanide / Ramipril |

| | |
|---|---|
| Heavy tobacco smoker | Heavy tobacco smoker |
| I found it hard to pay attention to anything other than my anxiety | I found it hard to focus on anything other than my anxiety in past 7 days |
| Olodaterol und Tiotropiumbromid | olodaterol and tiotropium bromide; inhalant |
| Light tobacco smoker | Light tobacco smoker |
| Insulin Drip | Insulin Drip |
| Pleural effusion, not elsewhere classified | Pleural effusion, not elsewhere classified |
| US | Please double check |
| Formoterol und Glycopyrroniumbromid | formoterol and glycopyrronium bromide; inhalant |
| Enalapril und Lercanidipin | Enalapril / lercanidipine Oral Tablet |
| Briakinumab | briakinumab; systemic |
| Fatigue/exhaustion/excessive tiredness | Fatigue |
| Vilanterol, Umeclidiniumbromid und Fluticasonfuroat | vilanterol, umeclidinium bromide and fluticasone furoate; inhalant |
| Lisinopril und Amlodipin | lisinopril and amlodipine; oral |
| Risankizumab | Risankizumab |
| I find it hard to do my usual work (including work at home) | I have trouble doing all of my usual work (include work at home) |
| Formoterol, Glycopyrroniumbromid und Beclometason | formoterol, glycopyrronium bromide and beclometasone; inhalant |
| Chronic hematological disease | Chronic disease of hematopoietic system |
| Perindopril und Amlodipin | Amlodipine / Perindopril |
| Insulin pump | Insulin pump |
| Pneumococcal infectious disease | Pneumococcal infectious disease |
| Ramipril und Felodipin | FELODIPINE / RAMIPRIL |
| Siltuximab | Siltuximab |
| Ganciclovir | Ganciclovir |
| Enalapril und Nitrendipin | Enalapril / Nitrendipine Oral Tablet |
| Brodalumab | Brodalumab |
| Cornea | Cornea |
| ambulatory - no limitation of activities | Ambulatory - No limitation of activities |
| Ramipril und Amlodipin | ramipril and amlodipine; oral |
| CORTICOSTEROIDE | Corticosteroid |
| Imidazole/Triazole in Kombination mit | imidazoles/triazoles in combination with |

| | |
|---|---|
| Corticosteroiden | corticosteroids; topical |
| Sirukumab | sirukumab; systemic |
| Trandolapril und Verapamil | Trandolapril + Verapamil |
| Confused | Confused |
| Guselkumab | Guselkumab |
| Dexamethason | Dexamethason |
| Delapril und Manidipin | delapril and manidipine; oral |
| Tildrakizumab | Tildrakizumab |
| Numerous | Numerous |
| Betamethason | Betamethason |
| ACE-Hemmer, andere Kombinationen | ACE inhibitors, other combinations |
| Aspergillus antigen in BAL | Aspergillus sp \| Bronchoalveolar lavage |
| Fluocinolonacetonid | Fluocinolone Acetonide |
| Perindopril, Amlodipin und Indapamid | perindopril, amlodipine and indapamide; systemic |
| Ciclosporin | Ciclosporin |
| Entire heart valve (body structure) | Entire heart valve |
| HMO REFERRAL | HMO Referral |
| Perindopril und Bisoprolol | perindopril and bisoprolol; systemic |
| Drug not available - out of stock | Drug not available - out of stock |
| Ramipril, Amlodipin und Hydrochlorothiazid | ramipril, amlodipine and hydrochlorothiazide; oral |
| Voclosporin | voclosporin |
| Perindopril, Bisoprolol und Amlodipin | perindopril, bisoprolol and amlodipine; systemic |
| Obey commands | Obeys commands |
| Phenylbutazon und Corticosteroide | phenylbutazone and corticosteroids; systemic |
| Haemophilus sp. | Haemophilus species |
| Has the patient already smoked at least one cigarette a day, every day for at least one month? | Has there ever been a period in your life when you smoked cigarettes every day for at least 30 days |
| Dipyrocetyl und Corticosteroide | dipyrocetyl and corticosteroids |
| Acetylsalicylsäure und Corticosteroide | acetylsalicylic acid and corticosteroids; systemic |
| Research | Research |

| | |
|---|---|
| Anemia associated with other chronic diseases classified elsewhere | Anemia in other chronic diseases classified elsewhere |
| Antibiotika und Corticosteroide | Antibiotics and corticosteroids |
| Covid-19 | COVID-19 |
| Resource | RESOURCE |
| Systemic lupus erythematosus treatment | Systemic lupus erythematosus |
| Antiseptika und Corticosteroide | Antiseptics and corticosteroids |
| Ear care | Ear care |
| Pressure support ventilator | Pressure support ventilator |
| Fettleber | Fatty liver |
| Colchicin | Colchicine |
| Community Acquired Pneumonia therapy | Community Acquired Pneumonia |
| Salicylsäure und Derivate | Salicylic acid and salicylic acid derivative |
| Aloxiprin | Aloxiprin |
| Pain (finding) | Pain |
| Cerebral meninges | Cerebral meninges |
| Cholinsalicylat | Choline salicylate |
| Can you sit on the edge of the bed | Are you able to sit on the edge of a bed |
| Mineralocorticoide | Mineralocorticoids |
| Natriumsalicylat | Sodium salicylate |
| Can you squat down and stand up again | Are you able to squat and get up |
| Salicylamid | salicylamide |
| Zinksulfat | Zinc sulfate |
| Type of measurement | Measurement type |
| Can you turn over in bed | Ability to roll over in bed |
| Fludrocortison | Fludrocortison |
| Salsalat | salsalate |
| Zinkgluconat | Zinkgluconat |
| Desoxycorton | Desoxycortone |
| Ethenzamid | ethenzamide |
| Zinkprotein-Komplex | zinc protein complex; systemic |
| B2 | B2 |
| Morpholinsalicylat | morpholine salicylate |
| Zinkhydrogenaspartat | zinc aspartate |

| | |
|---|---|
| Does your medical condition restrict you from taking a shower? | Does your health now limit you in taking a shower |
| Dipyrocetyl | dipyrocetyl; oral |
| Zinkorotat | Zinkorotat |
| Benorilat | benorilate |
| < \|Disorder of liver (disorder)\| | Disorder of Liver |
| Fluocortolon | Fluocortolone |
| Chronic viral hepatitis | Chronic viral hepatitis |
| Diflunisal | Diflunisal |
| Heparingruppe | Heparin group |
| Autoimmune Lebererkrankungen | Autoimmune liver disease |
| B1 | B1 |
| Systolic blood pressure present on admission? | Systolic blood pressure on admission |
| Paramethason | Paramethasone |
| Antithrombin III, Antithrombin alfa | Antithrombin alfa |
| Lysin-Acetylsalicylat | Lysine acetylsalicylate |
| Enoxaparin | Enoxaparin |
| Guacetisal | Guacetisal |
| B3 | B3 |
| Insulin treated diabetes type 2 | Insulin treated type 2 diabetes mellitus |
| Prednison | Prednison |
| Low-molecular-weight heparin | Heparin, Low-Molecular-Weight |
| Carbasalat calcium | Carbasalate Calcium |
| Triamcinolon | Triamcinolon |
| Imidazolsalicylat | imidazole salicylate |
| Cholin-Magnesium-Tris-Salicylat | Choline magnesium trisalicylate |
| Enterobacter sp. | Enterobacter |
| Cortison | Cortison |
| Danaparoid | Danaparoid |
| Feeling feverish (finding) | Feeling feverish |
| Isolated | Isolated |
| Prednyliden | prednylidene |
| Acetylsalicylsäure, Kombinationen exkl. | acetylsalicylic acid, combinations excl. |

| | |
|---|---|
| Psycholeptika | psycholeptics; systemic |
| Rimexolon | Rimexolone |
| A1 | A1 |
| Salicylamid, Kombinationen exkl. Psycholeptika | salicylamide, combinations excl. psycholeptics; systemic |
| Deflazacort | Deflazacort |
| Bemiparin | Bemiparin |
| A2 | A2 |
| Enterococcus sp. | Enterococcus sp |
| Cloprednol | Cloprednol |
| Certoparin | Certoparin |
| A3 | A3 |
| Dipyrocetyl, Kombinationen exkl. Psycholeptika | dipyrocetyl, combinations excl. psycholeptics |
| Cortivazol | Cortivazol |
| Heparin, Kombinationen | heparin, combinations; systemic |
| Professional | Professional |
| Carbasalat calcium, Kombinationen exkl. Psycholeptika | carbasalate calcium combinations excl. psycholeptics; oral, rectal |
| Indrawing of ribs during respiration (finding) | Indrawing of ribs during respiration |
| Acetylsalicylsäure, Kombinationen mit Psycholeptika | acetylsalicylic acid, combinations with psycholeptics; systemic |
| Antiplatelet drug | antiplatelet drug |
| Salicylamid, Kombinationen mit Psycholeptika | salicylamide, combinations with psycholeptics; systemic |
| Working | Working |
| Ventilation mode | Ventilation mode |
| Currently breastfeeding | Currently breastfeeding |
| Ditazol | ditazole |
| Other Reason | Other reason |
| Ethenzamid, Kombinationen mit Psycholeptika | ethenzamide, combinations with psycholeptics; oral, rectal, topical |
| Cloricromen | cloricromen |
| Dipyrocetyl, Kombinationen mit Psycholeptika | dipyrocetyl, combinations with psycholeptics |
| Picotamid | picotamide |

| | |
|---|---|
| Fever (finding) | Fever |
| Other allergy | Other allergy |
| Phenazon | Phenazone |
| Is data entry for this section finished? | Data entry of non-scannable items |
| Methylprednisolon, Kombinationen | methylprednisolone, combinations; systemic |
| Ticlopidin | Ticlopidin |
| Metamizol-Natrium | Metamizole sodium |
| Other cause of death | Other cause of death |
| Aminophenazon | Aminophenazone |
| Propyphenazon | Propyphenazon |
| Triamcinolon, Kombinationen | triamcinolone, combinations; topical |
| Nifenazon | nifenazone |
| Acute respiratory failure, unspecified whether with hypoxia or hypercapnia | Acute respiratory failure, unspecified whether with hypoxia or hypercapnia |
| Klebsiella sp. | Klebsiella |
| Betamethason, Kombinationen | betamethasone, combinations; topical |
| Epoprostenol | Epoprostenol |
| Phenazonsalicylat | Antipyrine Salicylate |
| Time of event | Time of event |
| Indobufen | Indobufen |
| Has the patient knowingly had contact with a probable or confirmed COVID-19 patient in the 14 days prior to the onset of his/her symptoms? | History of Close contact with confirmed COVID-19 case patient in the 14 days prior to illness onset |
| Phenazon, Kombinationen exkl. Psycholeptika | phenazone, combinations with psycholeptics; systemic |
| Metamizol-Natrium, Kombinationen exkl. Psycholeptika | metamizole sodium, combinations excl. psycholeptics; systemic |
| Invasive ventilation (endotracheal) | Invasive ventilation |
| Sulfinpyrazon | Sulfinpyrazone |
| Aminophenazon, Kombinationen exkl. Psycholeptika | aminophenazone, combinations excl. psycholeptics; systemic |
| Anticorticosteroide | Anticorticosteroids |
| Propyphenazon, Kombinationen exkl. Psycholeptika | propyphenazone, combinations with psycholeptics; oral, otic, rectal, topical |
| Currently | Currently |

| | |
|---|---|
| Trilostan | trilostane |
| hospitalized severe disease - non-invasive ventilation or high-flow oxygen | Hospitalized - severe disease, non-invasive ventilation or high-flow oxygen |
| Ketoconazol | ketoconazol |
| Phenazon, Kombinationen mit Psycholeptika | phenazone, combinations with psycholeptics; systemic |
| Self pay | Self pay |
| Metamizol-Natrium, Kombinationen mit Psycholeptika | metamizole sodium, combinations with psycholeptics; systemic |
| Atazanavir | Atazanavir |
| Aminophenazon, Kombinationen mit Psycholeptika | aminophenazone, combinations with psycholeptics; systemic |
| Triflusal | Triflusal |
| Propyphenazon, Kombinationen mit Psycholeptika | propyphenazone, combinations with psycholeptics; oral, otic, rectal, topical |
| Beraprost | Beraprost |
| How many cigarettes does the patient smoke on average per day | How many cigarettes do you smoke per day now |
| Ribavirin | Ribavirin |
| Extension | Extension |
| Treprostinil | Treprostinil |
| hospital admission | Hospital admission |
| Bucetin, Kombinationen exkl. Psycholeptika | bucetin, combinations excl. psycholeptics |
| Morganella sp. | Morganella |
| Prasugrel | Prasugrel |
| Date of blood culture | Date of blood culture |
| Serratia sp. | Serratia |
| Anilide | Anilides |
| Cilostazol | Cilostazol |
| Histological finding | Histological finding |
| Paracetamol | Paracetamol |
| Phenacetin | Phenacetin |
| one-sided | Unilateral |
| Ticagrelor | Ticagrelor |
| Immunocompromise after radiation, chemotherapy and other immunosuppressive | Immune compromise after radiation, chemotherapy and other immunosuppressive |

| | |
|---|---|
| measures | measures |
| Other specified diseases of liver | Other specified diseases of liver |
| Bucetin | bucetin; inhalant, oral, rectal |
| Cangrelor | Cangrelor |
| Propacetamol | propacetamol; parenteral |
| Vorapaxar | Vorapaxar |
| Tracheostomy Cuff | Tracheostomy tube cuff |
| IMMUNGLOBULINE | Immunoglobulin |
| Paracetamol, Kombinationen exkl. Psycholeptika | paracetamol, combinations excl. psycholeptics; systemic |
| Selexipag | Selexipag |
| Change of dressing | Change of dressing |
| Transfer from Skilled Nursing Facility | Transfer from Skilled Nursing Facility |
| Immunglobuline, normal human | Immunoglobulins, normal human |
| Phenacetin, Kombinationen exkl. Psycholeptika | phenacetin, combinations excl. psycholeptics; inhalant, oral, rectal |
| Factor XIII | Factor XIII |
| Immunglobuline, normal human, zur extravasalen Anwendung | immunoglobulins, normal human, for extravascular adm.; systemic |
| History of myocardial infarction | History of myocardial infarction |
| Cerebrovascular accident | Cerebrovascular accident |
| Immunglobuline, normal human, zur intravasalen Anwendung | immunoglobulins, normal human, for intravascular adm.; systemic |
| Paracetamol, Kombinationen mit Psycholeptika | paracetamol, combinations with psycholeptics; systemic |
| Ear ossicle | Ossicle of ear |
| Ximelagatran | Ximelagatran |
| Phenacetin, Kombinationen mit Psycholeptika | phenacetin, combinations with psycholeptics; inhalant, oral, rectal |
| Haut | Haut |
| Anti-D(rh)-Immunglobulin | immunoglobulin anti-D(Rh) |
| Bucetin, Kombinationen mit Psycholeptika | bucetin, combinations with psycholeptics |
| Blood vessel | Blood vessel |
| Acetylsalicylsäure, Kombinationen mit Protonenpumpenhemmern | acetylsalicylic acid, combinations with proton pump inhibitors; systemic |
| Tetanus-Immunglobulin | Tetanus immunoglobulin |

| | |
|---|---|
| computed tomography | Computed tomography |
| Gamma-Glutamyl Transpeptidase | Gamma-glutamyl transpeptidase |
| pneuminia | Pneumonia |
| pulmonary arterial diastolic blood pressure | Pulmonary artery diastolic pressure |
| Swab finding | Swab finding |
| Body Temperature | Body temperature |
| biological sex | Biological sex |
| leukocyte | Leukocytes |
| pH value | pH |
| Therapy | Therapy |
| systolic blood pressure | Systolic blood pressure |
| lymphocyte | Lymphocyte |
| Medication | Medication |
| entlassungsart | Discharge type [CMS Assessment] |
| Complications | Complications |
| Imaging | Imaging |
| Symptoms | Symptoms |
| Vital signs | Vital signs |
| Ventilation therapy | Ventilation |
| Apheresis | Apheresis |
| ECMO therapy | ECMO |
| Not applicable (qualifier value) | Not applicable |
| No (qualifier value) | No |
| Yes (qualifier value) | Yes |
| Prone position | Prone position |
| Other (qualifier value) | Other |
| FiO2 | FIO2 - Inspired fraction of oxygen |
| Respiratory rate | Respiratory rate |
| Noninvasive ventilation | Noninvasive ventilation |
| Invasive ventilation (tracheostomy) | Invasive ventilation |
| Patient discharged alive | Discharged alive |
| Type of discharge | Discharge report |
| Intensive care | Intensive care |

| | |
|---|---|
| Heparin Kombinationen | heparin, combinations; systemic |
| Sulodexid | Sulodexide |
| Antithrombin III Antithrombin alfa | Antithrombin III |
| Acetylsalicylsäure Kombinationen mit Protonenpumpenhemmern | acetylsalicylic acid, combinations with proton pump inhibitors; systemic |
| Direct oral anticoagulants | Oral anticoagulants |
| Anticoagulation | Anticoagulation |
| ACE Inhibitors | ACE inhibitors, plain |
| Biological sex | Biological sex (property) |
| Male | Male |
| Female | Female |
| Neurology | Neurology |
| Psychiatry | Psychiatry |
| Neurological Symptoms | SYMPTOMS NEUROLOGICAL |
| Montreal Cognitive Assessment | Montreal Cognitive Assessment |
| Diagnose | Diagnosis |
| Untersuchungsbefund | Examination findings |
| Disorientation, unspecified | Disorientation, unspecified |
| Total Score | Total score |
| RASS | RASS - Richmond agitation-sedation scale |
| Modified Rankin Scale | Modified Rankin Scale |
| Radiology | Radiology |
| Befund | Findings |
| Klinische Angaben | Clinical data |
| Lungenparenchym/Atemwege | Lung parenchyma |
| Vergleich mit Untersuchung vom | Comparison study |
| Bronchiektasen | Bronchiectasis |
| Konsolidierungen | Consolidation |
| Ausdehnung/Severity des Lungenbefalls | Lung involvement stages |
| Kaverne | Cavern |
| Aufnahme-Status | Admission status |
| Intubiert | Intubated |
| Covid-19 therapy | COVID-19 Related Treatment |
| Immunoglobulins | Immunoglobulins |

| | |
|---|---|
| drug therapy | Drug therapy |
| Interferon alfa natürlich | interferon alfa natural; parenteral |
| Steroids | Steroids |
| immunoglobulins | Immunoglobulins |
| Methylprednisolon | methylprednisolon |
| Calcineurin inhibitor | Calcineurin inhibitor |
| Peginterferon alfa-2a Kombinationen | peginterferon alfa-2a, combinations; parenteral |
| Peginterferon alfa-2b Kombinationen | peginterferon alfa-2b, combinations; parenteral |
| Sirolimus | Sirolimus |
| Propyphenazon Kombinationen exkl. Psycholeptika** | phenazone, combinations excl. psycholeptics; oral |
| Calcineurin Inhibitoren | Calcineurin Inhibitors |
| Tumornekrosefaktor alpha(TNF alpha) Inhibitoren | Tumor necrosis factor alpha inhibitor |
| Interleukin 1 receptor antagonist | Interleukin 1 receptor antagonist |
| Corticosteroide zur systemischen Anwendung Kombinationen | Corticosteroids for systemic use, combinations |
| Colchicine | Colchicine |
| Paracetamol Kombinationen exkl. Psycholeptika | paracetamol, combinations excl. psycholeptics; systemic |
| Bucetin Kombinationen exkl. Psycholeptika | bucetin, combinations excl. psycholeptics |
| Phenacetin Kombinationen exkl. Psycholeptika | phenacetin, combinations excl. psycholeptics; inhalant, oral, rectal |
| Product containing zinc (medicinal product) | Zinc-containing product |
| Darunavir und Cobicistat | Darunavir + Cobicistat |
| Bucetin Kombinationen mit Psycholeptika | bucetin, combinations with psycholeptics |
| Atazanavir und Ritonavir | ATAZANAVIR AND RITONAVIR |
| Phenacetin Kombinationen mit Psycholeptika | phenacetin, combinations with psycholeptics; inhalant, oral, rectal |
| Chloroquine | Chloroquine |
| Ruxolitinib | Ruxolitinib |
| Convalescent plasma | Convalescent plasma |
| Calcifediol | Calcifediol |

| | |
|---|---|
| ace inhibitors | Ace inhibitors |
| Immunglobuline normal human | Immunoglobulins, normal human |
| Immunglobuline normal human zur extravasalen Anwendung | immunoglobulins, normal human, for extravascular adm.; systemic |
| Immunglobuline normal human zur intravasalen Anwendung | immunoglobulins, normal human, for intravascular adm.; systemic |
| Patient referral (procedure) | Patient referral |
| Referral to palliative care service (procedure) | Referral to palliative care service |
| pneumonia | Pneumonia |
| Neutrophils | Neutrophils |
| Fibrosis of lung | Fibrosis of lung |
| Coronary arteriosclerosis | Coronary arteriosclerosis |
| Diabetes mellitus | Diabetes mellitus |
| Sleep apnea (disorder) | Sleep apnea |
| Cirrhosis of liver (disorder) | Cirrhosis of liver |
| Pulmonary embolism (disorder) | Pulmonary embolism |
| Hypertensive disorder systemic arterial (disorder) | Hypertensive disorder, systemic arterial |
| Carotid artery stenosis (disorder) | Carotid artery stenosis |
| Dementia | Dementia |
| History of cerebrovascular accident with residual deficit (situation) | History of cerebrovascular accident with residual deficit |
| Multiple sclerosis (disorder) | Multiple sclerosis |
| Anxiety disorder (disorder) | Anxiety disorder |
| Psychotic disorder (disorder) | Psychotic disorder |
| Parkinson's disease (disorder) | Parkinson's disease |
| Epilepsy (disorder) | Epilepsy |
| severity | Severity |
| Moderate (severity modifier) (qualifier value) | Moderate |
| Severe (severity modifier) (qualifier value) | Severe |
| Diabetes type 2 | Diabetes mellitus type 2 |
| Diabetes type 1 | Diabetes mellitus type 1 |
| Obstructive sleep apnea syndrome | Obstructive sleep apnea syndrome (machine translation) |

| | |
|---|---|
| Bilirubin.total [Mass/volume] in Serum or Plasma | Bilirubin.total [Mass/volume] in Serum or Plasma |
| Albumin in Serum | Albumin in serum |
| INR | INR |
| Pregnant (finding) | Pregnant |
| IPSCurrentSmokingStatus | Smoking status |
| Unknown if ever smoked | Unknown if ever smoked |
| Former smoker | Former smoker |
| Non-smoker | Non-smoker |
| Yes | Yes |
| Peripheral arterial occlusive disease | Peripheral arterial occlusive disease |
| Heart failure | Heart failure |
| Cardiac arrhythmia | Cardiac arrhythmia |
| Critical illness polyneuropathy | Critical illness polyneuropathy |
| Fibrose und Zirrhose der Leber | Fibrosis and cirrhosis of liver |
| Pulmonary hypertension (disorder) | Pulmonary hypertension |
| Inflammatory bowel disease | Inflammatory bowel disease |
| Vasculitis (disorder) | Vasculitis |
| Chronische Polyarthritis nicht näher bezeichnet : Nicht näher bezeichnete Lokalisation | Chronic polyarthritis |
| Rheumatoid arthritis | Rheumatoid arthritis |
| Complication | Complication |
| Meningitis (disorder) | Meningitis |
| Chronic liver diseases | Chronic liver disease |
| Venous thrombosis (disorder) | Venous thrombosis |
| Schlaganfall nicht als Blutung oder Infarkt bezeichnet | Stroke, not specified as haemorrhage or infarction |
| Cardiac arrhythmia (disorder) | Cardiac arrhythmia |
| Thrombosis (disorder) | Thrombosis |
| Sepsis | Sepsis |
| Myocardial infarction (disorder) | Myocardial infarction |
| Encephalitis (disorder) | Encephalitis |
| Infectious disease of lung (disorder) | Infectious disease of lung |
| Nephropathy induced by other drugs, | Nephropathy induced by other drugs, |

<table>
<tr><td>medicaments and biological substances</td><td>medicaments and biological substances</td></tr>
<tr><td>|Respiratory finding (finding)|</td><td>Respiratory finding</td></tr>
<tr><td>Chronic lung diseases</td><td>Chronic lung disease</td></tr>
<tr><td>No</td><td>No</td></tr>
<tr><td>Unknown</td><td>Unknown</td></tr>
<tr><td>Computed tomography</td><td>Computed tomography</td></tr>
<tr><td>vaccinations</td><td>Vaccinations</td></tr>
<tr><td>Tuberculosis (disorder)</td><td>Tuberculosis</td></tr>
<tr><td>chronic liver diseases</td><td>Chronic liver disease</td></tr>
<tr><td>Human immunodeficiency virus infection</td><td>Human immunodeficiency virus infection constitutional disease</td></tr>
<tr><td>History of being a tissue or organ recipient</td><td>History of being a tissue or organ recipient</td></tr>
<tr><td>Immunization status</td><td>Immunization status</td></tr>
<tr><td>Disorders of cardiovascular system</td><td>Disorder of cardiovascular system</td></tr>
<tr><td>Tobacco smoking status</td><td>Tobacco smoking status</td></tr>
<tr><td>Respiratory therapy</td><td>Respiratory therapy</td></tr>
<tr><td>Chronic kidney diseases</td><td>Chronic kidney disease</td></tr>
<tr><td>Malignant neoplastic diseases</td><td>Malignant neoplastic disease</td></tr>
<tr><td>Autoimmune liver disease (disorder)</td><td>Autoimmune liver disease</td></tr>
<tr><td>Lung</td><td>Lung</td></tr>
<tr><td>Cerebral meninges structure (body structure)</td><td>Cerebral meninges structure</td></tr>
<tr><td>Liver</td><td>Liver</td></tr>
<tr><td>Skin part (body structure)</td><td>Skin part</td></tr>
<tr><td>Ear ossicle structure (body structure)</td><td>Ear ossicle structure</td></tr>
<tr><td>Tendon</td><td>Tendon</td></tr>
<tr><td>Cartilage tissue (body structure)</td><td>Cartilage tissue</td></tr>
<tr><td>Entire large intestine (body structure)</td><td>Entire large intestine</td></tr>
<tr><td>Chronic kidney disease stage 5 (disorder)</td><td>Chronic kidney disease stage 5</td></tr>
<tr><td>Pregnancy</td><td>Pregnancy</td></tr>
<tr><td>Date of birth</td><td>Date of birth</td></tr>
<tr><td>Ethnic group</td><td>Ethnic group</td></tr>
<tr><td>Weight</td><td>Body weight</td></tr>
<tr><td>Latino</td><td>Latino</td></tr>
<tr><td>Caucasian (ethnic group)</td><td>Caucasian</td></tr>
</table>

| | |
|---|---|
| Insulin treated type 2 diabetes mellitus (disorder) | Insulin treated type 2 diabetes mellitus |
| Lactate [Mass/volume] in Cerebral spinal fluid | Lactate [Mass/volume] in Cerebral spinal fluid |
| Disorder of cardiovascular system (disorder) | Disorder of cardiovascular system |
| Severity | Severity |
| Partial thromboplastin time | Partial thromboplastin time |
| Antithrombin | Antithrombin |
| Lymphocytes | Lymphocytes |
| Leukocytes | Leukocytes |
| Hemoglobin | Hemoglobin |
| Lactate | Lactate |
| Creatinine | Creatinine |
| Ferritin | Ferritin |
| Bilirubin | Bilirubin |
| Fibrinogen | Fibrinogen |
| Natriuretic peptide.B prohormone N-Terminal | Natriuretic peptide.B prohormone N-Terminal |
| Interleukin 6 | Interleukin 6 |
| Procalcitonin | Procalcitonin |
| CRP | CRP |
| Aspartate aminotransferase | Aspartate aminotransferase |
| D-dimer | D-dimer |
| Gamma glutamyl transferase | Gamma glutamyl transferase |
| Lactate dehydrogenase | Lactate dehydrogenase |
| PaO2 | Arterial partial pressure of oxygen |
| PaCO2 | Arterial partial pressure of carbon dioxide |
| Carbon dioxide [Partial pressure] in Capillary blood | Carbon dioxide [Partial pressure] in Capillary blood |
| Lactate [Mass/volume] in Arterial blood | Lactate [Mass/volume] in Arterial blood |
| Troponin T.cardiac [Mass/volume] in Serum or Plasma by High sensitivity method | Troponin T.cardiac [Mass/volume] in Serum or Plasma by High sensitivity method |
| Platelets | Platelets |
| Other symptoms | Other symptoms |
| Fatigue (finding) | Fatigue |

| | |
|---|---|
| Cough | Cough |
| Productive cough (finding) | Productive cough |
| Fever | Fever |
| Loss of taste | Loss of taste |
| Clouded consciousness | Clouded consciousness |
| Asymptomatic (finding) | Asymptomatic |
| Wheezing (finding) | Wheezing |
| Loss of sense of smell (finding) | Loss of sense of smell |
| Chest pain (finding) | Chest pain |
| Lymphadenopathy (disorder) | Lymphadenopathy |
| Vomiting | Vomiting |
| Joint pain (finding) | Joint pain |
| Hemoptysis (finding) | Hemoptysis |
| Body temperature | Body temperature |
| Heart rate | Heart rate |
| SOFA-Score | Sequential organ failure assessment score |
| Systolic blood pressure | Systolic blood pressure |
| Diastolic blood pressure | Diastolic blood pressure |
| Peripheral oxygen saturation | Peripheral oxygen saturation |
| Oxygen saturation in Arterial blood | Oxygen saturation in Arterial blood |
| pH of Arterial blood | pH of Arterial blood |
| Microorganism | Microorganism |
| Hospital admission (procedure) | Hospital admission |
| Prophylaxis - procedure intent (qualifier value) | Prophylaxis - procedure intent |
| \|Oxygen administration by nasal cannula (procedure)\|:\|Using access device (attribute)\|=\|High flow oxygen nasal cannula (physical object)\| | Oxygen administration by nasal cannula |
| New (intermittent) non-invasive ventilation | Non-invasive ventilation |
| New (intermittent) invasive ventilation | Invasive ventilation |
| Volume assist-control ventilation | Volume controlled ventilation |
| Ventilator Weaning | Ventilator Weaning |
| Reason for visit | Reason for visit diagnosis |
| discouragement | Feeling of discouragement |

| | |
|---|---|
| confirmed diagnosis | Confirmed diagnosis |
| Death | Death |
| Dead (finding) | Dead |
| Referral to palliative care service | Referral to palliative care service |
| Intensive Care Unit | Intensive care unit |
| Urgent Care Center | Urgent care center |
| Psychology | Psychology |
| Perinatology | Perinatology |
| hematology and oncology | Hematology / Oncology |
| Occupational Medicine | Occupational medicine |
| Pharmacology | Pharmacology |
| Addiction Medicine | Addiction Medicine |
| Bariatric Medicine | Obesity (Bariatric) Medicine |
| Laboratory Medicine | Clinical Pathology / Laboratory Medicine |
| Pulmonary Medicine | Pulmonary medicine |
| Hematology | Hematology |
| social service | Social service |
| Hand Surgery | Hand Surgery |
| Cardiothoracic surgery | Cardiothoracic surgery |
| Osteopathy | Osteopathy |
| Preventive Medicine | Preventive Medicine |
| Allergy and Immunology | Allergy and Immunology |
| Bariatric Surgery | Bariatric surgery |
| Podiatry | Podiatry |
| Osteopathic Medicine | Osteopathic medicine |
| Infectious diseases department | Infectious diseases department |
| Sleep Medicine Specialty | Sleep Medicine |
| Geriatric medicine | Geriatric medicine |
| Nursing care coordination | Nursing care coordination |
| Child Psychiatry | Child psychiatry service |
| General Surgery | General Surgery |
| Reconstructive Plastic Surgery | Plastic And Reconstructive Surgery |
| Cardiac surgery department | Cardiac surgery department |

| | |
|---|---|
| Nephrology | Nephrology |
| Sports Medicine | Sports Medicine |
| Cardiology | Cardiology |
| Anesthesiology | Anesthesiology |
| Pediatric Pulmonary Medicine | Pediatric pulmonology |
| General Practice | General Practice |
| Pathology | Pathology |
| Gastroenterology | Gastroenterology |
| family medicine | Family Medicine |
| Microbiology | Microbiology |
| Pain management (specialty) | Pain management (specialty) |
| Audiology | Audiology |
| Surgical Specialty | Surgical specialty |
| Pediatric nephrology | Pediatric Nephrology |
| Medical Genetics | Medical genetics |
| Speech Pathology | Speech Language Pathology |
| Rheumatology | Rheumatology |
| Orthopedics | Orthopedics |
| Integrative Medicine | Integrative medicine |
| Professional / ancillary services care | Professional / ancillary services care |
| Neonatology | Neonatology |
| Hospital Medicine | Hospital Medicine |
| Pediatric ophthalmology | Pediatric ophthalmology |
| Oncology | Oncology |
| Adolescent Medicine | Adolescent Medicine |
| Pediatrics | Pediatrics |
| Colorectal Surgery | Colorectal surgery |
| Palliative Medicine | Palliative medicine |
| Geriatric Psychiatry | Geriatric Psychiatry |
| Nursing | Nursing |
| Endocrinology | Endocrinology |
| Bone tissue | Bone tissue |
| Skin | Skin |

| | |
|---|---|
| Tendon structure (body structure) | Tendon structure |
| Entire small intestine (body structure) | Entire small intestine |
| Interventional cardiology | Interventional cardiology |
| Virology | Virology |
| bacteriology | Bacteriology - general |
| Microbiological diagnostics | Microbiological method |
| laboratory finding | Laboratory finding |
| Osmotic Fragility | Osmotic Fragility |
| Behavioral health | Behavioral health |
| Pediatric rheumatology | Pediatric Rheumatology |
| Pediatric Oncology | Pediatric oncology |
| Pediatric Hematology/Oncology | Pediatric hematology oncology |
| Pediatric Neurology | Pediatric neurology |
| Pediatric Surgery | Pediatric surgery |
| Pediatric cardiology | Pediatric cardiology |
| Diagnostic Radiology | Diagnostic Radiology |
| Nuclear Medicine | Nuclear Medicine |
| Postobstructive pneumonia | Postobstructive pneumonia |
| Dyspnea | Dyspnea |
| Worries Overwhelm Me | My worries overwhelmed me in past 7 days |
| Feel Restless | Feel restless |
| Fair | General Quality: Fair |
| Very Poor | Very poor |
| Short of breath dressing/undressing | Short of breath dressing/undressing |
| Nadroparin | Nadroparin |
| Reviparin | Reviparin |
| Parnaparin | parnaparin |
| Direkte Faktor Xa Inhibitoren | Factor Xa Inhibitors |
| Encounter join Identifier | Encounter identifier |
| home visit care | Care home visit |
| End-Stage Renal Disease Treatment Facility | End-Stage Renal Disease Treatment Facility |
| Assisted Living Facility | Assisted living facility |
| Long-Term Care | Long-term care service |

| | |
|---|---|
| Temporary Lodging | Temporary Lodging |
| Hospice | Hospice |
| Dialysis Center | Dialysis facility/center |
| rural health clinic | Rural Health Clinic |
| Pharmacy visit | Pharmacy visit |
| Land ambulance | Land ambulance |
| Residential Substance Abuse Treatment Facility | Residential Substance Abuse Treatment Facility |
| Comprehensive Outpatient Rehabilitation Facility | Comprehensive Outpatient Rehabilitation Facility |
| Clinic | Clinic |
| Skilled Nursing Facility | Skilled nursing facility |
| Telemedicine | Telemedicine - General |
| nursing home visit | Nursing home visit |
| Urgent hospital admission | Urgent hospital admission |
| Ambulatory Surgical Center | Ambulatory Surgical Center |
| mobile medical service | Mobile medical facility |
| Inpatient rehabilitation facility | Inpatient rehabilitation facility |
| Federally Qualified Health Center | Federally Qualified Health Center |
| Inpatient Psychiatric Facility | Inpatient Psychiatric Facility |
| Comprehensive Inpatient Rehabilitation Facility | Comprehensive Inpatient Rehabilitation Facility |
| Homeless Shelter | Homeless Shelter |
| Observation Duration | Observation duration |
| Encounter record identifier hash | Encounter identifier |
| Nursing Assistant | Nursing assistant |
| Respiratory Therapist | Respiratory therapist |
| Students, Nursing | Student nurse |
| Advanced Practice Nurse | Advanced Practice Nurse (APN) |
| psychologist | Psychologist |
| Other student | Student |
| technician | Technician |
| Fellow | Fellow |
| Teacher | Teacher training teacher |

| | |
|---|---|
| Social Worker | Social worker |
| Pharmacist | Pharmacist specialist |
| Chiropractor | Chiropractor |
| Dentist | Dentist |
| Total duration | Test duration |
| drug category | Has controlled drug category (DM+D) |
| Generic package join identifier | Generic Product Identifier |
| Ingredient - RxNorm RXCUI:ID:Pt:Medication:Nom | Ingredient - RxNorm RXCUI |
| Antipyretics | ANTIPYRETICS |
| Interleukin Inhibitoren | Interleukin inhibitors |
| acyclovir | acyclovir |
| Hydroxychloroquine | Hydroxychloroquine |
| Search type | Search procedure |
| record identifier | Identifier |
| Migraine (disorder) | Migraine |
| Asthma (disorder) | Asthma (disorder) |
| Chronic obstructive lung disease (disorder) | Chronic obstructive lung disease |
| Kardiale Arrhythmie nicht näher bezeichnet | Cardiac arrhythmia, unspecified |
| Cirrhosis of liver | Cirrhosis of liver |
| Fibrosis of lung (disorder) | Fibrosis of lung |
| Sleep apnea | Sleep apnea |
| Multiple sclerosis | Multiple sclerosis |
| Life threatening severity | Life threatening severity |
| Medium | Medium |
| Persistent | Persistent |
| Moderate to severe (qualifier value) | Moderate to severe |
| Intermittent | Intermittent |
| Current every day smoker | Current every day smoker |
| Chronic Systemic Steroid Use | On long term systemic steroid therapy |
| Diagnostic Procedure | Diagnostic procedure |
| Biospecimen | General biological sample |
| Neurologic Examination | Neurologic examination |
| ABO Blood Group Determination | ABO antigen type determination |

| | |
|---|---|
| serology | Serology |
| diagnosis | Diagnosis |
| sputum examination | Sputum examination |
| echocardiogram | Echocardiogram |
| Immunofixation | Immunofixation |
| Point of care testing | Point of care testing |
| immunophenotyping | Immunophenotyping |
| electrocardiogram | Biofeedback, electrocardiogram |
| Auscultation of lower respiratory tract | Auscultation of lower respiratory tract |
| COVID-19 Diagnosis | COVID-19 infection |
| is principal | Principal |
| swab specimen | Swab specimen |
| throat swab collection | Taking throat swab |
| date of specimen collection | Specimen collection date |
| induced sputum specimen | Collection of induced sputum |
| Bronchoalveolar Lavage Fluid | Bronchoalveolar lavage fluid sample |
| rectal swab specimen | Rectal swab |
| Urine volume 24 hour | 24 hour urine output |
| Sputum culture | Sputum culture |
| No bacterial detection | No bacteria found |
| mitral valve insufficiency | Mitral Valve Insufficiency |
| Right ventricular strain pattern | Electrocardiogram (EKG): Right ventricular strain |
| Aortic Valve Insufficiency | Aortic (valve) insufficiency |
| Date of echocardiogram | Date of echocardiogram |
| mitral valve stenosis | Mitral valve stenosis |
| Urine immunofixation | Urine immunofixation |
| Charlson Comorbidity Index | Charlson Comorbidity Index |
| CURB-65 | CURB-65 (confusion, urea, respiratory rate, blood pressure, 65 years of age or older) score |
| Barthel Index of Activities of Daily Living | Barthel Index of Activities of Daily Living score |
| APACHE II - Total APACHE II Score | Apache II score |
| Tobacco Use | Tobacco use |

| | |
|---|---|
| alcohol use | Alcohol use |
| Stop | Stop |
| passive | Passive |
| Sedation | Sedation |
| electrode | Cystoscopic electrode |
| Electrocardiographic Finding | Electrocardiographic axis finding |
| Atrial Fibrillation by ECG Finding | ECG: atrial fibrillation |
| Wright-Giemsa stain | Wright-Giemsa stain |
| Blood specimen with EDTA | Blood specimen with EDTA |
| Smear Specimen | Nasal smear specimen |
| pleural fluid | Pleural fluid |
| Fresh Frozen Plasma | Fresh frozen plasma |
| Blood Product | Blood product |
| blood plasma | Blood plasma |
| cell specimen | Cell culture specimen |
| virology material | Virology |
| lung disease | Lung Disease |
| Myocarditis | Myocarditis |
| Other thrombosis or embolism | Other embolism and thrombosis |
| Myocardial infarction | Myocardial infarction |
| Other lung disease | Other lung disease NEC |
| Ventricular tachycardia | Ventricular tachycardia ablation |
| Anaemia in other chronic diseases classified elsewhere | Anemia in other chronic diseases classified elsewhere |
| Enema Administration | Enema administration assessment |
| Paracentesis | Paracentesis, Abdominal Paracentesis |
| SNOMED CT Identifier | SNOMED CT integer identifier |
| Manufacturer name | Manufacturer name |
| pneumothorax | Pneumothorax |
| Disease caused by severe acute respiratory syndrome coronavirus 2 (disorder) | Disease caused by Severe acute respiratory syndrome coronavirus 2 absent |
| Influenza A/B Antigen Measurement | Influenza B antigen level |
| Direct Fluorescent Antibody Test | Direct fluorescent antibody test for syphilis |
| Quite a Bit | Quite a bit |

| | |
|---|---|
| Remission phase (qualifier value) | Remission phase |
| acquired immunodeficiency syndrome | Acquired Immunodeficiency Syndrome |
| Secondary diabetes mellitus (disorder) | Secondary diabetes mellitus |
| serum glucose level | Serum glucose level |
| Coronary arteriosclerosis (disorder) | Coronary arteriosclerosis |
| \|History of clinical finding in subject (situation)\|:\|Associated finding (attribute)\|=\|Myocardial infarction (disorder)\| | History of clinical finding in subject |
| Receiving chemotherapy | Currently receiving chemotherapy for cancer |
| Delivery date | Delivery date |
| Finger test (sacral area) | Finger tapping test |
| apartment number | Current apartment number [Location] [PhenX] |
| Other public insurance (CHIP) | Other public insurance (CHIP) |
| Arabs (ethnic group) | Arabs |
| Caucasian | Caucasian |
| Feeding Tube | Enteral Feeding Tube |
| Catheter Site | Catheter tip site |
| Personal history of contact with and (suspected) exposure to potentially hazardous body fluids | Personal history of contact with and (suspected) exposure to potentially hazardous body fluids |
| Travel in the same means of transport (distance less than 2m) | Person in the transport environment |
| Household Contact with SARS-CoV-2 Positive Individual | Household contact with confirmed COVID-19 case patient |
| health personnel | Public health personnel |
| Systematized Nomenclature of Medicine | Systematic Nomenclature of Medicine - Clinical Terms (IHTSDO) |
| Logical Observation Identifiers Names and Codes | Logical Observation Identifiers Names and Code |
| Source information | Source of information |
| LOINC hierarchy | LOINC Hierarchy |
| Attribute value | Attribute value |
| Unique Identifier | Unique identifier |
| attribute | Attribute |
| Primitive | Neuroectodermal Tumors, Primitive |

| | |
|---|---|
| SNOMED CT Concept | SNOMED CT |
| legacy identifier | Legacy case identifier |
| depth | Depth |
| Ask at order entry | Ask at order entry |
| time | Time |
| Document section | Document section |
| definition | Definition |
| Property | Property |
| Test Name | Test name |
| system | system |
| Method | Method |
| The Unified Code for Units of Measure | Unified Code for Units of Measure (Regenstrief Institute) |
| Time aspect | Time aspect of |
| scale type | Scale type |
| Clinical Class | Clinical Class |
| Survey | Survey |
| Laboratory Class | Laboratory Class |
| Respiratory Tract Diseases | Respiratory Tract Diseases |
| History of cerebrovascular accident without residual deficits (situation) | History of cerebrovascular accident without residual deficits |
| acute renal failure | Acute renal failure |
| cytopenia | Cytopenia |
| Large unstained cells | Large unstained cells |
| Plasminogen | Plasminogen |
| prostaglandin | Prostaglandin |
| 24 hour urine protein assay | Urine estriol 24 hour assay |
| Oxygen [Partial pressure] in Arterial blood | Oxygen [Partial pressure] in Arterial blood |
| Carbon dioxide [Partial pressure] in Arterial blood | Carbon dioxide [Partial pressure] in Arterial blood |
| 24 hour urine creatinine assay | 24 hour urine creatinine output |
| Calcium urine (24 hour sample) | 24 hour urine calcium output |
| Bicarbonate:SCnc:Pt:BldA:Qn | Bicarbonate |
| Base excess:SCnc:Pt:BldA:Qn:Calculated | Base excess |

| | |
|---|---|
| Base excess:SCnc:Pt:BldC:Qn:Calculated | Base excess |
| Clinical referral | Clinical referral |
| Abdominal pain (finding) | Abdominal pain |
| Nausea (finding) | Nausea |
| disturbances of sensation of smell and taste | Disturbances of sensation of smell and taste |
| Hardly at All | Hardly at all |
| Room Air | Room air |
| Thrombosis prophylaxis or anticoagulation or platelet aggregation inhibition | Platelet aggregation inhibitor prophylaxis |
| Entire cornea (body structure) | Entire cornea |
| Propyphenazon Kombinationen exkl. Psycholeptika | Propyphenazon |
| Atazanavir und Cobicistat | Atazanavir + Cobicistat |
| Lopinavir and Ritonavir | lopinavir and ritonavir; oral |
| Tumor necrosis factor alpha inhibitor | Tumor necrosis factor alpha inhibitor |
| Remdesivir | Remdesivir |
| Inflammatory bowel disease (disorder) | Inflammatory bowel disease |
| Mild to moderate (qualifier value) | Mild |
| Embolism (disorder)" | Embolism |
| Venous thrombosis | Venous thrombosis |
| Cerebrovascular accident (disorder) | Cerebrovascular accident |
| Chronic viral hepatitis (disorder) | Chronic viral hepatitis |
| Total serum bilirubin 12 mg per dl | Total bilirubin (& level) |
| Serum creatinine 2 mg per dl or use of Terlipressin | Serum creatinine level - finding |
| Prior ventilation or oxygen support | Ventilation support |
| Extreme obesity with alveolar hypoventilation (disorder) | Extreme obesity with alveolar hypoventilation |
| Chronic kidney disease stage 5 on dialysis (disorder) | Chronic kidney disease stage 5 |
| Chronic kidney disease stage 1 (disorder) | Chronic kidney disease stage 1 |
| Chronic kidney disease stage 2 (disorder) | Chronic kidney disease stage 2 |
| Chronische Nierenkrankheit Stadium 3 | Chronic kidney disease, stage 3 |
| Dementia (disorder) | Dementia |
| Anxiety disorder | Anxiety disorder |

| | |
|---|---|
| Carotid artery stenosis | Carotid artery stenosis |
| hospitalized with mild disease - oxygen by mask or nasal prongs | Hospitalized - mild disease, oxygen by mask or nasal prongs |
| hospitalized with severe disease - intubation and mechanical ventilation | Hospitalized - severe disease, intubation and mechanical ventilation |
| hospitalized with severe disease - non-invasive ventilation or high-flow oxygen | Hospitalized - severe disease, non-invasive ventilation or high-flow oxygen |
| hospitalized with severe disease - ventilation and additional organ support - pressors, RRT, ECMO | Hospitalized - severe disease, ventilation and additional organ support - pressors, RRT, ECMO |
| hospitalized with mild disease - no oxygen therapy | Hospitalized - mild disease, no oxygen therapy |
| Pneumococcal infectious disease (disorder) | Pneumococcal infectious disease |
| Cefpodoxim or Clavulanic acid | Clavulanic acid |
| Diabetes mellitus type 1 (disorder) | Type 1 diabetes mellitus |
| Other cardiovascular disease present | Other cardiovascular disease |
| Body weight (observable entity) | Body weight |
| Asian ethnic group (ethnic group) | Asian - ethnic group |
| Other ethnic mixed origin (ethnic group) | Other ethnic, mixed origin |
| Pneumocystis jirovecii PCR from BAL or respiratory sample | Pneumocystis jirovecii |
| Loss of taste (finding) | Loss of taste |
| Vomiting (disorder) | Vomiting |
| Body temperature:Temp:Pt:Intravascular:Qn | Body temperature |

## Supplementary Table 4: Hospitals

| Name | Address |
|---|---|
| UK Aachen | Pauwelsstraße 30, 52074 Aachen |
| Universitätsklinikum Frankfurt | Theodor-Stern-Kai 7, 60590 Frankfurt am Main |
| UK Erlangen | Maximilianspl. 2, 91054 Erlangen |

## Supplementary Text 1: Resources for CDM

We added variables to the data model inspired by multiple data sources to create a sufficient data model for the data we had access to. Those were: LEOSS [https://leoss.net/data/], MIMIC-III [https://mimic.mit.edu/ ], MC-19 [https://clinicaltrials.gov/ct2/show/record/NCT04387799 ], CAPNETZ [https://capnetz.de/html/research/covid19 ], IBM Explorys Therapeutic Datasets [https://www.ibm.com/products/explorys-ehr-data-analysis-tools ], COVID-19 data sets from the University of Erlangen, University of Frankfurt and RWTH Aachen.

## Supplementary Text 2: Example of accepting a hypothesis in a larger cohort

We have two data sets A and B where |A| = 600 patients and |B| = 200 patients.
In each cohort, we test a drug to treat fever in COVID-19 ICU patients.
In trial A we have the following results:

| | Fever went down | Fever stayed | Total |
|---|---|---|---|
| Group 1 (Drug) | 174 | 126 | 300 |
| Group 2 (Control) | 150 | 150 | 300 |
| | 324 | 276 | 600 |

With Fisher's exact test we have **p = 0.0595** and cannot reject the hypothesis that the drug has no effect on the patient.
In the second cohort (B) we have the results :

| | Fever went down | Fever stayed | Total |
|---|---|---|---|
| Group 1 (Drug) | 59 | 41 | 100 |
| Group 2 (Control) | 45 | 55 | 100 |
| Sum | 104 | 96 | 200 |

Again with Fisher's exact test, we have **p = 0.0655** which is again not significant.

But now we use the CDM to standardize the cohort data and get a larger cohort with 800 patients and have the results.

| | Fever went down | Fever stayed | Total |
|---|---|---|---|
| Group 1 (Drug) | 233 | 167 | 400 |

| | | | |
|---|---|---|---|
| Group 2 (Control) | 195 | 205 | 400 |
| Sum | 428 | 372 | 800 |

And if we perform Fisher's exact test to test the hypothesis we get **p=0.0087** which is very significant and we can reject our hypothesis that the drug has no effect on the patient's fever.

## Supplementary Text 3: Collecting heterogeneous data sets with the DST

The work by *Wegner et al* (https://arxiv.org/abs/2111.09313) how multiple data source are collected into one common system with the help of common data models like the one presented in this paper.

## Supplementary Text 4: Similar work

Other work that has been done toward modeling COVID-19 data is cloesely related to the OMOP model. Like those:
https://www.medrxiv.org/content/10.1101/2021.11.23.21266734v1,
https://pubmed.ncbi.nlm.nih.gov/33887457/,
https://pubmed.ncbi.nlm.nih.gov/35077901/
Hence it is a crucial part of this work that firstly we mapped the model presented here to OMOP and secondly curated those mappings by domain experts.
